# Real-World Antidepressant Prescribing Patterns and Comparative Safety Outcomes of Commonly Prescribed Antidepressants in Patients with Depressive Disorder: A Multicenter OMOP-CDM Study

**DOI:** 10.3390/ph19091332

**Published:** 2026-08-24

**Authors:** Jungha Min, Sueun Shin, Sohyeon Park, Jeongha Yun, Kaylnn Park, Sandy Jeong Rhie

**Affiliations:** 1 College of Pharmacy, Ewha Womans University, Seoul 03760, Republic of Korea; 2090356@ewha.ac.kr (J.M.); 2164055@ewhain.net (S.S.); 2 Graduate School of Pharmaceutical Sciences, Ewha Womans University, Seoul 03760, Republic of Korea; qkrthgus@ewha.ac.kr (S.P.); jeongha.yun@ewha.ac.kr (J.Y.); ngpark93@ewha.ac.kr (K.P.)

**Keywords:** depressive disorder, antidepressants, prescribing patterns, real-world evidence, safety

## Abstract

**Background/Objectives**: Real-world antidepressant use for depressive disorder varies across clinical settings. This study aimed to characterize prescribing pathways and evaluate the comparative safety of frequently prescribed antidepressants in a large multicenter cohort with newly diagnosed depressive disorder. **Methods**: We conducted a retrospective multicenter observational study using electronic health record data standardized to the OMOP Common Data Model (1999–2026) in adults initiating antidepressants. Prescribing patterns among 13,381 patients were visualized using sunburst plots and Sankey diagrams. Comparative safety, escitalopram vs. sertraline and trazodone, was evaluated across 18 hospitals (*n* = 44,542) for nine clinical safety outcomes using propensity score-matched Cox proportional hazards models and empirically calibrated random-effects meta-analysis. **Results**: Selective serotonin reuptake inhibitors, particularly escitalopram (21.4%, *n* = 2868), were the preferred antidepressant. Common adjunctive medications included trazodone (14.6%, *n* = 794) and quetiapine (14.0%, *n* = 761), though utilization varied across hospitals. No clear differences were detected for most safety outcomes between cohorts. However, empirically calibrated QTc prolongation risk was significantly increased with sertraline relative to escitalopram (HR 1.75, 95% CI 1.10–2.78), a signal requiring further confirmation. **Conclusions**: This study demonstrates substantial institutional variation exists in real-world antidepressant treatment pathways and combination strategies. While comparative safety profiles were broadly comparable across evaluated outcomes, the isolated QTc prolongation signal for sertraline warrants targeted external confirmation. Furthermore, trazodone and escitalopram cohorts should not be regarded as clinically interchangeable first-line options, supporting individualized, evidence-based antidepressant selection in routine clinical practice.

## 1. Introduction

Depressive disorder is a common psychiatric condition associated with considerable morbidity and reduced quality of life, imposing a substantial burden on individuals and healthcare systems worldwide. Antidepressants are widely used to treat depressive disorder, and recent treatment strategies increasingly emphasize early adjunctive therapy, including antipsychotics, within stepwise treatment approaches [1]. Specifically, major clinical guidelines, such as those from the American Psychiatric Association (APA) and the Canadian Network for Mood and Anxiety Treatment (CANMAT), recommend a measurement-based, sequential treatment approach to optimize remission, which frequently involves drug rotation or adjunctive strategies when initial monotherapy fails [2,3].

Several antidepressants are available in clinical practice, including selective serotonin reuptake inhibitors (SSRIs), serotonin–norepinephrine reuptake inhibitors (SNRIs), noradrenergic and specific serotonergic antidepressants, serotonin antagonist and reuptake inhibitors, and tricyclic antidepressants (TCAs). Among these agents, SSRIs are commonly recommended as first-line pharmacologic therapy because of their safety and tolerability compared with older antidepressants, including TCAs and monoamine oxidase inhibitors (MAOIs) [4]. While TCAs, such as amitriptyline and imipramine, demonstrate robust clinical efficacy, their utility is heavily restricted by class-wide anticholinergic adverse effects, antihistaminic sedation, orthostatic hypotension, and cardiotoxicity, notably QTc prolongation and lethal arrhythmias in overdose. In contrast, newer-generation agents like SSRIs and SNRIs exhibit a safer therapeutic index. However, SNRIs, such as venlafaxine and duloxetine, are associated with dose-dependent blood pressure elevations due to noradrenergic activity, and NaSSAs, such as mirtazapine, frequently induce dose-limiting somnolence and weight gain [5]. In practice, real-world antidepressant treatment extends beyond SSRIs. In routine clinical practice, treatment selection and subsequent modifications vary based on patient characteristics, residual symptoms, comorbidities, clinician preferences, and institutional prescribing practices [6,7,8].

Comparative evidence on individual antidepressants, rather than drug classes alone, demonstrates differences in efficacy and tolerability. In a landmark network meta-analysis analyzing 21 active drugs, significant variations in clinical response and discontinuation rates were identified at the individual level, even among drugs within the same therapeutic class [5]. When examining these specific agents by class, SSRIs, such as escitalopram, have been associated with superior efficacy and early onset of action, combined with a favorable pharmacokinetic profile with a relatively low propensity for cytochrome P450-mediated drug-drug interactions, making it highly suitable for patients with polypharmacy [9]. Sertraline, another commonly prescribed SSRI, is noted for its high response rate and acceptability, though it carries a slightly elevated risk of early gastrointestinal side effects such as diarrhea [9]. Furthermore, SARIs such as trazodone are often utilized at lower doses (25–150 mg) for their sedative properties, acting via potent 5-HT2A and histaminergic H1 receptor antagonism; this has been reported to help manage comorbid sleep disturbances without dependence risk associated with traditional hypnotics [10]. At the class level, meta-analyses ranked antidepressants based on efficacy and acceptability [5]. However, real-world safety comparisons at the individual drug level, particularly those using standardized multicenter electronic health record (EHR) data, remain limited.

For example, a nationwide Danish register-based study demonstrates frequent changes in antidepressant treatment strategies during long-term follow-up, reflecting the complexity of routine antidepressant use [11]. Information on preferential antidepressant prescribing, treatment pathway changes over time, and differences in the safety of commonly prescribed agents may inform treatment selection in real-world clinical practice. However, previous studies have primarily evaluated prescribing trends at the antidepressant class level, such as SSRIs and SNRIs, rather than for individual drugs. This distinction is important because treatment decisions in clinical practice are based on specific antidepressants rather than therapeutic classes. Although antidepressants within the same class share pharmacological characteristics, individual agents may differ in prescribing patterns, treatment trajectories, tolerability, and safety.

Additionally, studies using standardized Observational Medical Outcomes Partnership Common Data Model (OMOP-CDM) data in South Korea primarily describe treatment pathways or evaluate safety outcomes associated with specific adverse events [12,13]. However, evidence integrating treatment pathways and comparative safety analyses of commonly prescribed antidepressants remains limited. In addressing this gap, multicenter evidence describing prescribing patterns, treatment modifications, routine clinical use, and comparative safety of specific antidepressants is needed to better inform antidepressant treatment decisions.

Therefore, this study aimed to characterize real-world antidepressant prescribing pathways and compare the safety outcomes of commonly prescribed antidepressants using multicenter OMOP-CDM data from South Korea.

## 2. Results

### 2.1. Sequential Antidepressant Treatment Patterns

A total of 13,381 patients across five tertiary hospitals were included in the treatment pathway analysis (AUMC [Ajou University Medical Center], *n* = 2676; EUMC [Ewha Womans University Medical Center], *n* = 2459; KDH [Kangdong Sacred Heart Hospital], *n* = 3234; KHNMC [Kyung Hee University Hospital at Gangdong], *n* = 628; KWMC [Kangwon National University Hospital], *n* = 4384).

#### 2.1.1. Initial Choice of Antidepressant

**Figure 1:**
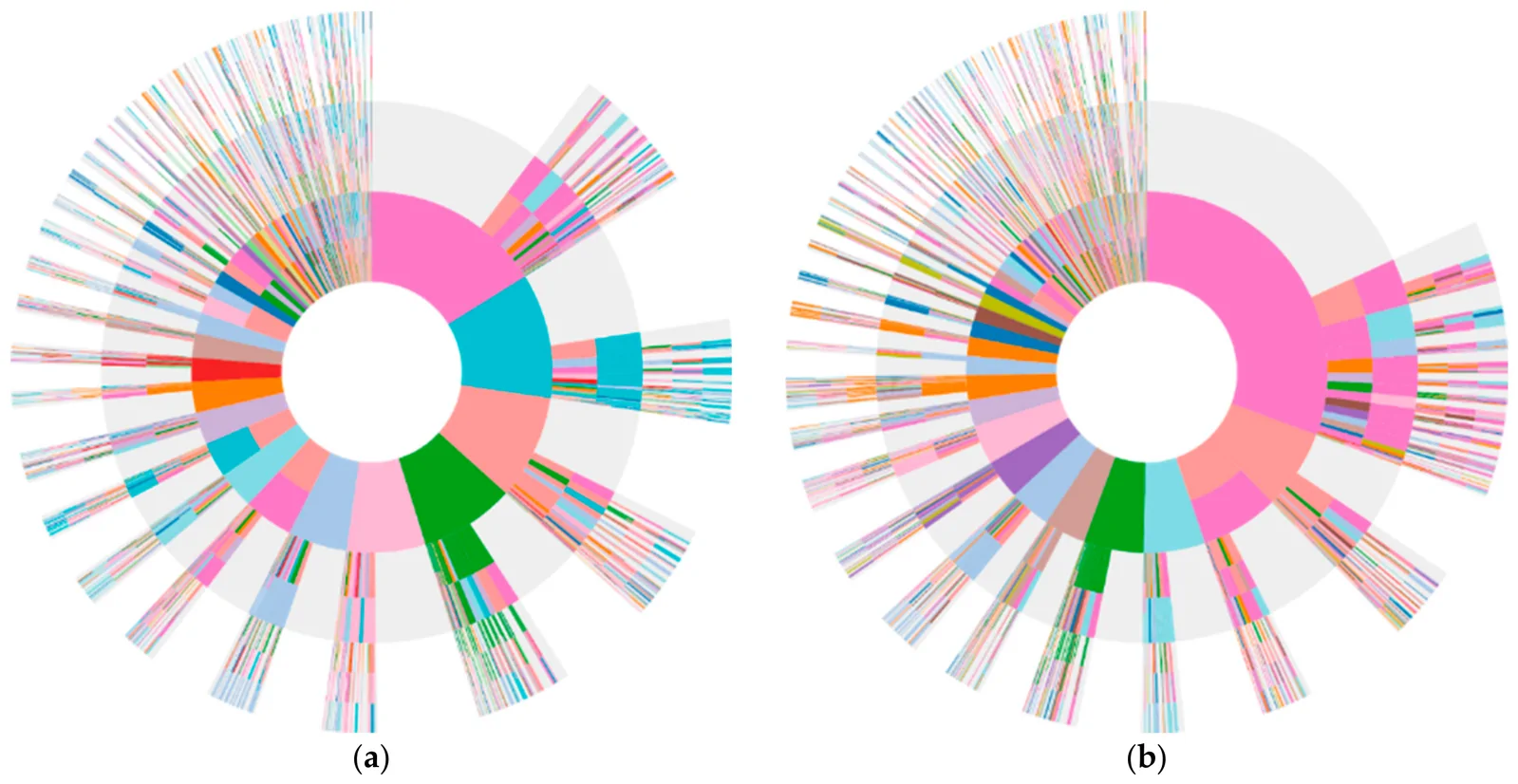

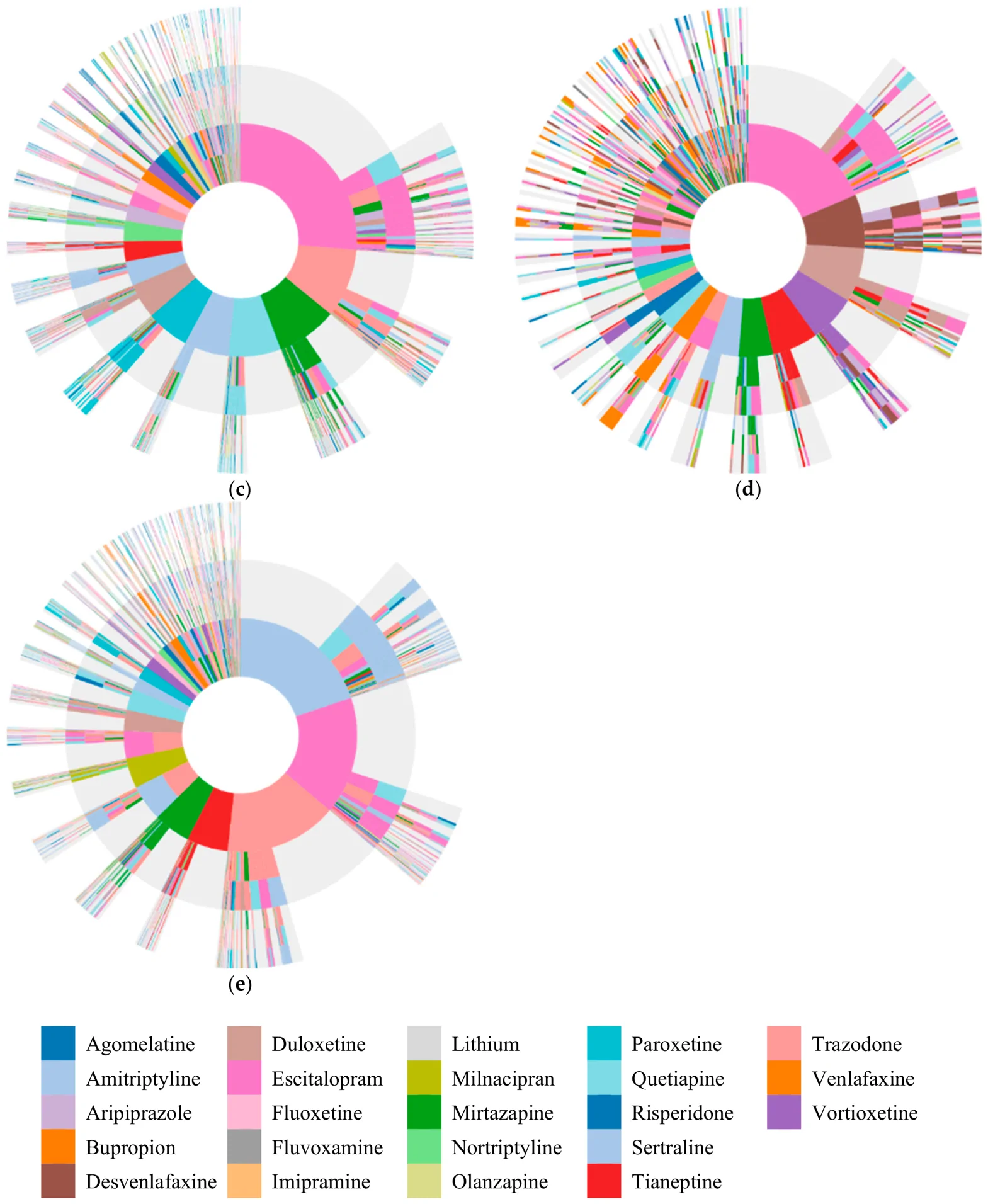
Sunburst plots. They illustrate antidepressant treatment pathways from STEP 1–3 across five tertiary hospitals (total patients, *n* = 13,381). The inner ring represents the initial antidepressant therapy (STEP 1), and the outer rings represent subsequent treatment pathways (STEP 2 and 3). Colors denote individual antidepressants. (**a**) AUMC: *n* = 2676, (**b**) EUMC: *n* = 2459, (**c**) KDH: *n* = 3234, (**d**) KHNMC: *n* = 628, (**e**) KWMC: *n* = 4384. Full-size versions of these plots are provided in Supplementary Figure S1. Abbreviations: AUMC, Ajou University Medical Center; EUMC, Ewha Womans University Medical Center; KDH, Kangdong Sacred Heart Hospital; KHNMC, Kyung Hee University Hospital at Gangdong; KWMC, Kangwon National University Hospital. Colors denote individual antidepressants: agomelatine, amitriptyline, aripiprazole, bupropion, desvenlafaxine, duloxetine, escitalopram, fluoxetine, fluvoxamine, imipramine, lithium, milnacipran, mirtazapine, nortriptyline, olanzapine, paroxetine, quetiapine, risperidone, sertraline, tianeptine, trazodone, venlafaxine, and vortioxetine.

SSRIs were the most frequently prescribed first-line antidepressants (innermost ring, Figure 1) across all five hospitals (*n* = 13,381). Among the SSRIs, escitalopram was the most commonly prescribed initial antidepressant, accounting for approximately 15–30% of first-line prescriptions in four hospitals (AUMC: *n* = 431, 16.2%; EUMC: *n* = 753, 30.5%; KDH: *n* = 852, 26.2%; and KHNMC: *n* = 117, 18.7%; Figure 1).

Following escitalopram, the second- and third-most frequently initiated antidepressants varied across hospitals: paroxetine (*n* = 299, 11.3%) and trazodone (*n* = 251, 9.5%) in AUMC; trazodone (*n* = 176, 7.1%) and the trazodone + escitalopram combination (*n* = 172, 7.0%) at EUMC; trazodone (*n* = 309, 9.5%) and mirtazapine (*n* = 273, 8.4%) at KDH; and desvenlafaxine (*n* = 47, 7.5%) and duloxetine (*n* = 47, 7.5%) at KHNMC. In contrast, KWMC showed a distinct prescribing preference for sertraline (*n* = 869, 19.6%) as the most frequently initiated antidepressant, followed by escitalopram (*n* = 715, 16.2%; Figure 1).

#### 2.1.2. Subsequent Treatment Pathways

Sunburst plots were used to visualize treatment pathways from initial antidepressant therapy (STEP 1) through subsequent treatment modifications to STEP 3 (Figure 1).

At STEP 2 (second ring from the center, Figure 1), a substantial proportion of patients remained on their initial antidepressant regimen without modification. Across all hospitals, commonly observed treatment modifications following escitalopram included the addition of trazodone, quetiapine, and other adjunctive agents; however, their relative frequencies varied across hospitals. For example, at EUMC (*n* = 2459), trazodone was added to escitalopram in 68 patients (2.7%), whereas at KDH (*n* = 3234), quetiapine was the most frequently added adjunctive agent (*n* = 75, 2.3%). Similarly, at AUMC (*n* = 2676), trazodone (*n* = 35, 1.3%) and quetiapine (*n* = 27, 1.0%) were the most commonly added adjunctive agents following escitalopram. At KHNMC (*n* = 628), tianeptine (*n* = 6, 0.96%) and trazodone (*n* = 4, 0.6%) were the most frequently added agents. At KWMC (*n* = 4384), quetiapine (*n* = 106, 2.4%) and trazodone (*n* = 82, 1.84%) were also commonly added. These findings highlight substantial interhospital variability in treatment modification strategies. In addition to the commonly added agents, a wide range of other medications was observed at lower frequencies. These included antidepressants such as sertraline, bupropion, and fluoxetine, as well as adjunctive agents including aripiprazole and amitriptyline, each accounting for generally < 1% of treatment modifications across hospitals.

At STEP 3 (third ring from the center, Figure 1), a broader range of medications was observed, including antidepressants, antipsychotics, and other adjunctive agents, reflecting the complexity of later-stage treatment strategies.

In the two hospitals with sufficient case volumes for period-stratified analysis (KDH and KWMC), prescribing patterns before and after 2017 differed substantially: milnacipran use at KWMC declined after 2017 as sertraline emerged as the predominant first-line agent, and vortioxetine emerged at KDH as a newly used option among patients aged ≥ 65 years (Supplementary Figure S2). Age- and sex-stratified sunburst analyses at these two hospitals did not reveal notable differences in treatment pathway patterns; the sequence and relative frequency of initial and subsequent antidepressant regimens were broadly similar across age (<65 vs. ≥65 years) and sex (male vs. female) strata at both hospitals.

#### 2.1.3. Transition Dynamics

**Figure 2:**
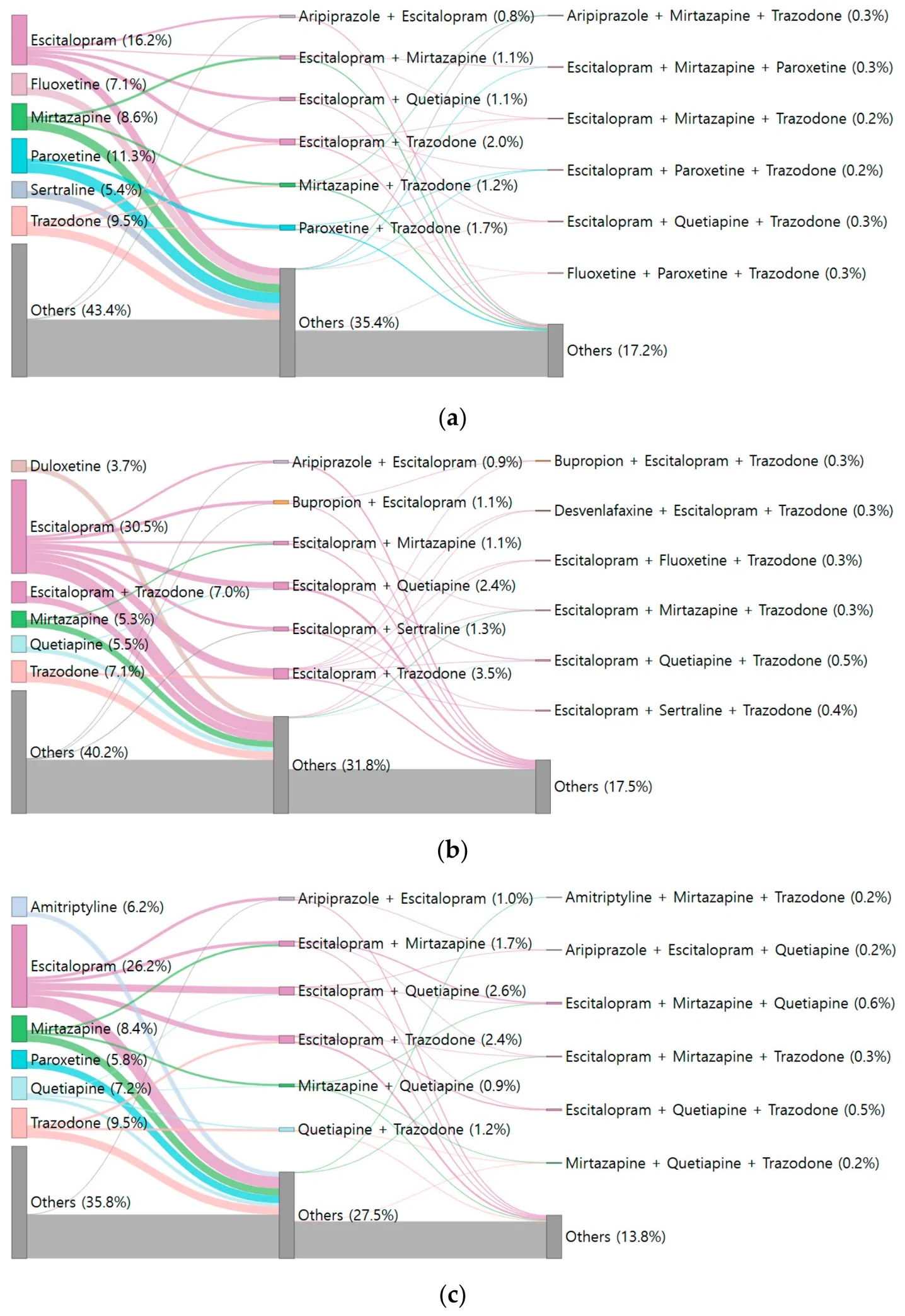

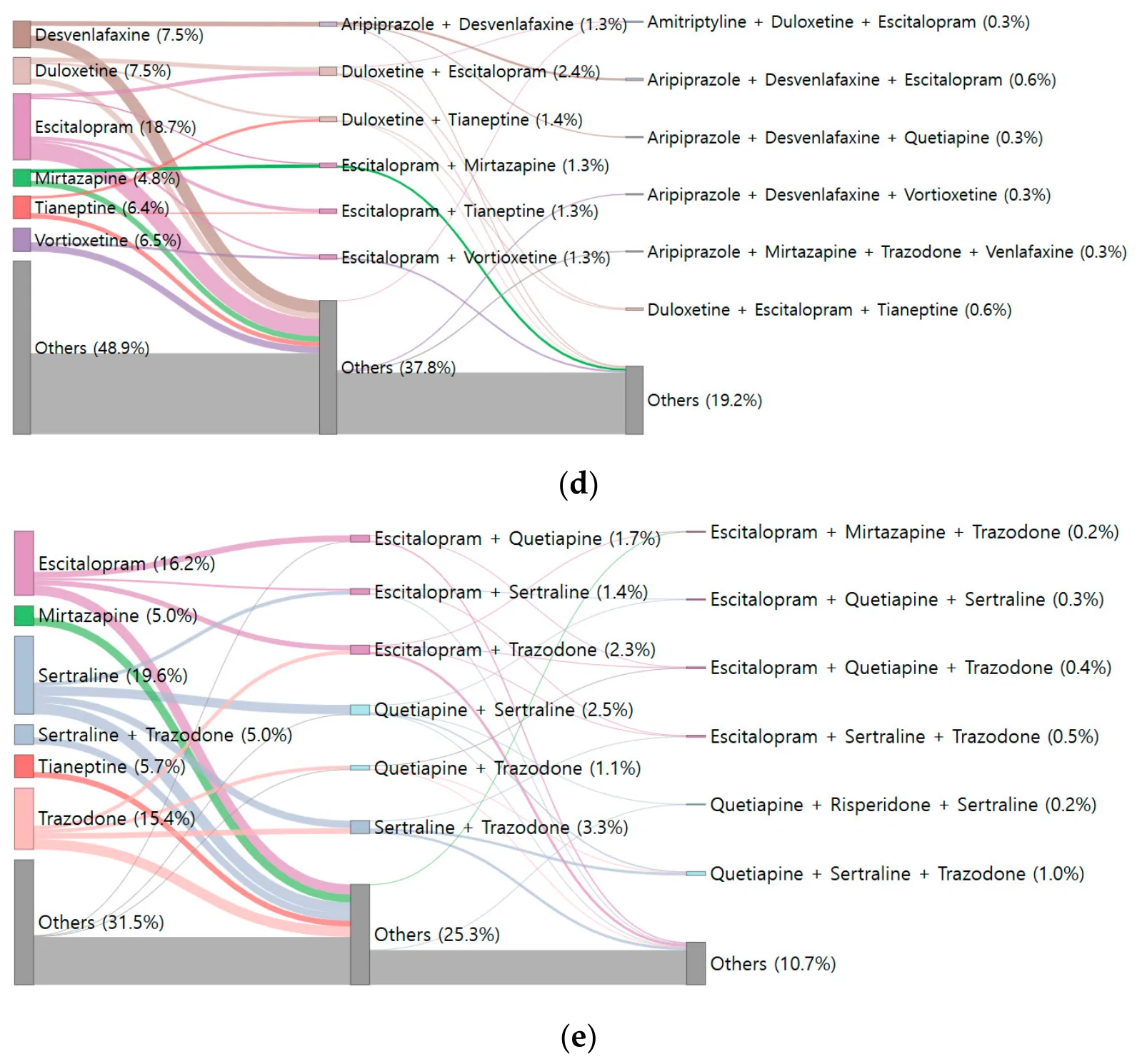
Sankey diagrams. At each step, the six most common regimens are displayed from STEP 1 to STEP 3 across five tertiary hospitals, and all remaining regimens are grouped as “Others.” Percentages represent the proportion of patients following each treatment pathway among all patients included in the treatment pathway analysis. The width of each flow is proportional to the corresponding percentage. (**a**) AUMC, (**b**) EUMC, (**c**) KDH, (**d**) KHNMC, and (**e**) KWMC. Abbreviations: AUMC, Ajou University Medical Center; EUMC, Ewha Womans University Medical Center; KDH, Kangdong Sacred Heart Hospital; KHNMC, Kyung Hee University Hospital at Gangdong; KWMC, Kangwon National University Hospital. Drugs appearing only in combination regimens (STEP 2 or 3) but not as initial monotherapy (STEP 1) are not listed as separate categories on the left axis. The color of all flow lines represents the initial treatment drug (Step 1), and is applied consistently throughout each treatment pathway.

The Sankey diagrams (Figure 2), which aggregate leading regimens at each step to visualize how patient volumes shift between treatment nodes across sequential stages, showed that subsequent treatment regimens varied across hospitals despite similar first-line antidepressants at treatment initiation. At STEP 2, treatment regimens including trazodone or quetiapine were commonly observed across several hospitals, but the specific regimen combinations varied based on institution. At EUMC, the most frequent STEP 2 regimens were escitalopram + trazodone (*n* = 87, 3.5%) and escitalopram + quetiapine (*n* = 60, 2.4%). Similar patterns were observed at KDH, where escitalopram + quetiapine (*n* = 84, 2.6%) and escitalopram + trazodone (*n* = 80, 2.5%) were commonly observed. At AUMC, escitalopram + trazodone (*n* = 52, 2.0%) and paroxetine + trazodone (*n* = 45, 1.7%) were also among the most common STEP 2 regimens. At KWMC, sertraline + trazodone (*n* = 146, 3.3%) and quetiapine + sertraline (*n* = 110, 2.5%) were relatively frequent. Conversely, at KHNMC, duloxetine + escitalopram (*n* = 15, 2.4%) and duloxetine + tianeptine (*n* = 9, 1.4%) were among the leading STEP 2 regimens.

At STEP 3, more complex treatment regimens were observed, although each regimen accounted for a relatively small proportion of the overall treatment pathway cohort. Common STEP 3 regimens included escitalopram + quetiapine + trazodone, escitalopram + sertraline + trazodone, and quetiapine + sertraline + trazodone, with the specific regimen patterns varying across hospitals. A considerable proportion of treatment pathways were grouped into the “Others” category at the overall pathway level, accounting for 0.3% (7/2676) at AUMC, 0.5% (13/2459) at EUMC, 0.6% (19/3234) at KDH, 0.6% (4/628) at KHNMC, and 1.0% (44/4384) at KWMC. This overall pathway-level “Others” category is distinct from the step-level “Others” category (Figure 2; approximately 17–44%, depending on the treatment step and hospital), which aggregates less common individual medications or treatment regimens at each step. This category comprises numerous frequently observed treatment regimens that could not be presented individually in Figure 2.

These findings indicate that, although medications (e.g., trazodone and quetiapine) often appear in subsequent treatment regimens, the dominant treatment pathways vary across hospitals. This suggests substantial institutional variability in real-world antidepressant treatment pathways beyond the initial regimen.

### 2.2. Meta-Analysis of Safety Outcomes

#### 2.2.1. Patient Characteristics

Overall, 44,542 patients with depressive disorder were included in the comparative safety analyses. The mean age was 54.2 ± 15.6 years, and 64.2% were female.

Baseline patient characteristics before and after propensity score matching (PSM), including a representative example (Table 1) and the entire set of outcome-specific covariate balance tables (Supplementary Table S1a–n), and corresponding Love plots (Supplementary Figure S3), demonstrated substantially improved covariate balance after matching, with most standardized mean differences (SMD) below the conventional threshold of 0.1. Across hospitals and outcomes, the comparator-to-target matching ratio and the corresponding effective sample size for each matched cohort are reported in Supplementary Tables S2–S4, and preference-score distributions before matching, which demonstrated adequate overlap between the treatment groups, are shown in Supplementary Figure S4. The proportion and identity of covariates retaining an absolute SMD greater than 0.1 after matching are summarized for every outcome and drug comparison in Supplementary Table S5.

**Figure 3:**
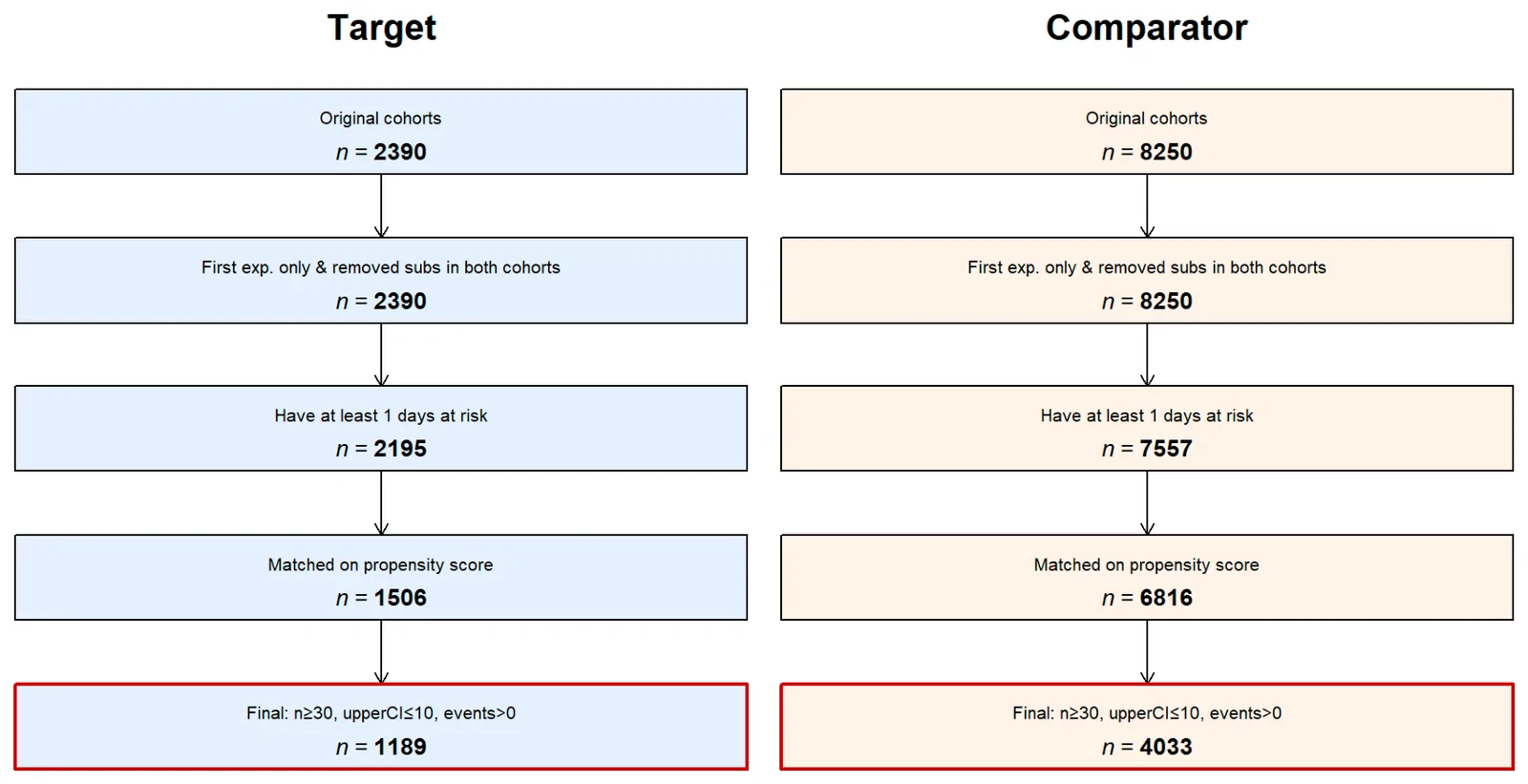
Flow diagram illustrating cohort construction for QTc prolongation (escitalopram vs. sertraline), a representative outcome. The diagram shows the number of patients and contributing hospitals at each stage: the source depressive disorder population, drug-specific new-user cohorts, application of exclusion criteria, propensity-score matching, and the final outcome-specific analytic cohort. Flow diagrams for all remaining outcome-by-drug-comparison combinations are provided in Supplementary Figures S7 and S8. Blue and orange boxes represent the Target and Comparator cohorts, respectively. The red-bordered box indicates the final analytic sample after applying inclusion criteria (*n* ≥ 30, upper confidence interval ≤ 10, and events > 0).

Figure 3 presents the flow diagram of patients and hospital selection for the comparative safety analysis, from the source depressive disorder cohort to the drug-specific new-user cohorts, propensity score-matched cohorts, and outcome-specific analytic cohorts. Since the number of eligible patients and contributing hospitals varied across hospital–outcome combinations based on outcome event counts and filtering criteria, the flow diagram for QTc prolongation (escitalopram vs. sertraline) is presented as a representative example. Supplementary Figures S5a–i and S6a–e show the flow diagrams for the remaining outcomes.

#### 2.2.2. Safety Comparison

In the 5-year time-at-risk (TAR) analysis, no significant differences were observed between sertraline and escitalopram across most evaluated safety outcomes. For example, risk estimates were comparable for common adverse events, including gastrointestinal system disorders (hazard ratio [HR] 1.26, 95% confidence interval [CI] 0.95–1.68) and cardiovascular outcomes, such as atrial fibrillation (HR 1.51, 95% CI 0.69–3.32). Other evaluated outcomes, including hematologic side effects, cardiac arrest, circulatory system, heart failure, hypersensitivity, and psycho-neurological system, also showed no significant differences between groups. QTc prolongation was the exception: after empirical calibration using 167 negative control outcomes to address non-negligible systematic bias, sertraline was associated with a statistically significant increase in risk relative to escitalopram (HR 1.75, 95% CI 1.10–2.78, *p* = 0.018; Supplementary Figure S7).

No statistically significant differences were observed between trazodone and escitalopram across any evaluated safety outcomes. Risk estimates for common adverse effects, including gastrointestinal system disorders (HR 1.29, 95% CI 0.93–1.79), were comparable between the two antidepressant groups, with all CIs including the null value (Supplementary Figure S8).

Matched sample sizes, event counts, mean follow-up duration, and incidence rates (per 1000 person-years) for every safety outcome, drug comparison, and contributing hospital are reported in Supplementary Table S6a,b.

Of these outcomes, gastrointestinal system disorder yielded subgroup estimates in both drug comparisons and is presented as the representative example (Supplementary Figure S9a–c). CIs included null values across all subgroups.

#### 2.2.3. Sensitivity and Subgroup Analyses

The robustness of the primary 5-year TAR findings was examined across several alternative analytic specifications. Across 1-year, 3-year, and 5-year TAR windows, pooled estimates were broadly consistent for most outcomes (Supplementary Figure S10). For the escitalopram-versus-sertraline QTc prolongation comparison, however, the calibrated estimate varied more noticeably across these windows: it was not significant using a 1-year TAR (HR 1.49, 95% CI 0.80–2.78), and was significant using both a 3-year TAR (HR 1.78, 95% CI 1.09–2.91) and the primary 5-year TAR (HR 1.75, 95% CI 1.10–2.78).

In an analysis removing the 60-day lag period entirely (0-day lag TAR), stable calibrated estimates were obtained for five safety outcomes that met the pre-specified hospital-level stability criteria—atrial fibrillation, gastrointestinal system, heart failure, hypersensitivity, and QTc prolongation—despite the reduced number of events available within this shorter specification; the remaining four outcomes did not meet the hospital-level stability criteria required for reliable estimation under this shorter window. None showed a statistically significant between-group difference; the QTc prolongation estimate in this specification was HR 1.01 (95% CI 0.55–1.86) (Supplementary Figure S9a).

Restricting the study population to a recent calendar period (index date between 1 January 2017 and 14 January 2026) yielded uncalibrated estimates consistent in direction with the primary analysis (Supplementary Figure S11); the escitalopram-versus-sertraline QTc prolongation estimate in this restricted period was HR 1.23 (95% CI 0.64–2.37). Within this period, the circulatory system, psycho-neurological system, and hematologic side effect categories were represented by the individual component conditions with sufficient events for stable estimation (tachycardia, bradycardia, and tremor).

Age- and sex-stratified analyses were conducted for outcomes with event counts sufficient to support stable estimation. Gastrointestinal system disorders, the outcome with the highest overall event rate, are presented as the representative example (Supplementary Figure S9b,c); the 95% CIs for both age (<65 vs. ≥65 years) and sex (male vs. female) subgroups included the null value in both drug comparisons.

A sensitivity analysis was performed excluding hospitals whose covariate balance was no longer available for independent re-verification, recalculating pooled calibrated HRs for both drug comparisons (Supplementary Figure S12). For most outcomes, estimates remained consistent with the primary analysis; notably, the escitalopram-versus-sertraline QTc prolongation estimate was materially unchanged (HR 1.78, 95% CI 1.08–2.94, based on four hospitals, versus the primary calibrated HR of 1.75, 95% CI 1.10–2.78), supporting the robustness of this finding to the exclusion of these hospitals.

## 3. Discussion

### 3.1. Principal Findings

In this multicenter real-world analysis, SSRIs—particularly escitalopram—were the most frequently prescribed initial antidepressants across most participating hospitals. These findings are generally consistent with current treatment guidelines, which recommend escitalopram as a first-line pharmacological option due to its favorable balance of efficacy, tolerability, and safety [1]. However, specific institutional differences were observed. Following escitalopram, sertraline, and trazodone were frequently selected as alternative initial treatments, depending on the hospital. Sertraline, widely used in routine clinical practice because of its efficacy and tolerability, is often preferred over escitalopram for patients with concerns about sexual dysfunction [3]. Similarly, the use of trazodone as an initial therapy suggests that clinicians may prioritize the immediate management of insomnia, severe sleep disturbance, or agitation at treatment initiation.

These prescribing-pattern findings are broadly consistent with those of a recent large-scale Danish nationwide register-based study of 113,175 individuals with unipolar depression followed for 5 years. The study shows that SSRIs are the predominant first-line antidepressant class before (55.4%) and after (47.7%) hospital diagnosis, and 60.8%, 33.7%, and 17.1% of patients proceed to a second, third, and fourth treatment trial, respectively. Similarly, the study reports substantial deviation from guideline recommendations and considerable heterogeneity in treatment patterns [11]. Within the Korean context, a nationwide claims-based analysis of antidepressant prescribing from 2014 to 2023 similarly reports a steady increase in SSRI prescribing alongside a continuous decline in TCA use [12], consistent with the SSRI-predominant pattern observed in our cohort. A recent multicenter Korean OMOP-CDM study involving 15 hospitals reports that SSRIs, rather than SNRIs or TCAs, are associated with a significantly increased risk of hyponatremia compared with other antidepressant classes, particularly among patients aged ≥ 60 years [13]. In the present study, no clear differences were detected across escitalopram, sertraline, and trazodone for most of the nine safety outcomes evaluated, suggesting that antidepressant safety differences may be outcome-specific rather than uniform across adverse event types.

Beyond initial treatment selection, treatment modifications more often involved adjunctive therapy rather than switching antidepressants. Consistent with the sunburst plot, patients generally continued their initial antidepressant or received adjunctive medications during subsequent treatment stages. Trazodone, quetiapine, and mirtazapine were the most commonly prescribed adjunctive medications across several hospitals. Given their sedative properties, the frequent use of these agents in subsequent treatment pathways likely reflects efforts to manage residual symptoms, including insomnia, anxiety, agitation, and partial treatment response, rather than depressive mood symptoms alone [10,14,15,16]. Similarly, studies report the frequent use of sedative antidepressants and atypical antipsychotics as adjunctive agents in patients with an incomplete response or persistent sleep-related symptoms during antidepressant treatment [17]. This pattern indicates that real-world antidepressant treatment extends beyond medication replacement for depressive mood symptoms alone and instead reflects individualized, symptom-oriented management.

Furthermore, this visualization approach provided deeper insights into the complexity and heterogeneity of prescribing patterns. The sunburst plots identified less commonly used agents at treatment initiation, including paroxetine, duloxetine, and vortioxetine. Although trazodone, quetiapine, and mirtazapine were commonly used adjunctive medications, their utilization varied considerably based on institution and index antidepressant, and no single combination strategy consistently predominated across hospitals. Additionally, a substantial proportion of treatment pathways were categorized as “Others” in the Sankey analysis, particularly at later treatment stages, indicating that real-world antidepressant treatment involves numerous less frequent medication combinations that cannot be fully captured by the most common treatment sequences alone [18]. Collectively, these findings suggest that antidepressant selection and subsequent modifications are influenced by guideline recommendations, patient characteristics, symptoms, and institutional prescribing preferences. Because clinician intent, symptom-level basis for treatment changes, and off-label prescribing motivations were not directly recorded in the underlying EHR/CDM data, these interpretations remain hypotheses regarding prescribing behavior rather than confirmed clinical indications.

The study period spanned nearly three decades (1999–2026). During this period, antidepressant availability, diagnostic coding practices, treatment guidelines, and EHR systems changed substantially. Therefore, the observed prescribing and safety patterns should be interpreted within this evolving clinical context. The third revision of the Korean Medication Algorithm for Depressive Disorder (KMAP-DD), published in 2017, designated escitalopram and sertraline as first-line treatments for mild-to-moderate depressive episodes and escitalopram as the sole first-line treatment for severe and psychotic depression, a designation not present in the prior 2012 revision [19]. Consistent with this guideline change, period-stratified prescribing patterns at two hospitals with sufficient case volumes differed substantially before and after 2017 (Section 2.1.2), indicating that evolving guideline recommendations can substantially influence prescribing behavior and that pooling across the entire study period may obscure period-specific trends.

For comparative safety, no clear differences were detected for most evaluated outcomes within the limitations of the available data, with one exception: an increased QTc-prolongation risk was observed with sertraline relative to escitalopram (HR 1.75, 95% CI 1.10–2.78), which is discussed separately below. Because no formal equivalence or noninferiority framework was applied, these findings indicate that no significant differences were detected in this sample rather than that the agents are equivalently safe; several point estimates, including atrial fibrillation (HR 1.51, 95% CI 0.69–3.32), retained confidence intervals compatible with a clinically meaningful increase in risk. The same applies to the trazodone comparisons, which should not be read as evidence that trazodone and escitalopram are clinically interchangeable, particularly in view of the residual confounding by indication discussed in Section 3.2.

An additional finding warranting specific comment was the escitalopram-versus-sertraline comparison for QTc prolongation, for which the raw HR (HR 1.56, 95% CI 0.98–2.48) did not reach statistical significance but the empirically calibrated estimate did (HR 1.75, 95% CI 1.10–2.78). This shift arose because the estimable negative controls exhibited effect estimates that systematically deviated from the null (HR = 1) in a protective direction, with relatively small variance across hospitals; empirical calibration removed this systematic underestimation from the point estimate while only modestly widening the confidence interval, shifting its lower bound from just below (0.98) to above (1.10) unity. By contrast, for outcomes such as the circulatory system and atrial fibrillation, the estimated bias from negative controls was minimal or in the opposite direction, leaving qualitative conclusions unchanged before and after calibration. Rather than treating this as confirmed evidence of a pharmacological effect of sertraline on cardiac repolarization, we examined it further across the alternative analytic specifications reported in Section 2.2.3. The estimate varied with the length of the TAR window and was attenuated when the 60-day lag was removed or when follow-up was restricted to a recent calendar period. This specification-dependent pattern argues for cautious interpretation, rather than either dismissing the signal outright or prematurely attributing it to a confirmed drug effect.

Existing evidence on sertraline and QTc prolongation is itself mixed: sertraline is generally considered to carry a comparatively low risk of clinically significant QTc prolongation among SSRIs at therapeutic doses, whereas some comparative analyses have suggested escitalopram carries a higher intrinsic potential for dose-dependent QTc prolongation [20]; On the other hand, a thorough QTc study demonstrated a clear dose-dependent QTc-prolonging effect of sertraline at supratherapeutic exposures, providing biological plausibility for an exposure-related risk that may not be fully captured within the therapeutic dose range typically evaluated in short-term trials [21]. Furthermore, it should be noted that QTc prolongation and torsade de pointes are not synonymous, and QTc prolongation alone, in the absence of other established risk factors, is an imperfect predictor of clinically meaningful arrhythmic events [22]. Whether this finding reflects a true, exposure-dependent safety signal, residual confounding, or chance cannot be resolved within the present observational design; it is therefore presented as a hypothesis-generating signal that warrants targeted external confirmation. Differential ECG surveillance and channeling bias represent additional plausible explanations for this finding. Specifically, because escitalopram carries established regulatory cautions regarding QTc prolongation, clinicians may be more inclined to obtain baseline or follow-up ECGs in escitalopram users, or conversely, may preferentially avoid prescribing escitalopram to patients with pre-existing cardiac risk factors. Either pattern of differential diagnostic testing or selective prescribing could contribute to the observed between-drug difference, independent of a true pharmacological effect.

Collectively, these findings highlight the complexity of real-world antidepressant prescribing patterns and emphasize the importance of evaluating treatment strategies at the individual drug level rather than the therapeutic class level [18]. These findings may provide clinicians with practical evidence to better understand routine prescribing patterns and optimize real-world antidepressant treatment strategies.

### 3.2. Limitations

Comparisons involving trazodone may still be subject to residual confounding by indication because trazodone may be prescribed as a primary antidepressant or, particularly at lower doses, to manage insomnia or sleep disturbances. However, excluding patients with a sleep disorder diagnosis at any point during the observation period from the cohort (Section 4.2.1) substantially reduced this potential source of confounding because no included patient had a recorded sleep disorder diagnosis that could serve as a proxy indication for trazodone use. Dose was not incorporated as a covariate because free-text dose annotations (e.g., “100 mg”) could not be reliably distinguished from fractional doses of higher-strength tablets (e.g., half of a 100 mg tablet). Including these data would have introduced additional measurement error. Concomitant sedative and antipsychotic medications were included as baseline covariates and balanced using PSM. However, these residual sources of confounding by indication should be considered when interpreting the trazodone-related safety comparisons.

As with all observational studies, unmeasured confounders related to the clinical care setting—including physician preferences, patient adherence, and unmeasured clinical characteristics—could not be fully accounted for [6,7,8]. Neurological and cognitive comorbidities (e.g., cerebral infarction, dementia, and traumatic brain injury), the comorbidity domain most mechanistically related to several evaluated safety outcomes, were included as baseline covariates and balanced using PSM. Sensitivity analyses excluding or stratifying by other comorbidities, such as borderline personality disorder, obsessive-compulsive disorder, active malignancy, and fibromyalgia, were not performed, as these conditions are not directly associated with the pathophysiology of the cardiovascular, gastrointestinal, or hypersensitivity outcomes evaluated, and such comprehensive stratification exceeded the scope of the distributed multicenter data-access framework.

Several limitations relate to the underlying data source. Standardized severity classifications were not consistently available across participating databases, so differences in treatment patterns by clinical severity could not be evaluated; given that the cohort comprised patients with newly diagnosed depressive disorder, however, it likely consisted predominantly of individuals with mild to moderate disease severity. Treatment pathways were defined using prescription records, which may not fully reflect the actual duration of medication use, and adherence to prescribed regimens could not be confirmed. Because discontinuation of the index medication alone was not treated as a censoring event under our modified intention-to-treat (ITT) framework, exposure misclassification during long-term follow-up cannot be excluded; this is an inherent limitation of observational studies using claims-based or EHR-derived database networks, in which actual medication-taking behavior cannot be directly verified. Prescribing specialty, depressive-disorder-related healthcare utilization (e.g., psychotherapy), and educational attainment were not captured within this OMOP-CDM infrastructure and could therefore not be incorporated as additional covariates or indication proxies. Several analytic constraints also warrant mention. Because the automated multicenter platform returns only final aggregated Cox model outputs, formal residual-based diagnostics for the proportional-hazards assumption could not be performed; comparison of HRs across 1-year, 3-year, and 5-year TAR windows was therefore used as an indirect assessment (Section 2.2.3).

Nine outcomes were evaluated across two drug comparisons without a pre-specified primary endpoint or correction for multiple testing, and the single statistically significant finding should be interpreted in this multiplicity context; applying a Bonferroni correction for the eighteen comparisons performed (α = 0.05/18 ≈ 0.0028) would render it no longer statistically significant (calibrated *p* = 0.0175). In addition, because the cohort-overlap structure described in Section 4.3.1, the outcome-specific findings within each comparison are not fully statistically independent of one another; each Cox model and meta-analysis was nonetheless estimated separately, so this overlap does not bias any single reported effect estimate.

Finally, because the data were obtained from tertiary hospitals, the observed prescribing patterns and safety estimates may not be fully generalizable to primary or secondary care settings. Patients treated at tertiary hospitals may have more complex clinical presentations, a higher burden of psychiatric or medical comorbidities, or referral after an inadequate response to treatment in primary care, and prescribing decisions at these institutions may be more specialist-driven than those in general practice. Confirming condition-specific associations more directly would require individual-level EMR review or prospective study designs, which we suggest as directions for future research.

## 4. Materials and Methods

### 4.1. Study Design and Data Source

In this retrospective, multicenter observational study, EHR data standardized to the OMOP-CDM in South Korea were used. Data extraction, using a uniform final cutoff date of 14 January 2026, served as an initial platform feasibility assessment. Subsequent cohort construction, statistical analyses, and reporting were conducted in treatment pathway analysis and comparative safety analysis, respectively. The study protocol was reviewed and granted exemption prior to study initiation by the IRB at Ewha Womans University Medical Center (EUMC), which also waived the requirement for informed consent owing to the retrospective study design and use of de-identified data (20 April 2026).

### 4.2. Treatment Pathway Analysis

#### 4.2.1. Cohort Definition and Operational Criteria

The target cohort comprised patients with an index date defined as the first diagnosis of a depressive disorder, identified using three SNOMED CT concepts—depressive disorder (35489007), mild depression (310495003), and mild major depression (87512008)—together with all descendant concepts, followed by ≥1 antidepressant prescription after diagnosis across five tertiary university hospitals: AUMC, EUMC, KDH, KHNMC and KWMC. Patients were required to have ≥365 days of continuous observation before the index date. Patients were excluded if they had a diagnosis of bipolar disorder, schizophrenia, anxiety disorders, pregnancy, or sleep disorders at any time during the study period (concept identifiers listed in Supplementary Table S7).

The event cohorts were defined for each specific antidepressant and related psychotropic ingredient rather than at the class level. Individual agents were categorized based on pharmacologic class for descriptive analysis: SSRIs (escitalopram, fluoxetine, fluvoxamine, paroxetine, and sertraline); SNRIs (desvenlafaxine, duloxetine, milnacipran, and venlafaxine); TCAs (amitriptyline, imipramine, and nortriptyline); other antidepressants (agomelatine, bupropion, mirtazapine, tianeptine, trazodone, and vortioxetine); atypical antipsychotics (aripiprazole, olanzapine, quetiapine, and risperidone); and the mood stabilizer (lithium).

Within the treatment pathway analysis, specific operational criteria were applied to define prescribing patterns and treatment transitions: Initial regimen was defined as the first antidepressant agent prescribed on or after the initial diagnosis date of depressive disorder, with initial combination therapy requiring same-day initiation of multiple agents. In the pathway analysis, drug event cohorts were defined such that an event persisted from its initiation date until the end of continuous observation. Cohort eras were constructed with a 0-day gap window (no collapse gap). Because the exposure end was determined by the onset of a subsequent step event or observation termination, gap and stockpiling rules were not applied at the pathway transition level. A transition involving a complete replacement of the drug agent was operationalized as switching, whereas a transition where the index agent was maintained and a new agent was added was operationalized as augmentation (add-on). Discontinuation was considered to occur when the index drug was replaced or augmented; otherwise, discontinuation was not explicitly tracked at the individual drug cohort level. Temporally and clinically, a transition from Step 1 to Step 2 represents the initiation date of a new drug event cohort (a distinct single agent or combination regimen) following the index regimen.

To further characterize prescribing patterns, stratified pathway analyses were conducted using age (<65 vs. ≥65 years), sex (male vs. female), and calendar period (before vs. after 2017) at two representative hospitals with distinct first-line prescribing patterns (KDH, escitalopram-predominant; KWMC, sertraline-predominant).

#### 4.2.2. Sunburst and Sankey Visualization Analysis

Treatment sequences were visualized using sunburst plots generated via ATLAS (version 2.12.0) to illustrate overall nested hierarchical treatment pathways from STEP 1 to 3. A minimum cell count of five patients was required for an event to be included in the treatment pathway visualization. The maximum pathway length was set to three steps, and repeated cohort events within the same pathway were not permitted.

While sunburst plots effectively display branching treatment trajectories initiated from specific first-line agents, their radial structure becomes fragmented at later steps, making it difficult to trace aggregate step-to-step transitional shifts. To overcome this limitation and capture node-to-node patient movements, Sankey diagrams were generated separately for each hospital using the networkD3 package (version 0.4.1) in R (version 4.5.1) to delineate the dominant treatment flows and directional transition dynamics across STEP 1–3.

STEP 1 represented the initial antidepressant regimen, whereas STEP 2 and 3 represented subsequent treatment regimens. At each step, the six most frequently observed treatment regimens were retained, and remaining regimens were grouped into an “Others” category. The width of each flow was proportional to the percentage of patients following the corresponding pathway among all patients included in the treatment pathway cohort at each hospital.

### 4.3. Comparative Safety Analysis

#### 4.3.1. Study Population

The safety analysis cohort was derived from the same base population as the target cohort of the treatment pathway analysis described in Section 4.1 and Section 4.2 and was therefore subject to the same diagnostic exclusion criteria (bipolar disorder, schizophrenia, anxiety disorders, pregnancy, and sleep disorders). Eligibility criteria were applied across an expanded network of 18 tertiary and specialized hospitals (Supplementary Tables S1 and S8). Patients with prior exposure to any of the three index drugs (escitalopram, sertraline, or trazodone) before the index date were excluded. Patients with a history of the outcomes of interest were also excluded to avoid misclassification of incident events. Two separate target cohorts were established, comprising first-time users of sertraline and trazodone, respectively. Both cohorts were compared to a single comparator cohort of first-time users of escitalopram. Patients with prior exposure to the comparator drug before the index date were excluded. Because the escitalopram comparator cohort was shared across both drug-comparison pairs, an individual escitalopram new-user could appear as the comparator in both the escitalopram-versus-sertraline and escitalopram-versus-trazodone analyses; sertraline and trazodone new-users, in contrast, contributed to only one comparison pair each. Within a given drug comparison, an individual patient could also contribute to multiple outcome-specific analyses, provided they met the specific cohort-eligibility and observation-window criteria for each outcome.

Baseline demographic and clinical characteristics, including age, sex, comorbidities, and concomitant medications, were assessed before the index date (overall sex and age distribution of the safety cohort by hospital is presented in Supplementary Table S9). For baseline comorbidities and clinical characteristics, the absence of a recorded diagnosis or medication code was considered as the absence of the corresponding condition. Standardized measures of depressive disorder severity were not consistently available across the EHR-based OMOP-CDM data sources used in this study; therefore, disease severity could not be evaluated as a covariate or stratification variable.

Matching adequacy was further assessed using the target-to-comparator matching ratio and effective sample size for each hospital and outcome, calculated as 4 × (Target N × Comparator N)/(Target N + Comparator N) [23]; Love plots summarizing covariate balance before and after matching across all analyses; and preference-score distributions before matching, given that patient-level propensity scores after matching are not exported from each site under this distributed-network’s data-governance structure. The proportion and identity of covariates retaining an absolute SMD greater than 0.1 after matching are reported for each outcome and drug comparison, rather than inferred from Table 1 alone. This upper-bound property of masked proportions does not extend to SMDs, because an SMD reflects the difference between two group proportions rather than a single proportion; substituting an upper-bound value into one group’s proportion does not guarantee that the resulting SMD is itself an upper bound. SMDs derived from covariate cells involving one or more masked counts are therefore treated as approximations. A sensitivity analysis comparing SMDs calculated under a lower-bound (count = 0) versus upper-bound (count = 4) assumption for masked cells showed a difference not exceeding 4.0 percentage points across all safety outcomes and both drug comparisons (Supplementary Table S10). Also, because masking affects only the reporting of aggregate counts and not the underlying patient-level matching, the resulting SMD was identical between the two assumptions.

#### 4.3.2. Population-Based Estimation of Safety Outcomes and Exposure Censoring Rules

Safety outcomes were classified into nine categories: atrial fibrillation, hematologic events, cardiac arrest, circulatory system, gastrointestinal system, heart failure, hypersensitivity reactions, psycho-neurological system, and QTc prolongation. Full concept set definition (SNOMED CT concept identifiers) and phenotype algorithms for each category are provided in Supplementary Table S11, and concept identifiers for all study medications are listed in Supplementary Table S12.

Four of the nine categories were composite phenotypes constructed by grouping related SNOMED CT concepts within a given physiological system. All concept sets were defined using the include-descendants option in ATLAS, so that every child concept subordinate to a listed top-level concept was captured even where only that top-level code appears in the concept tables. Incident events were identified from standardized OMOP-CDM condition occurrence records; as is characteristic of observational studies conducted on standardized common data models, formal chart-review validation of these outcome phenotypes was not undertaken.

In two supplementary analyses, individual component conditions were evaluated separately where feasible: an age- and sex-stratified subgroup analysis of three clinically salient conditions (atrial fibrillation, gastrointestinal system, sexual dysfunction; Supplementary Figure S9b,c), and, within the sensitivity analysis restricted to 2017–2026 (Supplementary Figure S11a,b), disaggregation of the categories of circulatory system, psycho-neurological system, and hematologic side effects into individual component conditions. Because the smaller sample size within this restricted period yielded fewer than five negative controls with finite effect estimates for any outcome, empirical calibration could not be reliably performed; HRs for this sensitivity analysis are therefore reported as uncalibrated estimates.

The primary TAR was defined as the period starting after a 60-day lag period after the index date until the occurrence of an event or censoring. The 60-day lag period was applied to minimize protopathic bias, arising from pre-existing symptoms present shortly after the index date that prompted the initial prescription. As a sensitivity analysis, the analysis was repeated using a 0-day lag (TAR beginning on the index date) to evaluate early safety signals (Supplementary Figure S9a). For patients with multiple occurrences of the same adverse events, only the first event was included as the primary endpoint to maintain statistical independence and avoid overestimating the risk associated with recurrent events.

To rigorously define treatment exposure and follow-up, a modified ITT framework was employed. Patients were followed continuously from the index date until the earliest occurrence of any of the following six censoring events: Initiation of the comparator drug (treatment switching), addition or augmentation with another antidepressant, loss of continuous observation (loss to follow-up), death, first occurrence of the specific study safety outcome, or administrative end of the 5-year follow-up period. Discontinuation of the index medication itself without switching or augmentation was not treated as a censoring event. Consequently, follow-up continued up to 5 years after index initiation unless one of the six censoring criteria was met.

Comparative safety analyses were performed using Cox proportional hazards models in propensity score–matched cohorts at each hospital. Propensity scores were estimated from baseline demographic and clinical characteristics. Matched cohorts were constructed using 1:N nearest-neighbor PSM without replacement, with a caliper width of 0.2 of the standard deviation of the logit of the propensity score. No upper limit was imposed on the matching ratio.

All analyses were conducted using the OMOP Common Data Model (version 5.3.1), ATLAS (version 2.12.0), and the CohortMethod R package (https://github.com/SandyRhie/depression, accessed on 18 June 2026). To prevent variance inflation and ensure statistical reliability, strict institutional filtering rules were applied: individual hospitals were included in the pooled outcome analysis only if they had ≥30 matched patients and an upper confidence limit ≤ 10 for that specific outcome, a stability criterion pre-specified prior to analysis. This threshold reflects the well-documented risk that standard normal-approximation-based meta-analytic methods can yield biased or unstable estimates when hospital-level event counts are small, particularly across heterogeneous distributed research networks [24]. We acknowledge, however, that exclusion-based approaches such as ours carry their own risk of selection bias, and that non-normal likelihood approximation methods offer a principled alternative for future refinement of this analysis. A complete list of excluded hospitals and a sensitivity analysis repeating the meta-analyses without this exclusion criterion are provided in Supplementary Table S13 and Supplementary Figure S13, respectively. Because outcome event rates varied across sites, the number of contributing hospitals differed by outcome, with certain rare outcomes supported by fewer hospitals. Outcomes with no hospitals meeting these stability criteria were excluded from the final synthesis.

Since this was conducted at a multicenter federated distributed network, all primary HR estimates and baseline covariate balance metrics were fully generated and verified during the active execution phase. Subsequent institutional data governance updates and enhanced local security protocols at a small subset of hospitals restricted post-hoc raw file re-extraction, as noted in Supplementary Table S1.

For a small subset of hospitals, the underlying results are no longer available for independent re-verification, so we are unable to confirm whether the original covariate-balance criteria were reconfirmed prior to inclusion for these sites. The aggregated effect estimates for these hospitals were retained within the central analytic platform. To address this, we additionally performed a sensitivity analysis excluding all hospitals for which complete results were unavailable; the recalculated pooled estimates, presented in Supplementary Figure S12, were consistent with the primary analysis.

#### 4.3.3. Meta-Analysis and Empirical Calibration

Effect estimates from the propensity score-matched analyses at each hospital were pooled using meta-analysis. All data management, statistical analyses, and visualizations were performed in Python (version 3.12.12) within the Google Colaboratory environment. Forest plots were generated using the matplotlib library. All statistical tests were two-sided, and a *p*-value < 0.05 was considered statistically significant.

Hospital-specific log-transformed HRs and corresponding 95% CIs were pooled on the natural log scale using a random-effects meta-analysis with the DerSimonian–Laird estimator and standard inverse-variance weighting, when valid estimates were available from at least two hospitals. CIs were calculated using the standard normal distribution, and this approach accounted for differences in patient populations and prescribing practices while preserving between-hospital variability. Between-hospital heterogeneity was assessed using the I^2^ statistic and the between-hospital variance (τ^2^) from the random-effects model, both reported for each outcome in Supplementary Figures S7 and S8. Meta-analyses were performed separately for each clinical outcome and drug comparison. Sensitivity analyses using an alternative 3-year TAR were conducted to assess the robustness of the primary 5-year TAR findings. Because the standardized OMOP-CDM/ATLAS platform outputs only final aggregated Cox model estimates, residual-based diagnostics for the proportional-hazards assumption (e.g., Schoenfeld residuals) were not accessible. As an indirect check, HRs were additionally compared across 1-year, 3-year, and 5-year TAR windows for every safety outcome and drug comparison (Supplementary Figure S10a–d); meaningful variation across these windows may indicate that the HR is not constant over the full follow-up period.

To assess and adjust for residual systematic error and unmeasured confounding not addressed by PSM, empirical calibration was performed using 167 prespecified negative control outcomes (SNOMED concept sets covering musculoskeletal, infectious, dermatologic, gynecologic, and urologic conditions; listed in Supplementary Table S14). A systematic error distribution was fitted to the negative control effect estimates to drive calibrated HRs and 95% CIs for each safety outcome. Negative control outcomes were selected a priori based on concepts established in the observational research literature [25,26]. Each negative control was evaluated separately at the individual-hospital level for every outcome and drug comparison. The systematic error distribution was estimated using the fitNull() function of the EmpiricalCalibration R package, which assumes that the distribution of log HRs among true negative controls is normal, yielding a null mean and null standard deviation; calibration was applied once to the pooled hospital-level estimate, rather than separately at each hospital. The number of negative controls with estimable results for each outcome and drug comparison is reported in Supplementary Table S15, and uncalibrated and calibrated HRs, 95% CIs, and *p* values for all outcomes are reported in Supplementary Table S16. Calibrated HRs are reported as the primary results throughout the manuscript. Calibration plots are presented in Supplementary Figure S14a,b. All nine safety outcome evaluations across both drug comparisons were conducted as exploratory analyses without a pre-specified primary endpoint hierarchy or adjustment for multiple comparisons.

Hospital-pooled HRs for safety outcomes were additionally estimated within age (<65 vs. ≥65 years) and sex (male vs. female) subgroups, and within a subset restricted to a recent calendar period (index date between 1 January 2017 and 14 January 2026), for both drug comparisons. Because sufficient event counts for stable subgroup estimation could only reasonably be expected for outcomes with a higher overall event rate, detailed age- and sex-stratified analyses were conducted for a subset of outcomes with adequate event numbers, such as gastrointestinal system disorders.

## 5. Conclusions

This study shows that escitalopram, sertraline, and trazodone are commonly prescribed in routine clinical practice, while subsequent treatment pathways vary considerably across institutions. No clear differences in comparative safety were detected for most outcomes evaluated, within the limitations of the available data; this should not be interpreted as evidence that the three agents are equivalently safe, particularly given the rarity of several events and the width of the corresponding confidence intervals. A notable exception was an increased risk of QTc prolongation with sertraline relative to escitalopram, which requires external confirmation before being interpreted as a confirmed drug effect. Given the low incidence of events and the residual confounding by indication affecting the trazodone comparisons in particular, these findings warrant further long-term prospective validation rather than immediate clinical reassurance; integrating treatment pathways and comparative safety analyses nonetheless emphasizes the importance of individualized, evidence-based treatment decisions.

## Figures and Tables

**Table 1:** Baseline Characteristics Before and After Propensity Score Matching. Representative baseline characteristics for the escitalopram-versus-sertraline comparison of QTc prolongation, before and after PSM; the complete sets for all nine outcomes and both comparisons are in Supplementary Table S1a–n.

	Before PSM Adjustment			After PSM Adjustment		
Covariate	Escitalopram (*n* = 1674)	Sertraline (*n* = 3414)	Std. Diff	Escitalopram (*n* = 1189)	Sertraline (*n* = 4033)	Std. Diff
Age and Sex						
18–19	3.4	1.8	0.173	3.8	3.9	0.058
20–24	6.2	4.9	0.092	6.4	7.2	0.002
25–29	3.9	2.8	0.096	≤3.3	3.5	0.022
30–34	3.5	2.8	0.041	≤3.0	3.3	0
35–39	4.5	3.5	0.095	4.8	4.1	0.078
40–44	4.3	4.3	0.017	≤4.7	5.8	−0.055
45–49	5.6	5.1	0.042	5.9	6.1	0.006
50–54	7.4	6.2	0.011	≤6.9	7.6	−0.076
55–59	6.1	7.5	0.002	6.6	8.3	−0.005
60–64	8.1	8.7	−0.006	8.3	8.2	0.017
65–69	8.8	8.7	0.007	9.3	8.9	0.013
70–74	9.2	10.8	−0.068	9.6	9.9	−0.011
75–79	≤9.3	13.9	−0.182	≤9.0	9.8	−0.05
80–84	≤10.4	11.5	−0.153	≤10.1	8.0	−0.011
85–89	5.8	6.4	−0.058	5.0	4.1	0.06
90–94	≤2.0	2.1	−0.07	≤1.8	1.3	−0.008
95–99	≤0.5	≤0.5	−0.02	≤0.9	≤0.4	0.008
Female	60.1	64.6	−0.107	61.9	62.4	−0.022
Comorbidities						
Mild depression	≤47.1	28.6	−0.108	≤35.2	23.5	−0.034
Recurrent depressive disorder	≤8.4	8.4	−0.055	≤9.6	8.6	−0.001
Moderate depression	20.8	16.6	0.03	26.2	19.0	0.008
Inflammation of specific body systems	29.6	37.1	−0.071	32.4	33.0	0.056
Degenerative disease of the central nervous system	≤16.4	24.3	−0.364	≤15.5	12.0	−0.004
Cognitive disorder	≤9.8	9.0	−0.178	≤9.1	6.9	−0.058
Cerebral degeneration	≤16.0	24.1	−0.369	≤15.2	11.9	−0.008
Traumatic and/or non-traumatic brain injury	≤1.4	0.8	0.005	≤1.4	≤0.6	0.045
Hyperlipidemia	13.3	20.5	−0.205	14.6	13.8	0.012
Alzheimer’s dementia	≤15.1	22.6	−0.352	≤14.0	10.9	−0.005
Cerebral infarction	6.5	12.4	−0.164	7.4	8.5	−0.033
Heart disease	≤9.2	12.3	−0.106	≤10.5	8.7	0.037
Concomitant Medications						
Other ophthalmologicals	37.8	40.6	−0.086	39.9	37.6	0.006
Proton pump inhibitors	14.6	19.4	−0.114	16.4	17.8	−0.035
ACE inhibitors	1.4	2.1	0.032	≤1.8	1.8	0.028
Drugs for constipation	≤15.8	17.3	−0.108	≤16.3	17.2	−0.102
Vitamin B1, plain	13.1	17.8	−0.116	14.2	15.0	−0.037
Beta-blocking agents	17.4	16.0	0.046	18.7	16.3	0.057
Antipruritics, incl. antihistamines, anesthetics, etc.	13.8	13.9	0.002	15.2	14.0	0.04
Acetaminophen	21.3	19.8	−0.023	20.6	19.5	0.005
Antiepileptics	18.7	18.9	0.01	17.6	18.6	0.022
Other plain vitamin preparations	12.0	17.5	−0.1	13.5	14.3	−0.004
Antianemic preparations	12.1	12.4	−0.085	≤12.8	11.4	−0.036
Statins (HMG CoA reductase inhibitors)	14.6	23.0	−0.253	16.1	14.4	0.014
Angiotensin II antagonists	11.0	16.9	−0.191	12.7	10.7	0.039
Atypical antipsychotics	5.8	4.1	0.184	6.9	7.3	0.057
Amlodipine	≤6.8	11.4	−0.185	≤8.3	7.2	−0.003
Aspirin	≤12.3	15.3	−0.189	≤11.9	10.6	0.007
Metformin	≤7.0	8.7	−0.107	≤7.7	6.6	0.028

For covariates with <5 patients at a participating hospital, the exact count was replaced with a fixed negative sentinel value to comply with the minimum cell-size policy for privacy protection. For these covariates, the upper masking threshold of four patients was used to calculate the upper-bound proportion. Pooled estimates that included data from ≥1 masked hospitals are reported as “≤” to indicate an upper-bound estimate rather than an exact observed value. Included hospitals were DCMC, EUMC, KWMC, and MJH. Three additional hospitals (CAUHS, GNUH, and SEJONG_ICN) contributed to the analysis but were excluded from this table because the results became inaccessible. Abbreviations: CAUHS, Chung-Ang University Hospital; DCMC, Daegu Catholic University Medical Center; EUMC, Ewha Womans University Medical Center; GNUH, Gyeongsang National University Hospital; KWMC, Kangwon National University Hospital; MJH, Myongji Hospital; SEJONG_ICN, Incheon Sejong Hospital.

## Data Availability

Raw, patient-level data and certain hospital-level diagnostic and execution log files are not publicly available because of institutional data-governance policies and platform access restrictions at participating hospitals. Further inquiries regarding data access can be directed to the corresponding author.

## Supplementary Materials

The following supporting information can be download at: https://www.mdpi.com/article/10.3390/ph19091332/s1. Supplementary Table S1(a–n): Baseline Characteristics of Patients Included in the Safety Analysis; Supplementary Table S2: Hospital-Level Inclusion and Exclusion by Outcome (Escitalopram vs. Sertraline); Supplementary Table S3: Hospital-Level Inclusion and Exclusion by Outcome (Escitalopram vs. Trazodone); Supplementary Table S4: Pooled Target-to-Comparator Matching Ratio by Safety Outcome; Supplementary Table S5: Proportion and Identity of Covariates with an Absolute Standardized Mean Difference Greater Than 0.1 After Propensity Score Matching; Supplementary Table S6(a,b): Hospital-Level Matched Sample Sizes, Event Counts, Follow-Up Time, and Incidence Rates by Safety Outcome; Supplementary Table S7: Concept IDs for Inclusion and Exclusion Criteria; Supplementary Table S8: Hospital Sites and Their Abbreviations; Supplementary Table S9(a–e): Sex and Age Distribution of the Safety Analysis Cohort, by Hospital; Supplementary Table S10: Sensitivity of Standardized Mean Differences to Masked-Cell Value Assumptions; Supplementary Table S11: Concept IDs for Safety Outcomes; Supplementary Table S12: Concept IDs for Antidepressants and Other Medications; Supplementary Table S13: Hospitals with Inaccessible Covariate Result; Supplementary Table S14: Negative Control Outcome Concept IDs; Supplementary Table S15: Number of Negative Control Outcomes with Estimable Results, by Hospital; Supplementary Table S16(a,b): Uncalibrated and Calibrated Hazard Ratios, 95% Confidence Intervals, and P-values for All Safety Outcomes; Supplementary Figure S1(a–e): Full-Size Sunburst Plots of Antidepressant Treatment Pathways; Supplementary Figure S2(a,b): Secular Prescribing Trends by Sex, Age, and Calendar Period (KDH, KWMC); Supplementary Figure S3: Love Plots of Covariate Balance Before and After Propensity Score Matching; Supplementary Figure S4: Preference Score Distributions Before Propensity Score Matching; Supplementary Figure S5(a–i): Flow Diagrams (Escitalopram vs. Sertraline); Supplementary Figure S6(a–e): Flow Diagrams (Escitalopram vs. Trazodone); Supplementary Figure S7: Forest plot of hazard ratios for multiple clinical outcomes comparing escitalopram and sertraline users in the 5-year time-at-risk (TAR); Supplementary Figure S8: Forest plot of hazard ratios for multiple clinical outcomes comparing escitalopram and trazodone users in the 5-year time-at-risk (TAR); Supplementary Figure S9(a–c): Sensitivity and Subgroup Analysis Using a 0-Day Lag Time-at-Risk, by Sex and Age; Supplementary Figure S10(a–d): Forest plot of hazard ratios across 1-, 3-year Time-at-Risk Window; Supplementary Figure S11(a,b): Sensitivity Analysis Restricted to the 2017–2026 Calendar Period; Supplementary Figure S12: Sensitivity Analysis Excluding Hospitals with Inaccessible Covariate Result; Supplementary Figure S13: Sensitivity Analysis Without the Confidence-Interval-Width Hospital-Exclusion Criterion; Supplementary Figure S14(a,b): Empirical Calibration Diagnostics for Negative Control Outcomes.

## Abbreviations

The following abbreviations are used in this manuscript:

ACE	Angiotensin-converting enzyme
AUMC	Ajou University Medical Center
CDM	Common Data Model
CI	Confidence interval
ECG	Electrocardiogram
EHR	Electronic health record
EMR	Electronic medical record
HR	Hazard ratio
IRB	Institutional Review Board
ITT	Intention-to-treat
MAOI	Monoamine oxidase inhibitor
OMOP	Observational Medical Outcomes Partnership
PSM	Propensity score matching
SMD	Standardized mean difference
SNOMED CT	Systematized Nomenclature of Medicine Clinical Terms
SNRI	Serotonin–norepinephrine reuptake inhibitor
SSRI	Selective serotonin reuptake inhibitor
TAR	Time-at-risk
TCA	Tricyclic antidepressant
