# Supplementary material for: Real-World Antidepressant Prescribing Patterns and Comparative Safety Outcomes of Commonly Prescribed Antidepressants in Patients with Depressive Disorder: A Multicenter OMOP-CDM Study
Source: Pharmaceuticals (Basel). 2026 Aug 24;19(9):1332. doi: 10.3390/ph19091332 (PMC13610068; doi:10.3390/ph19091332)

Supplementary Table S1(a-n). Baseline Characteristics of Patients Included in the Safety Analysis

Values preceded by "≤" indicate that the exact cell count could not be reported because it represented a small number of patients (typically 0–4) subject to privacy-related cell suppression at the contributing hospital; the value shown is the upper bound used in place of the suppressed count, based on the assumption that the true (suppressed) count was 4, the maximum possible value under the "fewer than 5 patients" masking rule. As a sensitivity check, we recalculated all descriptive percentages assuming the opposite extreme (a suppressed count of 0, the minimum possible value) and confirmed that this alternative assumption altered none of the standardized differences: the Std. Diff values reported in this table were identical under both the upper-bound (4) and lower-bound (0) assumptions, because standardized differences were computed from each hospital's underlying result prior to the application of cell-suppression masking and are therefore unaffected by the choice of substitute value used for descriptive display. Standardized differences (Std. Diff) retain their sign to indicate the direction of imbalance (Escitalopram minus comparator, divided by the pooled standard deviation).

Supplementary Table S1a. Atrial fibrillation (Escitalopram vs Sertraline)

	Before PSM Adjustment			After PSM Adjustment		
**Covariate**	**Escitalopram (N=1,257)**	**Sertraline (N=1,474)**	**Std. Diff**	**Escitalopram (N=2,556)**	**Sertraline (N=929)**	**Std. Diff**
**Age & Sex group**						
18-19	2.6	1.3	0.094	2.9	1.5	0.098
20-24	6.0	4.9	0.078	5.5	7.6	-0.079
25-29	3.5	2.5	0.048	2.8	2.9	-0.009
30-34	≤3.2	2.7	0.009	2.8	3.1	-0.022
35-39	4.1	2.7	0.101	3.5	2.7	0.09
40-44	4.0	4.1	-0.002	4.5	4.9	-0.028
45-49	5.2	4.4	0.053	5.0	4.4	0.048
50-54	7.7	5.5	0.088	8.3	6.5	0.072
55-59	6.0	7.9	0.011	7.3	9.3	-0.019
60-64	7.8	8.7	-0.012	8.4	8.9	-0.004
65-69	9.0	9.3	0.012	9.0	9.1	0.018
70-74	9.3	12.4	-0.096	9.8	11.0	-0.04
75-79	12.1	14.1	-0.086	12.0	12.0	-0.012
80-84	11.3	12.0	-0.104	10.6	9.6	-0.031
85-89	5.8	5.5	-0.022	5.4	4.7	0.023
90-94	≤1.7	0.9	-0.059	≤2.0	0.9	-0.047
95-99	≤0.3	≤0.3	-0.008	≤0.6	0.4	-0.043
Female	60.9	65.1	-0.066	64.0	63.3	0.006
**Disease**						
Mild depression	≤52.5	25.4	0.061	39.4	28.0	-0.078
Recurrent depressive disorder	9.7	14.2	-0.078	11.8	13.6	-0.022
Moderate depression	21.2	19.6	-0.119	27.7	19.1	-0.026
Inflammation of specific body systems	28.4	36.1	-0.062	30.4	32.6	0.043
Degenerative disease of the central nervous system	17.9	22.3	-0.223	16.8	15.3	-0.034
Cognitive disorder	≤11.3	9.8	-0.124	≤10.4	8.6	-0.066
Cerebral degeneration	17.3	22.1	-0.238	16.2	15.2	-0.053
Traumatic and/or non-traumatic brain injury	≤1.4	1.0	-0.015	≤1.7	0.7	0.027
Hyperlipidemia	13.9	22.4	-0.186	16.4	16.4	0.018
Alzheimer's dementia	16.5	20.0	-0.206	15.0	13.8	-0.039
Cerebral infarction	6.8	15.4	-0.228	7.6	8.9	-0.024
Heart disease	10.0	11.5	-0.006	12.1	9.0	0.136
**Drugs**						
Other ophthalmologicals	38.2	44.9	-0.133	41.6	38.7	0.064
Proton pump inhibitors	14.8	18.3	-0.082	17.1	17.5	0.003
ACE inhibitors	1.3	2.5	0.017	≤1.7	1.7	0.058
Drugs for constipation	16.8	20.8	-0.082	18.9	18.7	0.021
Vitamin B1, plain	13.3	21.9	-0.18	16.2	16.0	0.04
Beta blocking agents	17.3	17.8	-0.006	18.7	16.1	0.067
Antipruritics, incl. antihistamines, anesthetics, etc.	14.8	19.0	-0.038	17.3	18.3	0.038
Acetaminophen	22.5	20.4	0.023	22.0	20.2	0.044
Antiepileptics	18.7	18.0	-0.01	17.7	16.1	0.039
Other plain vitamin preparations	12.0	21.4	-0.184	14.9	15.5	0.034
Antianemic preparations	13.6	15.7	-0.076	15.5	13.0	0.059
Statins (HMG CoA reductase inhibitors)	15.5	22.2	-0.124	17.8	16.1	0.073
Angiotensin II antagonists	11.6	16.0	-0.076	14.8	11.8	0.114
Atypical antipsychotics	5.1	4.2	0.128	6.4	7.5	0.005
Amlodipine	6.7	10.2	-0.122	8.1	7.3	0.024
Aspirin	13.8	17.9	-0.123	13.7	13.2	0.022
Metformin	7.2	8.3	-0.067	7.0	7.3	-0.02

Included hospitals: KWMC, DCMC. SEJONG_ICN contributed to the forest plot analysis but could not be included in this table due to subsequent inaccessibility of results.

Supplementary Table S1b. Cardiac arrest (Escitalopram vs Sertraline)

Covariate	Before PSM Adjustment			After PSM Adjustment		
	Escitalopram (N=3,478)	Sertraline (N=1,833)	Std. Diff	Escitalopram (N=3,427)	Sertraline (N=1,175)	Std. Diff
**Age & Sex group**						
18-19	≤2.3	2.1	0.003	≤2.4	2.0	0.018
20-24	5.9	5.5	0.039	6.0	6.8	-0.007
25-29	3.4	2.8	0.031	2.8	2.9	0.002
30-34	≤3.8	3.3	0.037	3.5	3.9	0.001
35-39	4.2	3.3	0.075	4.2	3.7	0.052
40-44	4.7	5.1	0.019	4.6	5.7	-0.026
45-49	5.5	5.5	0.027	5.2	5.6	0.005
50-54	8.0	6.7	0.07	8.0	7.8	0.004
55-59	5.9	7.4	-0.002	6.3	8.3	-0.027
60-64	8.0	9.2	-0.019	8.3	8.8	0
65-69	8.9	9.3	0.005	9.5	9.3	-0.004
70-74	9.4	11.7	-0.046	10.0	10.6	0.003
75-79	11.9	12.6	-0.068	11.7	11.4	-0.009
80-84	10.7	10.3	-0.07	10.1	8.5	-0.01
85-89	5.8	4.4	-0.008	≤5.8	3.9	0.027
90-94	≤2.0	1.6	-0.043	≤2.2	1.5	-0.015
95-99	-	-	-	-	-	-
Female	62.2	63.9	0.018	64.5	65.3	-0.007
**Disease**						
Mild depression	46.1	19.8	0.101	34.2	23.1	-0.061
Recurrent depressive disorder	8.9	12.3	-0.07	10.8	12.1	-0.016
Moderate depression	21.3	18.1	-0.016	26.3	19.9	-0.004
Inflammation of specific body systems	28.6	33.1	-0.022	29.5	31.2	0.012
Degenerative disease of the central nervous system	16.4	17.5	-0.149	14.9	12.5	-0.011
Cognitive disorder	≤10.2	8.0	-0.073	10.9	6.8	0.022
Cerebral degeneration	15.9	17.3	-0.164	14.6	12.4	-0.022
Traumatic and/or non-traumatic brain injury	≤1.5	1.0	0.011	≤1.4	0.7	0.026
Hyperlipidemia	13.5	17.9	-0.13	15.1	14.1	0.001
Alzheimer's dementia	17.2	20.4	-0.206	15.7	14.4	-0.049
Cerebral infarction	7.8	12.9	-0.112	8.4	9.2	-0.01
Heart disease	12.1	13.3	-0.029	14.0	10.8	0.08
**Drugs**						
Other ophthalmologicals	39.1	43.6	-0.081	40.4	37.7	0.029
Proton pump inhibitors	14.9	18.0	-0.088	17.1	16.8	0
ACE inhibitors	2.0	2.5	0.076	≤2.0	2.3	0.032
Drugs for constipation	18.0	20.3	-0.028	19.6	18.8	0.01
Vitamin B1, plain	13.9	20.1	-0.123	14.8	15.2	0.002
Beta blocking agents	18.0	18.3	-0.016	17.6	16.5	0.023
Antipruritics, incl. antihistamines, anesthetics, etc.	15.9	18.7	0.003	17.9	18.6	0.032
Acetaminophen	21.7	20.0	-0.006	20.7	18.7	0.02
Antiepileptics	19.2	19.6	-0.033	17.7	17.9	0.003
Other plain vitamin preparations	12.8	20.0	-0.131	13.9	15.1	-0.003
Antianemic preparations	13.5	15.6	-0.101	13.8	12.2	0.024
Statins (HMG CoA reductase inhibitors)	16.5	20.8	-0.081	18.1	16.4	0.054
Angiotensin II antagonists	12.9	15.2	-0.029	14.3	12.6	0.05
Atypical antipsychotics	6.3	6.1	0.091	7.5	6.3	0.103
Amlodipine	7.7	9.3	-0.036	8.3	8.2	0.003
Aspirin	13.5	15.4	-0.075	13.2	12.9	-0.003
Metformin	7.1	7.8	-0.069	7.3	7.0	-0.011

Included hospitals: AUMC, DCMC and KWMC

Supplementary Table S1c. Circulatory system (Escitalopram vs Sertraline)

	Before PSM Adjustment			After PSM Adjustment		
**Covariate**	**Escitalopram (N=7,734)**	**Sertraline (N=1,833)**	**Std. Diff**	**Escitalopram (N=7,918)**	**Sertraline (N=1,501)**	**Std. Diff**
**Age & Sex group**						
18-19	≤3.8	2.7	0.084	≤4.4	4.1	0.026
20-24	≤8.4	7.0	0.084	≤9.0	8.9	0.024
25-29	≤5.5	3.6	0.116	≤5.7	4.6	0.105
30-34	≤4.8	4.3	-0.01	≤4.9	3.8	0.005
35-39	≤5.4	4.0	0.089	≤5.7	4.3	0.094
40-44	≤6.1	6.1	0.031	≤6.4	6.8	-0.018
45-49	≤6.5	6.8	0.016	≤6.6	6.7	-0.015
50-54	≤8.3	8.4	-0.068	≤8.7	8.2	-0.085
55-59	≤6.6	9.2	-0.054	≤7.2	8.5	-0.048
60-64	8.3	9.3	0.003	8.5	8.7	0.034
65-69	8.6	9.2	0.007	9.2	9.3	0.008
70-74	≤8.3	9.8	-0.062	≤9.0	9.8	-0.017
75-79	≤8.8	8.8	-0.083	≤8.4	7.5	-0.017
80-84	≤7.8	8.0	-0.079	≤6.6	6.1	0.01
85-89	≤4.5	3.1	-0.029	≤4.7	2.4	0.018
90-94	≤2.1	1.0	0.018	≤2.2	0.9	0.049
95-99	-	-	-	-	-	-
Female	62.3	65.6	-0.028	64.3	64.7	-0.009
**Disease**						
Mild depression	≤34.4	16.3	-0.057	≤23.7	16.3	-0.026
Recurrent depressive disorder	≤9.4	6.4	0.083	≤10.4	≤7.1	0.061
Moderate depression	22.1	19.7	0.092	24.5	20.8	-0.013
Inflammation of specific body systems	24.7	25.4	-0.032	24.4	23.4	0.007
Degenerative disease of the central nervous system	≤12.7	14.0	-0.173	≤11.6	9.5	0.027
Cognitive disorder	≤7.5	6.4	-0.106	≤7.2	6.2	-0.026
Cerebral degeneration	≤12.4	13.8	-0.177	≤11.3	9.4	0.018
Traumatic and/or non-traumatic brain injury	≤1.8	≤1.1	0.033	≤2.3	1.0	0.046
Hyperlipidemia	≤6.2	6.3	-0.063	≤6.9	4.7	0.047
Alzheimer's dementia	≤11.7	12.2	-0.151	≤10.3	8.4	0.026
Cerebral infarction	≤4.5	6.1	-0.112	≤4.9	4.2	0
Heart disease	≤4.5	5.3	-0.027	≤5.3	4.1	0.042
**Drugs**						
Other ophthalmologicals	32.9	36.8	-0.077	33.0	32.0	0.008
Proton pump inhibitors	12.0	16.2	-0.079	13.1	13.3	0.005
ACE inhibitors	≤1.4	≤1.1	0.06	≤2.4	1.2	0.062
Drugs for constipation	≤15.7	17.7	-0.011	≤16.4	15.7	0.013
Vitamin B1, plain	≤10.0	13.8	-0.087	≤11.5	11.1	-0.017
Beta blocking agents	12.9	11.5	0.007	≤13.0	12.7	0.009
Antipruritics, incl. antihistamines, anesthetics, etc.	12.7	14.0	0.001	≤13.7	12.2	0.053
Acetaminophen	17.7	18.9	-0.072	15.9	16.1	0.016
Antiepileptics	≤17.3	21.0	-0.121	≤16.4	17.6	-0.006
Other plain vitamin preparations	≤8.7	12.4	-0.067	≤10.0	10.1	-0.003
Antianemic preparations	≤9.5	11.8	-0.064	≤10.5	9.8	-0.003
Statins (HMG CoA reductase inhibitors)	≤9.3	13.6	-0.134	≤10.1	9.4	0.017
Angiotensin II antagonists	≤4.0	6.1	-0.06	≤4.8	4.2	0.008
Atypical antipsychotics	≤7.4	6.0	0.084	≤8.7	7.8	0.043
Amlodipine	≤3.8	5.4	-0.014	≤4.5	4.8	0.016
Aspirin	≤7.0	9.5	-0.106	≤7.0	6.5	0.025
Metformin	≤4.0	4.2	-0.015	≤4.7	3.6	0.04

Included hospitals: AUMC, CHNUH, DCMC, EUMC, HNUH, KDH, KHMC, KWMC, KYUH, SCHBC and UUH. CAUHS contributed to the forest plot analysis but could not be included in this table due to subsequent inaccessibility of results.

Supplementary Table S1d. Gastrointestinal system (Escitalopram vs Sertraline)

	Before PSM Adjustment			After PSM Adjustment		
**Covariate**	**Escitalopram (N=4,127)**	**Sertraline (N=1,626)**	**Std. Diff**	**Escitalopram (N=3,824)**	**Sertraline (N=1,123)**	**Std. Diff**
**Age & Sex group**						
18-19	≤2.5	2.6	-0.026	≤3.1	3.3	-0.056
20-24	≤6.7	5.7	0.068	≤7.1	6.9	0.013
25-29	4.0	3.0	0.115	≤3.7	3.2	0.049
30-34	≤3.9	3.9	-0.013	≤4.2	4.3	-0.042
35-39	4.7	3.4	0.092	≤4.2	4.1	0.024
40-44	≤5.1	5.0	0.045	≤5.2	5.9	-0.04
45-49	5.4	5.5	0.058	≤5.5	5.8	0
50-54	7.6	7.0	-0.019	≤7.9	6.8	-0.019
55-59	≤5.8	8.0	-0.072	≤6.7	8.3	-0.078
60-64	8.9	9.3	0.06	9.0	9.0	0.075
65-69	9.4	8.9	0.031	10.1	9.1	0.045
70-74	≤9.1	10.6	-0.056	≤9.2	8.8	0.029
75-79	≤11.5	12.1	-0.097	≤12.6	11.0	-0.015
80-84	≤10.8	11.1	-0.071	≤10.7	9.9	0.022
85-89	≤4.8	4.7	-0.045	≤4.6	3.7	-0.001
90-94	≤2.4	2.0	-0.01	≤2.1	≤1.5	0.051
95-99	-	-	-	-	-	-
Female	62.3	65.0	-0.006	62.9	65.5	-0.024
**Disease**						
Mild depression	≤44.1	26.3	-0.031	≤34.1	24.5	-0.009
Recurrent depressive disorder	≤7.8	4.8	0.085	≤9.2	5.6	0.075
Moderate depression	23.0	19.8	0.069	26.5	22.6	-0.059
Inflammation of specific body systems	26.8	29.6	-0.002	26.8	27.9	0.006
Degenerative disease of the central nervous system	16.8	18.6	-0.119	16.0	13.6	0.084
Cognitive disorder	≤9.9	9.4	-0.094	≤9.8	7.2	0.024
Cerebral degeneration	16.4	18.5	-0.126	15.6	13.4	0.081
Traumatic and/or non-traumatic brain injury	1.8	1.0	0.094	≤1.8	0.9	0.091
Hyperlipidemia	≤11.5	15.1	-0.193	≤12.0	8.8	0.073
Alzheimer's dementia	15.7	17.0	-0.114	14.6	12.4	0.076
Cerebral infarction	≤7.3	12.4	-0.154	≤8.8	7.6	0.049
Heart disease	11.1	11.5	-0.004	12.1	8.6	0.113
**Drug**						
Other ophthalmologicals	35.7	37.5	-0.046	35.6	31.2	0.048
Proton pump inhibitors	13.1	19.3	-0.138	14.9	14.5	0.027
ACE inhibitors	≤1.8	2.0	-0.016	≤1.8	1.6	-0.012
Drugs for constipation	16.3	18.8	0.002	16.9	16.4	0.033
Vitamin B1, plain	12.1	14.1	0.021	12.8	11.2	0.025
Beta blocking agents	16.9	15.8	0.004	16.9	13.8	0.088
Antipruritics, incl. antihistamines, anesthetics, etc.	14.2	16.0	-0.046	14.7	13.7	0.007
Acetaminophen	19.5	19.8	-0.097	17.2	16.7	-0.039
Antiepileptics	17.5	21.5	-0.162	15.6	16.5	-0.04
Other plain vitamin preparations	10.7	12.0	0.02	11.1	10.2	0.017
Antianemic preparations	11.6	13.8	-0.034	12.6	10.1	0.082
Statins (HMG CoA reductase inhibitors)	14.9	22.4	-0.206	16.0	13.5	0.098
Angiotensin II antagonists	11.4	15.6	-0.122	12.4	11.7	0.031
Atypical antipsychotics	≤4.3	3.9	0.023	≤5.5	3.6	0.066
Amlodipine	≤8.1	10.2	-0.062	≤9.4	7.9	0.047
Aspirin	12.7	15.0	-0.057	11.7	9.9	0.095
Metformin	≤7.2	8.0	-0.033	≤8.1	5.6	0.109

Included hospitals: AUMC, KDH, KHMC, KWMC, and SCHBC. CAUHS contributed to the forest plot analysis but could not be included in this table due to subsequent inaccessibility of results.

Supplementary Table S1e. Heart failure (Escitalopram vs Sertraline)

Covariate	Before PSM Adjustment			After PSM Adjustment		
	Escitalopram (N=4,580)	Sertraline (N=1,781)	Std. Diff	Escitalopram (N=4,387)	Sertraline (N=1,166)	Std. Diff
**Age & Sex group**						
18-19	3.0	2.3	0.121	3.2	3.8	0.055
20-24	7.0	6.1	0.132	6.5	9.6	-0.065
25-29	4.4	3.3	0.127	4.7	6.3	0.016
30-34	≤3.1	3.0	-0.019	≤2.8	3.4	-0.046
35-39	4.3	2.8	0.119	4.2	3.1	0.092
40-44	3.9	4.3	-0.002	≤4.1	4.9	-0.042
45-49	5.2	4.5	0.048	5.2	4.2	0.052
50-54	7.2	6.3	-0.001	7.4	6.7	-0.012
55-59	6.0	8.3	-0.038	6.7	8.3	-0.033
60-64	7.8	8.4	-0.025	7.8	7.7	0.002
65-69	9.0	9.5	-0.008	9.3	8.2	0.038
70-74	9.3	11.9	-0.086	10.5	10.5	-0.005
75-79	12.0	13.3	-0.09	11.5	10.8	-0.009
80-84	10.7	10.3	-0.103	10.4	7.4	0.037
85-89	≤5.4	4.3	-0.043	≤4.4	3.4	0.007
90-94	≤2.0	1.2	0.01	≤2.3	1.0	0.047
95-99	≤0.3	≤0.3	-0.008	≤0.6	0.4	-0.054
Female	60.2	65.5	-0.127	62.7	62.9	-0.019
**Disease**						
Mild depression	47.4	22.4	0.044	34.5	22.2	0.008
Recurrent depressive disorder	8.7	8.8	0.018	≤11.2	8.8	0.032
Moderate depression	19.2	17.6	-0.09	23.7	17.6	-0.077
Inflammation of specific body systems	28.2	32.3	-0.09	29.9	26.9	0.052
Degenerative disease of the central nervous system	17.8	19.6	-0.183	16.8	12.9	0.028
Cognitive disorder	≤3.2	5.1	-0.152	≤3.6	3.2	-0.028
Cerebral degeneration	17.4	19.3	-0.188	16.4	12.8	0.023
Traumatic and/or non-traumatic brain injury	≤1.4	0.9	0.005	≤1.5	≤0.6	0.032
Hyperlipidemia	13.2	18.8	-0.228	15.0	12.0	0.048
Alzheimer's dementia	16.3	16.6	-0.161	15.1	11.0	0.03
Cerebral infarction	7.4	13.9	-0.235	≤8.5	7.3	0.018
Heart disease	9.1	9.9	-0.046	10.3	6.9	0.105
**Drug**						
Other ophthalmologicals	38.2	42.2	-0.079	41.4	34.0	0.109
Proton pump inhibitors	15.2	19.1	-0.084	17.6	16.5	0.021
ACE inhibitors	≤1.2	1.5	0.009	≤1.7	1.1	0.039
Drugs for constipation	18.0	22.4	-0.028	19.9	18.6	0.065
Vitamin B1, plain	13.3	18.5	-0.061	15.4	12.9	0.096
Beta blocking agents	17.2	15.9	0.057	17.4	18.1	0.031
Antipruritics, incl. antihistamines, anesthetics, etc.	14.1	16.9	-0.064	16.0	14.8	0.023
Acetaminophen	21.2	19.9	-0.06	20.5	16.4	0.036
Antiepileptics	18.3	19.7	-0.046	17.3	17.0	0.037
Other plain vitamin preparations	12.0	17.5	-0.082	14.1	12.4	0.065
Antianemic preparations	13.2	15.3	-0.063	14.6	11.3	0.058
Statins (HMG CoA reductase inhibitors)	14.8	22.7	-0.248	16.0	13.7	0.044
Angiotensin II antagonists	11.2	15.5	-0.141	12.5	9.6	0.084
Atypical antipsychotics	6.1	4.6	0.176	7.3	8.7	0.031
Amlodipine	7.5	10.5	-0.095	8.8	6.6	0.082
Aspirin	12.6	15.5	-0.144	12.8	9.8	0.084
Metformin	7.0	7.9	-0.066	7.2	6.2	0.028

Included hospitals: DCMC, EUMC, GNUH, KHMC, and KWMC.

Supplementary Table S1f. Hematologic side effects (Escitalopram vs Sertraline)

Covariate	Before PSM Adjustment			After PSM Adjustment		
	Escitalopram (N=7,785)	Sertraline (N=2,399)	Std. Diff	Escitalopram (N=7,258)	Sertraline (N=1,692)	Std. Diff
**Age & Sex group**						
18-19	≤2.8	2.7	0.02	≤3.0	3.8	-0.032
20-24	7.1	5.7	0.11	7.4	7.8	0.02
25-29	4.2	3.3	0.089	4.0	5.0	0.009
30-34	≤3.9	3.3	0.026	≤3.6	3.8	-0.023
35-39	4.2	3.3	0.068	≤4.2	4.0	0.029
40-44	5.0	4.7	0.046	5.0	5.3	-0.013
45-49	5.4	5.0	0.022	≤5.3	5.6	-0.02
50-54	≤7.2	6.7	-0.01	≤7.1	7.2	-0.05
55-59	6.4	7.9	-0.02	6.8	8.4	-0.038
60-64	8.1	8.6	-0.008	8.4	8.2	0.013
65-69	8.8	9.2	-0.01	9.2	8.8	0.014
70-74	9.4	11.1	-0.043	9.9	10.1	0.008
75-79	11.5	12.2	-0.06	11.1	10.4	0.009
80-84	≤9.5	9.8	-0.116	≤8.9	7.7	-0.031
85-89	≤5.0	4.7	-0.048	≤4.6	3.0	0.044
90-94	≤2.0	1.6	-0.037	≤2.2	1.1	0.023
95-99	≤0.3	≤0.3	-0.023	≤0.6	0.4	-0.043
Female	61.1	65.1	-0.064	62.5	65.8	-0.07
**Disease**						
Mild depression	39.0	21.6	-0.03	≤29.6	19.3	-0.009
Recurrent depressive disorder	≤7.4	6.4	-0.005	≤8.8	6.4	0.025
Moderate depression	20.5	18.2	0.014	23.0	20.2	-0.08
Inflammation of specific body systems	28.6	31.0	-0.03	29.3	28.4	0.032
Degenerative disease of the central nervous system	15.4	20.1	-0.217	14.8	11.6	0.053
Cognitive disorder	≤8.5	6.9	-0.113	≤7.7	5.3	-0.011
Cerebral degeneration	15.0	20.0	-0.224	14.4	11.5	0.048
Traumatic and/or non-traumatic brain injury	≤1.7	0.8	0.049	≤1.9	≤0.8	0.061
Hyperlipidemia	≤12.0	16.1	-0.2	≤13.1	10.2	0.024
Alzheimer's dementia	14.1	17.7	-0.202	13.3	9.9	0.058
Cerebral infarction	7.4	11.0	-0.124	≤7.9	7.0	0.009
Heart disease	11.4	11.9	-0.022	12.3	9.1	0.083
**Drugs**						
Other ophthalmologicals	37.0	38.8	-0.049	37.1	32.7	0.065
Proton pump inhibitors	14.4	18.1	-0.091	16.4	14.9	0.042
ACE inhibitors	≤2.1	2.1	0.013	≤2.3	1.7	0.027
Drugs for constipation	17.9	20.1	0.002	17.8	17.2	0.044
Vitamin B1, plain	12.9	16.2	-0.048	12.8	11.9	0.024
Beta blocking agents	17.7	15.8	0.052	17.3	17.6	0.024
Antipruritics, incl. antihistamines, anesthetics, etc.	13.9	15.1	-0.021	14.7	13.7	0.022
Acetaminophen	19.3	19.2	-0.07	18.4	16.4	-0.002
Antiepileptics	17.7	19.8	-0.072	17.3	16.5	0.027
Other plain vitamin preparations	11.4	14.8	-0.068	11.6	11.1	0.007
Antianemic preparations	10.3	12.1	-0.097	10.4	8.5	0.031
Statins (HMG CoA reductase inhibitors)	15.1	21.1	-0.181	16.6	13.7	0.07
Angiotensin II antagonists	11.7	14.7	-0.099	13.2	10.7	0.062
Atypical antipsychotics	6.4	5.0	0.127	7.0	6.3	0.072
Amlodipine	8.0	9.9	-0.042	8.8	6.9	0.075
Aspirin	12.3	14.0	-0.088	12.5	10.0	0.047
Metformin	7.0	7.5	-0.045	7.2	5.7	0.063

Included hospitals: AUMC, DCMC, EUMC, KDH, KHMC, KWMC, and MJH

Supplementary Table S1g. Hypersensitivity (Escitalopram vs Sertraline)

Covariate	Before PSM Adjustment			After PSM Adjustment		
	Escitalopram (N=3,792)	Sertraline (N=601)	Std. Diff	Escitalopram (N=3,576)	Sertraline (N=502)	Std. Diff
**Age & Sex group**						
18-19	≤3.8	3.3	0.04	≤4.0	4.6	0.012
20-24	9.3	6.6	0.119	10.6	8.0	0.122
25-29	6.1	3.9	0.115	≤6.4	6.1	0.039
30-34	≤5.6	4.0	0.039	≤5.2	4.5	-0.018
35-39	4.9	3.9	0.028	5.0	4.4	0.011
40-44	8.0	5.8	0.058	6.0	5.9	-0.021
45-49	5.9	6.2	-0.045	≤5.6	5.9	-0.063
50-54	7.9	7.8	-0.037	7.2	7.0	-0.019
55-59	6.8	8.3	-0.047	6.4	8.7	-0.083
60-64	8.3	9.0	-0.047	8.2	8.0	-0.011
65-69	8.9	9.5	-0.019	9.8	9.4	0.006
70-74	8.9	9.7	-0.017	9.6	9.2	0.02
75-79	7.6	9.6	-0.058	8.2	8.5	-0.003
80-84	5.2	7.3	-0.076	5.6	6.5	-0.031
85-89	≤2.2	3.8	-0.097	≤2.4	2.6	-0.043
90-94	≤2.4	1.3	0.03	≤2.9	0.6	0.12
95-99	-	-	-	-	-	-
Female	67.2	65.8	0.008	66.7	69.6	-0.098
**Disease**						
Mild depression	9.9	10.3	-0.005	≤9.8	10.7	-0.052
Recurrent depressive disorder	≤5.3	3.1	0.049	≤6.4	3.1	0.076
Moderate depression	23.5	18.8	0.121	20.9	19.6	0.006
Inflammation of specific body systems	27.1	24.3	0.05	25.9	21.9	0.079
Degenerative disease of the central nervous system	6.6	12.7	-0.132	8.5	8.0	0.064
Cognitive disorder	≤3.8	3.9	-0.025	≤4.6	3.5	0.031
Cerebral degeneration	6.3	12.3	-0.157	8.0	7.7	0.036
Traumatic and/or non-traumatic brain injury	2.1	1.1	0.094	≤2.3	0.9	0.078
Hyperlipidemia	3.5	6.0	-0.106	3.8	4.1	0
Alzheimer's dementia	5.0	9.8	-0.135	6.4	5.5	0.055
Cerebral infarction	4.9	5.5	-0.023	≤4.0	4.0	0.001
Heart disease	8.8	8.3	0.032	9.2	6.6	0.112
**Drug**						
Other ophthalmologicals	35.3	37.9	-0.073	31.7	27.9	0.089
Proton pump inhibitors	14.0	19.1	-0.118	15.5	13.0	0.078
ACE inhibitors	≤3.9	1.9	0.042	≤3.6	1.7	0.056
Drugs for constipation	21.9	22.8	0.003	20.7	17.3	0.118
Vitamin B1, plain	14.1	16.4	-0.047	12.4	11.6	0.041
Beta blocking agents	16.3	13.9	0.079	16.2	18.2	0.006
Antipruritics, incl. antihistamines, anesthetics, etc.	17.4	16.8	-0.012	16.7	13.3	0.081
Acetaminophen	15.4	19.5	-0.118	13.9	13.9	0.003
Antiepileptics	19.3	23.8	-0.126	19.9	19.2	0.027
Other plain vitamin preparations	12.7	13.9	-0.038	10.9	10.4	0.023
Antianemic preparations	9.3	14.5	-0.157	9.6	8.7	0.054
Statins (HMG CoA reductase inhibitors)	13.0	16.4	-0.084	14.3	11.5	0.095
Angiotensin II antagonists	10.0	11.4	-0.048	10.4	10.0	0.007
Atypical antipsychotics	10.5	7.0	0.092	10.0	7.4	0.098
Amlodipine	8.8	8.0	0.035	8.2	6.4	0.07
Aspirin	9.4	11.3	-0.045	9.6	7.9	0.072
Metformin	5.6	5.7	0.011	6.2	5.1	0.062

Included hospitals: AUMC, EUMC, and KDH

Supplementary Table S1h. Psycho-neurological system (Escitalopram vs Sertraline)

Covariate	Before PSM Adjustment			After PSM Adjustment		
	Escitalopram (N=6,230)	Sertraline (N=931)	Std. Diff	Escitalopram (N=5,934)	Sertraline (N=941)	Std. Diff
**Age & Sex group**						
18-19	≤5.8	3.2	0.159	≤5.3	≤5.8	0.026
20-24	≤8.5	6.2	0.085	≤9.5	8.1	0.033
25-29	≤6.6	3.8	0.114	≤6.5	5.6	0.048
30-34	≤5.1	3.8	-0.001	≤5.5	4.7	-0.037
35-39	5.8	3.8	0.103	≤6.2	5.1	0.061
40-44	≤6.6	4.8	0.072	≤6.4	6.4	-0.001
45-49	≤7.4	5.8	0.073	≤7.5	6.5	0.023
50-54	≤6.5	7.6	-0.062	≤6.3	7.4	-0.105
55-59	≤7.2	7.8	-0.038	≤6.9	7.7	-0.036
60-64	≤8.8	8.4	-0.015	≤8.6	7.8	-0.009
65-69	≤8.4	8.5	-0.017	≤9.0	8.5	-0.016
70-74	≤9.4	10.0	-0.054	≤10.4	9.1	0.002
75-79	≤9.5	11.3	-0.08	≤10.1	8.1	0.057
80-84	≤6.9	9.0	-0.119	≤8.1	6.0	0.033
85-89	≤3.5	4.9	-0.07	≤4.2	2.9	0.026
90-94	≤3.0	2.0	-0.034	≤4.0	0.7	0.085
95-99	-	-	-	-	-	-
Female	61.4	63.3	-0.05	61.3	63.6	-0.063
**Disease**						
Mild depression	≤19.1	26.6	-0.169	≤21.3	17.1	0.093
Recurrent depressive disorder	≤5.5	3.0	0.1	≤7.2	3.1	0.124
Moderate depression	19.5	16.4	0.138	17.4	19.7	-0.05
Inflammation of specific body systems	30.4	32.0	0.009	29.1	31.3	0.005
Degenerative disease of the central nervous system	≤11.0	18.0	-0.233	≤13.7	8.0	0.116
Cognitive disorder	≤4.2	5.9	-0.111	≤5.4	4.5	-0.021
Cerebral degeneration	≤10.8	17.9	-0.236	≤13.6	7.8	0.116
Traumatic and/or non-traumatic brain injury	≤2.3	≤0.8	0.064	≤3.1	≤0.9	0.04
Hyperlipidemia	≤8.2	13.4	-0.186	≤9.2	9.0	0.003
Alzheimer's dementia	≤9.8	16.3	-0.215	≤12.3	7.0	0.121
Cerebral infarction	≤6.8	10.0	-0.142	≤6.9	6.6	-0.005
Heart disease	≤9.3	11.6	-0.086	≤8.6	7.1	0.053
**Drug**						
Other ophthalmologicals	34.6	37.8	-0.094	31.5	32.4	-0.036
Proton pump inhibitors	15.4	19.2	-0.048	16.1	15.2	0.081
ACE inhibitors	≤4.0	2.4	-0.014	≤4.1	2.0	-0.013
Drugs for constipation	≤19.6	21.4	-0.074	≤18.7	17.3	0.014
Vitamin B1, plain	≤13.8	15.3	-0.028	≤11.7	11.5	0.002
Beta blocking agents	≤16.2	13.3	0.029	≤15.9	14.4	0.029
Antipruritics, incl. antihistamines, anesthetics, etc.	14.3	14.7	0.043	13.1	13.9	0.029
Acetaminophen	15.7	17.8	-0.053	14.5	14.3	0.013
Antiepileptics	≤17.1	20.3	-0.097	≤17.8	18.4	-0.029
Other plain vitamin preparations	≤12.4	13.3	-0.015	≤10.2	9.9	0.025
Antianemic preparations	≤9.8	13.2	-0.074	≤9.7	9.3	0.033
Statins (HMG CoA reductase inhibitors)	≤12.5	21.5	-0.261	≤13.2	12.6	0.03
Angiotensin II antagonists	≤10.4	15.1	-0.194	≤10.8	10.1	-0.015
Atypical antipsychotics	≤8.9	5.6	0.103	≤8.7	8.5	0.022
Amlodipine	≤9.7	10.5	-0.094	≤9.9	7.2	0.044
Aspirin	10.1	14.0	-0.11	9.7	9.3	0.042
Metformin	≤6.6	7.7	-0.051	≤6.9	5.4	0.054

Included hospitals: AUMC, EUMC, KDH, KHMC, KYUH, MJH, SCHBC, SCHSU. CAUHS, GNUH, and SEJONG_ICN contributed to the forest plot analysis but could not be included in this table due to subsequent inaccessibility of results.

Supplementary Table S1i. QT prolongation (Escitalopram vs Sertraline)

Covariate	Before PSM Adjustment			After PSM Adjustment		
	Escitalopram (N=4,294)	Sertraline (N=1,774)	Std. Diff	Escitalopram (N=4,748)	Sertraline (N=1,273)	Std. Diff
**Age & Sex group**						
18-19	3.4	1.8	0.173	3.8	3.9	0.058
20-24	6.2	4.9	0.092	6.4	7.2	0.002
25-29	3.9	2.8	0.096	≤3.3	3.5	0.022
30-34	3.5	2.8	0.041	≤3.0	3.3	0
35-39	4.5	3.5	0.095	4.8	4.1	0.078
40-44	4.3	4.3	0.017	≤4.7	5.8	-0.055
45-49	5.6	5.1	0.042	5.9	6.1	0.006
50-54	7.4	6.2	0.011	≤6.9	7.6	-0.076
55-59	6.1	7.5	0.002	6.6	8.3	-0.005
60-64	8.1	8.7	-0.006	8.3	8.2	0.017
65-69	8.8	8.7	0.007	9.3	8.9	0.013
70-74	9.2	10.8	-0.068	9.6	9.9	-0.011
75-79	≤9.3	13.9	-0.182	≤9.0	9.8	-0.05
80-84	≤10.4	11.5	-0.153	≤10.1	8.0	-0.011
85-89	5.8	6.4	-0.058	5.0	4.1	0.06
90-94	≤2.0	2.1	-0.07	≤1.8	1.3	-0.008
95-99	≤0.5	≤0.5	-0.02	≤0.9	≤0.4	0.008
Female	60.1	64.6	-0.107	61.9	62.4	-0.022
**Disease**						
Mild depression	≤47.1	28.6	-0.108	≤35.2	23.5	-0.034
Recurrent depressive disorder	≤8.4	8.4	-0.055	≤9.6	8.6	-0.001
Moderate depression	20.8	16.6	0.03	26.2	19.0	0.008
Inflammation of specific body systems	29.6	37.1	-0.071	32.4	33.0	0.056
Degenerative disease of the central nervous system	≤16.4	24.3	-0.364	≤15.5	12.0	-0.004
Cognitive disorder	≤9.8	9.0	-0.178	≤9.1	6.9	-0.058
Cerebral degeneration	≤16.0	24.1	-0.369	≤15.2	11.9	-0.008
Traumatic and/or non-traumatic brain injury	≤1.4	0.8	0.005	≤1.4	≤0.6	0.045
Hyperlipidemia	13.3	20.5	-0.205	14.6	13.8	0.012
Alzheimer's dementia	≤15.1	22.6	-0.352	≤14.0	10.9	-0.005
Cerebral infarction	6.5	12.4	-0.164	7.4	8.5	-0.033
Heart disease	≤9.2	12.3	-0.106	≤10.5	8.7	0.037
**Drug**						
Other ophthalmologicals	37.8	40.6	-0.086	39.9	37.6	0.006
Proton pump inhibitors	14.6	19.4	-0.114	16.4	17.8	-0.035
ACE inhibitors	1.4	2.1	0.032	≤1.8	1.8	0.028
Drugs for constipation	≤15.8	17.3	-0.108	≤16.3	17.2	-0.102
Vitamin B1, plain	13.1	17.8	-0.116	14.2	15.0	-0.037
Beta blocking agents	17.4	16.0	0.046	18.7	16.3	0.057
Antipruritics, incl. antihistamines, anesthetics, etc.	13.8	13.9	0.002	15.2	14.0	0.04
Acetaminophen	21.3	19.8	-0.023	20.6	19.5	0.005
Antiepileptics	18.7	18.9	0.01	17.6	18.6	0.022
Other plain vitamin preparations	12.0	17.5	-0.1	13.5	14.3	-0.004
Antianemic preparations	12.1	12.4	-0.085	≤12.8	11.4	-0.036
Statins (HMG CoA reductase inhibitors)	14.6	23.0	-0.253	16.1	14.4	0.014
Angiotensin II antagonists	11.0	16.9	-0.191	12.7	10.7	0.039
Atypical antipsychotics	5.8	4.1	0.184	6.9	7.3	0.057
Amlodipine	≤6.8	11.4	-0.185	≤8.3	7.2	-0.003
Aspirin	≤12.3	15.3	-0.189	≤11.9	10.6	0.007
Metformin	≤7.0	8.7	-0.107	≤7.7	6.6	0.028

Included hospitals: DCMC, KWMC, KYUH, and MJH. GNUH and SEJONG_ICN contributed to the forest plot analysis but could not be included in this table due to subsequent inaccessibility of results.

Supplementary Table S1j. Circulatory system (Escitalopram vs Trazodone)

Covariate	Before PSM Adjustment			After PSM Adjustment		
	Escitalopram (N=2,089)	Trazodone (N=3,117)	Std. Diff	Escitalopram (N=755)	Trazodone (N=2,628)	Std. Diff
**Age & Sex group**						
18-19	2.2	3.9	-0.072	2.6	4.6	-0.055
20-24	6.0	7.6	-0.004	≤7.4	7.0	0.029
25-29	4.9	4.3	0.063	≤4.6	4.6	0.017
30-34	≤4.5	4.0	-0.015	≤4.5	4.3	-0.031
35-39	≤5.2	3.4	0.056	≤4.5	3.6	0.017
40-44	≤6.1	4.5	0.073	≤5.6	5.8	-0.039
45-49	7.4	5.4	0.093	≤5.1	6.7	-0.027
50-54	10.0	6.8	0.112	8.5	8.7	0.013
55-59	9.0	8.2	0.029	≤7.8	9.1	-0.037
60-64	10.1	7.5	0.055	≤10.5	8.4	0.039
65-69	8.6	7.8	0.074	≤8.6	7.8	0
70-74	8.4	9.8	-0.037	9.6	8.4	0.058
75-79	≤7.5	11.5	-0.194	≤10.2	10.4	-0.058
80-84	6.1	9.1	-0.117	≤7.3	7.3	-0.027
85-89	≤3.6	4.6	-0.087	≤5.2	≤2.8	0.085
90-94	≤1.2	≤1.5	-0.041	≤2.8	≤0.9	0.094
95-99	≤0.9	≤0.5	-0.003	≤2.8	≤0.6	0.107
Female	62.0	66.2	-0.021	64.3	65.0	-0.007
**Disease**						
Mild depression	31.9	27.5	-0.202	31.7	26.3	-0.03
Recurrent depressive disorder	7.2	3.1	0.175	≤5.3	4.3	0.034
Moderate depression	27.8	18.7	0.144	21.5	19.8	-0.003
Inflammation of specific body systems	28.9	24.1	0.144	26.6	26.4	0.039
Degenerative disease of the central nervous system	9.6	20.2	-0.273	17.7	14.9	0.055
Cognitive disorder	≤3.5	10.2	-0.317	≤7.2	5.3	-0.032
Cerebral degeneration	9.1	20.2	-0.285	17.1	14.9	0.042
Traumatic and/or non-traumatic brain injury	≤1.1	≤0.8	0.017	≤2.0	1.0	0.043
Hyperlipidemia	≤5.7	8.4	-0.156	≤7.9	7.6	-0.029
Alzheimer's dementia	7.9	17.5	-0.276	15.6	12.3	0.07
Cerebral infarction	4.2	5.9	-0.096	≤6.1	5.8	-0.022
Heart disease	≤3.7	4.1	-0.063	≤4.3	3.2	-0.046
**Drug**						
Other ophthalmologicals	38.5	31.1	0.204	33.9	34.5	0.019
Proton pump inhibitors	12.9	16.7	-0.079	13.1	16.1	-0.076
ACE inhibitors	≤0.8	≤0.7	0.023	≤2.0	≤0.7	0.067
Drugs for constipation	19.2	17.4	0.137	19.6	20.0	0.048
Vitamin B1, plain	15.4	11.0	0.211	12.4	14.9	-0.038
Beta blocking agents	≤12.6	9.7	-0.009	≤12.2	11.0	-0.015
Antipruritics, incl. antihistamines, anesthetics, etc.	14.1	12.6	0.076	13.1	14.4	-0.02
Acetaminophen	19.7	16.5	0.036	18.2	17.8	-0.005
Antiepileptics	22.5	19.9	0.02	20.5	23.6	-0.078
Other plain vitamin preparations	12.0	8.7	0.21	10.2	11.8	-0.016
Antianemic preparations	12.6	11.2	0.112	12.6	13.9	-0.02
Statins (HMG CoA reductase inhibitors)	8.6	13.5	-0.166	11.4	12.4	-0.037
Angiotensin II antagonists	≤3.9	5.3	-0.068	≤4.1	5.9	-0.057
Atypical antipsychotics	≤4.3	5.6	-0.045	≤6.3	5.5	0.021
Amlodipine	≤3.4	4.2	-0.048	≤4.2	3.7	-0.001
Aspirin	7.2	9.0	-0.06	10.0	8.8	0.069
Metformin	≤4.2	4.1	-0.072	≤4.8	5.1	-0.076

Included hospitals: EUMC, KDH, KHMC, KWMC, and SCHBC.

Supplementary Table S1k. Gastrointestinal system (Escitalopram vs Trazodone)

Covariate	Before PSM Adjustment			After PSM Adjustment		
	Escitalopram (N=4,029)	Trazodone (N=1,915)	Std. Diff	Escitalopram (N=3,839)	Trazodone (N=933)	Std. Diff
**Age & Sex group**						
18-19	1.9	3.8	-0.015	3.0	5.4	-0.03
20-24	≤6.0	6.6	0.044	≤7.2	≤6.3	0.029
25-29	≤4.1	3.3	0.041	≤4.5	3.8	-0.004
30-34	≤3.4	3.2	0.034	≤4.0	3.6	0.01
35-39	≤4.2	2.8	0.046	≤4.2	3.2	0.032
40-44	≤4.9	3.8	0.105	≤4.6	4.1	0.06
45-49	≤5.8	4.9	0.035	≤5.2	≤5.5	-0.022
50-54	≤9.1	6.4	0.084	≤8.1	8.1	0.009
55-59	≤8.4	7.4	0.067	≤7.2	8.4	0.009
60-64	≤10.1	8.0	0.056	≤9.8	10.0	-0.011
65-69	≤9.3	9.3	-0.019	≤9.7	9.6	-0.022
70-74	≤9.6	11.6	-0.083	≤11.1	9.9	0.013
75-79	9.7	15.1	-0.13	≤11.8	12.6	-0.024
80-84	≤9.2	10.5	-0.069	≤9.4	8.5	-0.02
85-89	≤4.9	5.2	-0.05	≤6.3	3.6	0.062
90-94	≤1.7	1.7	-0.024	≤3.0	≤1.1	0.064
95-99	-	-	-	-	-	-
Female	59.8	64.9	-0.057	63.1	63.1	0.038
**Disease**						
Mild depression	≤39.1	26.8	-0.159	≤34.7	24.7	-0.033
Recurrent depressive disorder	≤8.7	4.8	0.149	≤6.8	7.8	-0.059
Moderate depression	22.0	12.9	0.059	16.0	14.8	-0.025
Inflammation of specific body systems	30.4	28.7	0.063	30.4	31.5	0.035
Degenerative disease of the central nervous system	≤11.4	21.3	-0.295	≤17.4	14.0	-0.01
Cognitive disorder	≤4.5	9.9	-0.217	≤7.7	5.1	0.023
Cerebral degeneration	≤10.9	21.1	-0.303	≤16.9	13.9	-0.019
Traumatic and/or non-traumatic brain injury	≤1.3	≤0.9	0.028	≤2.5	≤1.3	0.076
Hyperlipidemia	≤13.9	16.9	-0.177	≤16.6	14.4	-0.006
Alzheimer's dementia	9.8	21.8	-0.298	16.1	14.0	0.021
Cerebral infarction	≤8.6	13.9	-0.241	≤11.9	10.6	-0.079
Heart disease	≤11.2	11.8	-0.049	≤11.7	10.9	0.047
**Drug**						
Other ophthalmologicals	43.4	39.4	0.102	40.8	44.8	-0.036
Proton pump inhibitors	16.6	21.3	-0.046	17.9	22.4	-0.08
ACE inhibitors	≤1.3	1.8	-0.022	≤2.4	≤1.7	-0.009
Drugs for constipation	20.6	19.4	0.053	20.5	23.7	-0.058
Vitamin B1, plain	17.3	13.1	0.15	16.8	18.7	-0.004
Beta blocking agents	18.0	15.9	0.021	≤15.7	16.3	0
Antipruritics, incl. antihistamines, anesthetics, etc.	17.6	14.4	0.087	16.4	18.6	-0.04
Acetaminophen	23.1	19.4	0.073	20.2	23.7	-0.025
Antiepileptics	23.3	23.4	0.079	23.6	29.5	-0.035
Other plain vitamin preparations	15.3	11.3	0.169	15.4	15.5	0.04
Antianemic preparations	16.5	13.1	0.095	15.1	16.8	-0.025
Statins (HMG CoA reductase inhibitors)	≤16.6	24.4	-0.235	≤19.0	21.6	-0.105
Angiotensin II antagonists	≤13.1	15.9	-0.116	≤13.7	15.9	-0.071
Atypical antipsychotics	≤5.0	4.6	0.046	≤5.8	5.3	0.012
Amlodipine	≤9.6	11.5	-0.081	≤11.4	10.5	0.013
Aspirin	≤13.8	16.1	-0.125	≤15.0	14.6	-0.019
Metformin	≤7.6	8.2	-0.074	≤6.8	8.6	-0.048

Included hospitals: CHNUH, EUMC, ISH, KHMC, KHNMC, KWMC, and SCHBC.

Supplementary Table S1l. Heart failure (Escitalopram vs Trazodone)

Covariate	Before PSM Adjustment			After PSM Adjustment		
	Escitalopram (N=1,805)	Trazodone (N=1,699)	Std. Diff	Escitalopram (N=1,720)	Trazodone (N=687)	Std. Diff
**Age & Sex group**						
18-19	1.4	2.1	-0.055	1.4	2.7	-0.094
20-24	≤4.9	4.7	0.082	≤6.4	5.4	0.055
25-29	≤3.4	2.3	0.028	≤3.4	3.1	-0.007
30-34	≤2.7	2.3	-0.008	≤2.5	3.2	-0.067
35-39	≤4.1	1.9	0.044	≤2.8	2.4	-0.016
40-44	≤4.5	2.5	0.088	≤4.5	≤3.6	0.036
45-49	≤5.3	3.0	0.102	≤3.8	≤3.7	0.055
50-54	8.5	4.7	0.129	7.3	6.8	0.031
55-59	8.5	6.0	0.112	6.1	6.3	0.044
60-64	10.1	7.6	0.068	9.6	8.3	0.031
65-69	8.7	10.1	-0.039	8.8	11.1	-0.071
70-74	9.3	13.6	-0.05	10.6	12.1	-0.016
75-79	11.0	17.7	-0.158	14.0	14.6	-0.035
80-84	10.6	13.3	-0.074	12.9	11.0	0.073
85-89	≤6.0	6.3	-0.041	≤6.8	4.9	-0.003
90-94	≤1.8	≤1.8	-0.012	≤3.2	≤1.1	0.087
95-99	-	-	-	-	-	-
Female	57.4	66.1	-0.122	63.3	61.4	0.049
**Disease**						
Mild depression	≤46.5	33.8	-0.117	≤44.0	31.4	-0.003
Recurrent depressive disorder	8.2	6.6	0.133	6.3	9.3	-0.016
Moderate depression	23.2	15.0	0.023	18.6	18.7	-0.118
Inflammation of specific body systems	34.7	31.0	0.105	32.7	30.8	0.094
Degenerative disease of the central nervous system	13.1	30.1	-0.295	22.4	20.2	0.057
Cognitive disorder	4.6	16.1	-0.282	9.6	8.4	0.011
Cerebral degeneration	12.3	30.0	-0.313	21.8	20.1	0.046
Traumatic and/or non-traumatic brain injury	≤1.0	0.9	-0.01	≤1.8	≤0.8	0.079
Hyperlipidemia	≤16.8	21.2	-0.143	≤19.5	17.4	-0.011
Alzheimer's dementia	10.9	28.7	-0.349	20.6	18.9	0.021
Cerebral infarction	≤9.2	16.3	-0.214	13.4	11.9	0.004
Heart disease	≤9.8	10.9	-0.114	≤8.9	9.9	-0.065
**Drug**						
Other ophthalmologicals	46.5	38.4	0.113	39.7	40.4	-0.006
Proton pump inhibitors	19.4	21.4	-0.055	18.9	19.1	0.004
ACE inhibitors	≤1.1	1.2	-0.013	≤1.3	1.4	-0.014
Drugs for constipation	23.9	19.4	0.076	19.5	22.2	-0.078
Vitamin B1, plain	17.9	11.1	0.148	13.9	12.8	0.049
Beta blocking agents	16.6	15.0	0.042	16.2	15.3	0.016
Antipruritics, incl. antihistamines, anesthetics, etc.	18.8	13.9	0.099	15.7	15.5	0.001
Acetaminophen	26.1	20.7	0.045	22.0	21.4	0.003
Antiepileptics	22.5	17.7	0.142	22.3	23.2	-0.023
Other plain vitamin preparations	15.8	10.6	0.121	12.4	12.0	0.015
Antianemic preparations	18.1	13.2	0.045	14.7	14.3	-0.011
Statins (HMG CoA reductase inhibitors)	17.8	26.1	-0.166	19.9	21.8	-0.043
Angiotensin II antagonists	15.4	17.4	-0.045	15.8	17.6	-0.054
Atypical antipsychotics	3.3	4.2	-0.017	≤3.2	6.0	-0.157
Amlodipine	≤10.6	12.9	-0.092	≤11.2	11.7	-0.041
Aspirin	14.9	16.8	-0.098	16.6	14.3	0.024
Metformin	≤8.2	8.6	-0.06	≤8.4	7.1	0.015

Included hospitals: CHNUH, KHMC, and KWMC.

Supplementary Table S1m. **Hematologic side effects (Escitalopram vs Trazodone)**

Covariate	Before PSM Adjustment			After PSM Adjustment		
	Escitalopram (N=4,106)	Trazodone (N=2,602)	Std. Diff	Escitalopram (N=3,823)	Trazodone (N=1,129)	Std. Diff
**Age & Sex group**						
18-19	≤1.9	≤3.3	-0.033	≤2.7	≤4.2	0.001
20-24	5.1	6.0	-0.004	≤6.5	6.5	-0.036
25-29	≤3.8	3.4	0.052	≤4.4	3.9	0.023
30-34	≤3.5	3.5	0.02	≤3.2	3.8	-0.032
35-39	≤4.1	2.8	0.071	≤4.0	3.1	0.05
40-44	≤5.1	3.5	0.114	≤4.0	3.9	0.051
45-49	≤6.4	4.9	0.041	≤4.8	6.2	-0.089
50-54	≤9.0	6.0	0.092	≤7.3	7.8	-0.038
55-59	8.9	7.8	0.077	≤7.4	8.6	0.019
60-64	9.7	7.7	0.064	9.6	8.5	0.049
65-69	9.1	9.1	0.008	≤10.1	8.5	0.029
70-74	≤9.6	11.7	-0.128	≤9.9	10.3	-0.063
75-79	9.6	13.4	-0.123	≤11.2	11.6	-0.017
80-84	≤8.6	10.5	-0.094	≤9.7	8.8	-0.01
85-89	4.9	5.5	-0.056	6.5	3.7	0.112
90-94	≤1.6	1.7	-0.037	≤2.8	1.2	0.041
95-99	≤0.4	≤0.4	-0.036	≤1.2	≤0.5	0.049
Female	60.7	65.8	-0.04	63.0	64.7	0.001
**Disease**						
Mild depression	34.7	30.8	-0.227	≤33.4	27.9	-0.068
Recurrent depressive disorder	7.0	3.5	0.158	≤5.1	5.3	-0.004
Moderate depression	25.2	16.5	0.108	18.9	17.8	-0.013
Inflammation of specific body systems	33.6	29.6	0.094	31.4	30.8	0.068
Degenerative disease of the central nervous system	11.0	22.8	-0.307	18.5	15.4	0.062
Cognitive disorder	≤3.9	10.4	-0.273	≤7.1	5.3	-0.021
Cerebral degeneration	10.5	22.7	-0.318	18.0	15.2	0.051
Traumatic and/or non-traumatic brain injury	≤1.3	≤1.0	-0.002	≤2.2	≤1.0	0.041
Hyperlipidemia	13.9	16.6	-0.181	16.0	13.2	0.02
Alzheimer's dementia	9.1	19.7	-0.308	16.2	12.7	0.064
Cerebral infarction	≤8.6	12.9	-0.194	≤12.0	10.0	-0.009
Heart disease	≤11.0	11.2	-0.027	≤10.6	9.0	0.07
**Drug**						
Other ophthalmologicals	43.4	37.0	0.119	38.8	39.6	0.007
Proton pump inhibitors	17.3	20.7	-0.059	18.3	18.9	0.002
ACE inhibitors	≤1.3	1.9	-0.035	≤1.8	1.5	-0.007
Drugs for constipation	23.2	21.5	0.083	22.4	23.6	0.031
Vitamin B1, plain	17.9	12.9	0.164	14.7	17.6	-0.02
Beta blocking agents	16.9	14.8	0.012	≤14.3	15.3	-0.03
Antipruritics, incl. antihistamines, anesthetics, etc.	17.1	15.2	0.04	14.6	16.6	-0.041
Acetaminophen	23.5	19.1	0.062	21.6	20.8	0.028
Antiepileptics	24.5	21.1	0.075	21.2	23.3	-0.012
Other plain vitamin preparations	15.5	10.7	0.187	12.6	15.1	-0.007
Antianemic preparations	14.7	12.3	0.073	14.0	16.3	-0.028
Statins (HMG CoA reductase inhibitors)	≤16.9	23.9	-0.236	≤19.2	19.5	-0.043
Angiotensin II antagonists	≤13.0	15.6	-0.125	≤12.9	15.1	-0.065
Atypical antipsychotics	≤4.3	4.9	-0.003	≤5.1	5.3	-0.04
Amlodipine	≤9.7	11.2	-0.061	≤10.5	10.6	-0.008
Aspirin	≤13.5	16.0	-0.146	≤14.7	14.2	-0.028
Metformin	≤7.9	7.9	-0.103	≤7.6	7.9	-0.048

Included hospitals: EUMC, KDH, KHMC, KHNMC, KWMC, and SCHBC.

Supplementary Table S1n. QT prolongation (Escitalopram vs Trazodone)

Covariate	Before PSM Adjustment			After PSM Adjustment		
	Escitalopram (N=2,278)	Trazodone (N=2,023)	Std. Diff	Escitalopram (N=2,136)	Trazodone (N=722)	Std. Diff
**Age & Sex group**						
18-19	1.5	2.3	-0.055	1.7	2.1	-0.018
20-24	3.8	4.7	-0.046	≤4.6	5.0	-0.073
25-29	3.3	3.0	0.023	≤3.5	4.6	-0.054
30-34	≤3.4	3.0	-0.01	≤2.9	3.3	-0.06
35-39	4.5	2.8	0.082	≤4.0	3.1	0.021
40-44	5.1	3.4	0.098	≤3.7	4.2	0.006
45-49	6.6	4.9	0.106	4.6	6.9	-0.068
50-54	9.3	5.4	0.18	8.2	7.0	0.063
55-59	9.6	7.6	0.142	8.2	8.8	0.037
60-64	9.9	7.8	0.073	10.1	8.8	0.058
65-69	8.5	8.9	0.016	8.3	9.2	-0.025
70-74	9.1	10.8	-0.078	9.8	9.9	-0.017
75-79	9.4	13.4	-0.202	12.6	10.5	0.016
80-84	9.1	11.9	-0.119	9.6	10.4	-0.044
85-89	5.2	7.3	-0.12	6.6	4.5	0.09
90-94	≤1.7	2.2	-0.067	≤3.2	1.5	0.042
95-99	≤0.7	≤0.4	-0.021	≤1.9	≤0.5	0.082
Female	60.3	66.5	-0.068	63.7	67.1	-0.048
**Disease**						
Mild depression	40.9	38.1	-0.218	41.7	34.9	-0.013
Recurrent depressive disorder	7.3	3.1	0.183	≤5.4	4.7	-0.008
Moderate depression	28.4	20.4	0.13	21.7	20.6	-0.069
Inflammation of specific body systems	34.9	29.8	0.201	30.6	32.8	0.001
Degenerative disease of the central nervous system	10.6	24.2	-0.343	20.7	16.4	0.097
Cognitive disorder	≤3.9	13.5	-0.373	≤8.7	6.0	0.022
Cerebral degeneration	10.1	24.0	-0.354	20.1	16.2	0.082
Traumatic and/or non-traumatic brain injury	1.0	0.9	0.017	≤1.9	0.7	0.04
Hyperlipidemia	14.2	14.7	-0.115	16.9	12.7	0.072
Alzheimer's dementia	9.1	21.8	-0.342	18.7	13.7	0.118
Cerebral infarction	8.2	11.0	-0.075	11.6	9.6	0.047
Heart disease	9.8	9.5	-0.007	9.4	7.4	0.064
**Drug**						
Other ophthalmologicals	45.3	37.1	0.22	39.3	42.4	-0.079
Proton pump inhibitors	17.0	21.8	-0.107	18.3	18.6	-0.029
ACE inhibitors	≤1.5	2.3	-0.031	≤1.7	1.7	-0.015
Drugs for constipation	23.3	20.8	0.142	20.7	23.6	-0.032
Vitamin B1, plain	19.5	13.8	0.254	15.5	20.2	-0.143
Beta blocking agents	15.6	13.8	-0.004	14.0	13.2	0.005
Antipruritics, incl. antihistamines, anesthetics, etc.	18.3	16.8	0.085	14.8	19.0	-0.08
Acetaminophen	24.9	20.1	0.093	22.7	22.6	-0.003
Antiepileptics	24.7	21.6	0.073	22.7	23.1	0.013
Other plain vitamin preparations	16.5	10.5	0.271	13.0	15.8	-0.107
Antianemic preparations	17.6	13.4	0.15	15.4	17.8	-0.104
Statins (HMG CoA reductase inhibitors)	16.2	22.6	-0.179	19.1	19.3	0.018
Angiotensin II antagonists	13.3	14.5	-0.076	14.5	14.3	0.016
Atypical antipsychotics	≤3.5	4.6	-0.047	≤5.3	4.8	0.059
Amlodipine	9.8	10.6	-0.046	9.7	10.0	-0.002
Aspirin	14.1	17.2	-0.1	16.5	15.2	0.029
Metformin	7.8	7.7	-0.079	≤8.0	7.6	-0.025

Included hospitals: KDH, KWMC, and SCHBC.

Supplementary Table S2. Hospital-Level Inclusion and Exclusion by Outcome (Escitalopram vs. Sertraline)

Tables 5 and 6 list, for each safety outcome, every hospital that contributed a valid propensity-score-matched estimate at analysis (Included) as well as every hospital that attempted the analysis but was excluded (Excluded), together with the specific reason for exclusion: a matched sample size below 30 (Matched N < 30), an individual-hospital 95% confidence interval upper bound exceeding 10 (Upper 95% CI > 10), or a non-convergent/unstable Cox model, typically arising from zero or near-zero event counts in one arm (Unstable model, with target/comparator event counts shown in parentheses). Target N and Comparator N denote the number of matched patients in the escitalopram and comparator (sertraline or trazodone) arms, respectively, at the hospital in question. Effective sample size (ESS) is calculated as 4 × (Target N × Comparator N) / (Target N + Comparator N) [23], and represents the size of a hypothetical 1:1 balanced cohort that would yield the same statistical precision as the observed unequal matching ratio; it therefore quantifies the precision lost when the two arms differ substantially in size. ESS is reported for all hospitals, including those excluded from the pooled analysis.

Escitalopram vs Sertraline							
**Outcome**	**Site**	**Status**	**Target N**	**Comparator N**	**Target events**	**Reason for exclusion**	**Effective sample size**
**Atrial fibrillation**	KWMC	Included	696	1,090	15		1699.1
	DCMC	Included	199	1,205	1		683.2
	SEJONG_ICN	Included	34	261	2		120.3
	AUMC	Excluded	230	991	0	Zero events / masked (events 0/8)	746.7
	KHMC	Excluded	142	655	1	Upper 95% CI > 10 (26.20)	466.8
	KDH	Excluded	121	1,090	5	Upper 95% CI > 10 (51.58)	435.6
	GNUH	Excluded	85	652	0	Unstable model (events 0/0)	300.8
	CAUHS	Excluded	63	173	1	Unstable model (events 1/1)	184.7
	SCHBC	Excluded	51	458	0	Unstable model (events 0/4)	183.6
	SCHSU	Excluded	42	839	2	Upper 95% CI > 10 (35.73)	160.0
	HUMC_DONGTAN	Excluded	25	233	0	Unstable model (events 0/0)	90.3
**Cardiac arrest**	KWMC	Included	727	1,136	9		1773.2
	AUMC	Included	229	984	1		743.1
	DCMC	Included	219	1,307	2		750.3
	KHMC	Excluded	147	752	1	Unstable model (events 1/0)	491.9
	KDH	Excluded	123	1,089	0	Unstable model (events 0/3)	442.1
	GNUH	Excluded	85	650	1	Upper 95% CI > 10 (19.77)	300.7
	KYUH	Excluded	83	666	0	Unstable model (events 0/5)	295.2
	CAUHS	Excluded	66	177	0	Unstable model (events 0/0)	192.3
	PNUH	Excluded	47	91	0	Unstable model (events 0/0)	124.0
	SCHSU	Excluded	43	860	0	Unstable model (events 0/1)	163.8
	SEJONG_ICN	Excluded	39	248	0	Unstable model (events 0/0)	134.8
	HUMC_DONGTAN	Excluded	25	240	1	Unstable model (events 1/0)	90.6
**Circulatory system**	KWMC	Included	498	814	58		1235.9
	AUMC	Included	181	750	14		583.2
	DCMC	Included	147	703	7		486.3
	EUMC	Included	126	1,249	7		457.8
	KHMC	Included	88	380	10		285.8
	UUH	Included	88	1,203	7		328.0
	KDH	Included	83	765	14		299.5
	GNUH	Included	72	556	5		255.0
	KYUH	Included	69	574	6		246.4
	CHNUH	Included	62	450	4		218.0
	CAUHS	Included	51	145	2		150.9
	SCHBC	Included	36	329	5		129.8
	PNUH	Excluded	40	74	2	Upper 95% CI > 10 (14.27)	103.9
	SCHSU	Excluded	26	601	3	Matched N < 30 (=26)	99.7
	ISH	Excluded	23	397	1	Matched N < 30 (=23)	87.0
	HUMC_DONGTAN	Excluded	22	190	0	Unstable model (events 0/15)	78.9
	SEJONG_ICN	Excluded	22	147	2	Matched N < 30	76.5
	DSMC	Excluded	14	58	1	Matched N < 30	45.1
**Gastrointestinal system**	KWMC	Included	580	899	70		1410.2
	AUMC	Included	211	879	5		680.6
	KHMC	Included	140	673	12		463.6
	KDH	Included	91	837	9		328.3
	CAUHS	Included	59	152	6		170.0
	SCHBC	Included	42	384	5		151.4
	SEJONG_ICN	Excluded	26	173	1	Matched N < 30	90.4
	HUMC_DONGTAN	Excluded	24	193	1	Matched N < 30	85.4
	KWMC	Included	695	1,086	11		1695.2

Heart failure	DCMC	Included	187	1,214	1		648.2
	EUMC	Included	144	1,455	1		524.1
	KHMC	Included	140	632	4		458.4
	AUMC	Excluded	218	977	1	Upper 95% CI > 10 (16.18)	712.9
	GNUH	Excluded	83	642	0	Unstable model (events 0/5)	294.0
	KYUH	Excluded	83	665	1	Unstable model (events 1/15)	295.2
	CAUHS	Excluded	62	171	1	Upper 95% CI > 10 (62.47)	182.0
	SCHSU	Excluded	42	845	1	Upper 95% CI > 10 (351.83)	160.0
	PNUH	Excluded	36	69	0	Unstable model (events 0/0)	94.6
	SEJONG_ ICN	Excluded	28	198	0	Zero events / masked (events 0/13)	98.1
	HUMC_ DONGTAN	Excluded	25	230	0	Unstable model (events 0/0)	90.2
	DSMC	Excluded	23	90	0	Unstable model (events 0/2)	73.3
	KHNMC	Excluded	7	32	0	Unstable model (events 0/0)	23.0
Hematologic side effects	KWMC	Included	689	1,073	27		1678.3
	AUMC	Included	218	951	2		709.4
	DCMC	Included	197	1,209	9		677.6
	MJH	Included	175	827	12		577.7
	KHMC	Included	152	699	8		499.4
	EUMC	Included	147	1,451	2		533.9
	KDH	Included	114	1,048	6		411.3
	GNUH	Excluded	85	639	0	Zero events / masked (events 0/17)	300.1
	KYUH	Excluded	76	714	3	Upper 95% CI > 10 (12.70)	274.8
	SCHBC	Excluded	45	465	0	Unstable model (events 0/13)	164.1
	SEJONG_ ICN	Excluded	34	258	1	Upper 95% CI > 10 (29.72)	120.2
	HUMC_ DONGTAN	Excluded	23	237	1	Matched N < 30 (=23)	83.9
Hypersensitivity	AUMC	Included	229	983	2		742.9
	EUMC	Included	150	1,531	1		546.5
	KDH	Included	123	1,062	2		440.9
	KWMC	Excluded	727	1,130	2	Unstable model (events 2/2)	1769.5
	KHMC	Excluded	147	720	0	Unstable model (events 0/8)	488.3
	KYUH	Excluded	83	675	1	Upper 95% CI > 10 (11.31)	295.6
	CAUHS	Excluded	66	176	0	Unstable model (events 0/2)	192.0
	SCHBC	Excluded	53	464	0	Unstable model (events 0/0)	190.3
	SCHSU	Excluded	43	861	0	Unstable model (events 0/5)	163.8
	PNUH	Excluded	39	72	0	Unstable model (events 0/0)	101.2
	SEJONG_ ICN	Excluded	39	240	0	Unstable model (events 0/1)	134.2
	HUMC_ DONGTAN	Excluded	25	240	0	Unstable model (events 0/0)	90.6
Psycho-neurological system	AUMC	Included	184	779	13		**595.4**
	KHMC	Included	145	555	6		459.9
	EUMC	Included	128	1,171	8		461.5
	MJH	Included	122	611	11		406.8
	KDH	Included	73	577	7		259.2
	GNUH	Included	71	508	5		249.2
	KYUH	Included	61	559	6		220.0
	CAUHS	Included	56	132	2		157.3
	SCHBC	Included	37	335	3		133.3
	SEJONG_ ICN	Included	33	151	1		108.3
	SCHSU	Included	31	556	4		117.5
	PNUH	Excluded	41	71	1	Upper 95% CI > 10 (50.53)	104.0
	HUMC_ DONGTAN	Excluded	18	161	0	Unstable model (events 0/10)	64.8
QT prolongation	KWMC	Included	685	1,080	31		1676.6
	DCMC	Included	196	1,182	4		672.5
	MJH	Included	189	871	5		621.2
	GNUH	Included	85	646	2		300.5
	KYUH	Included	84	715	1		300.7
	SEJONG_ ICN	Included	34	254	2		119.9
	EUMC	Excluded	143	1,499	0	Unstable model (events 0/34)	522.2
	KDH	Excluded	120	1,070	5	Upper 95% CI > 10 (23.34)	431.6
	CAUHS	Excluded	62	172	2	Unstable model (events 2/1)	182.3
	PNUH	Excluded	44	66	0	Unstable model (events 0/0)	105.6
	HUMC_ DONGTAN	Excluded	25	234	1	Matched N < 30	90.3
	DSMC	Excluded	20	99	0	Unstable model (events 0/2)	66.6

Supplementary Table S3. Hospital-Level Inclusion and Exclusion by Outcome (Escitalopram vs. Trazodone)

Tables 5 and 6 list, for each safety outcome, every hospital that contributed a valid propensity-score-matched estimate at analysis (Included) as well as every hospital that attempted the analysis but was excluded (Excluded), together with the specific reason for exclusion: a matched sample size below 30 (Matched N < 30), an individual-hospital 95% confidence interval upper bound exceeding 10 (Upper 95% CI > 10), or a non-convergent/unstable Cox model, typically arising from zero or near-zero event counts in one arm (Unstable model, with target/comparator event counts shown in parentheses). Target N and Comparator N denote the number of matched patients in the escitalopram and comparator (sertraline or trazodone) arms, respectively, at the hospital in question. Effective sample size (ESS) is calculated as 4 × (Target N × Comparator N) / (Target N + Comparator N) [23], and represents the size of a hypothetical 1:1 balanced cohort that would yield the same statistical precision as the observed unequal matching ratio; it therefore quantifies the precision lost when the two arms differ substantially in size. ESS is reported for all hospitals, including those excluded from the pooled analysis.

Escitalopram vs Trazodone

Outcome	Site	Status	Target N	Comparator N	Target events	Reason for exclusion	Effective sample size
**Circulatory system**	KWMC	Included	303	535	44		773.8
	EUMC	Included	167	844	13		557.7
	KDH	Included	144	625	21		468.1
	KHMC	Included	95	330	8		295.1
	SCHBC	Included	46	294	1		159.1
	CAUHS	Excluded	65	106	6	Upper 95% CI > 10 (193.79)	161.2
	SEJONG_ICN	Excluded	19	62	4	Matched N < 30	58.2
	HUMC_DONGTAN	Excluded	9	37	1	Unstable model (events 1/3)	29.0
	PNUH	Excluded	<5	24	0	Unstable model (events 0/2)	13.7
**Gastrointestinal system**	KWMC	Included	348	611	36		886.9
	KHMC	Included	180	566	17		546.3
	EUMC	Included	159	864	12		537.1
	SCHBC	Included	82	491	6		281.1
	CHNUH	Included	68	409	3		233.2
	ISH	Included	65	741	3		239.0
	KHNMC	Included	31	157	6		103.6
	GNUH	Excluded	21	457	1	Matched N < 30	80.3
	SCHGM	Excluded	13	53	2	Matched N < 30	41.8
**Heart failure**	KWMC	Included	444	738	8		1108.9
	KHMC	Included	174	576	3		534.5
	CHNUH	Included	69	406	2		235.9
	CAUHS	Excluded	71	144	0	Unstable model (events 0/1)	190.2
	GNUH	Excluded	30	626	0	Unstable model (events 0/5)	114.5
	SEJONG_ICN	Excluded	20	70	2	Matched N < 30	62.2
	PNUH	Excluded	9	36	0	Unstable model (events 0/0)	28.8
**Hematologic side effects**	KWMC	Included	441	733	36		1101.4
	KDH	Included	200	844	9		646.7
	EUMC	Included	190	954	2		633.8
	KHMC	Included	172	590	7		532.7
	SCHBC	Included	87	510	1		297.3
	KHNMC	Included	39	192	1		129.7
	CHNUH	Excluded	71	421	1	Upper 95% CI > 10 (10.10)	243.0
	SEJONG_ICN	Excluded	22	70	0	Unstable model (events 0/1)	67.0
	GNUH	Excluded	20	459	0	Unstable model (events 0/11)	76.7
	PNUH	Excluded	7	26	0	Unstable model (events 0/0)	22.1
**QT prolongation**	KWMC	Included	424	731	12		1073.4
	KDH	Included	211	877	6		680.3
	SCHBC	Included	87	528	3		298.8
	KHMC	Excluded	168	538	3	Upper 95% CI > 10 (10.95)	512.1
	CAUHS	Excluded	76	145	1	Unstable model (events 1/1)	199.5
	CHNUH	Excluded	69	396	0	Unstable model (events 0/9)	235.0

Supplementary Table S4. Pooled Target-to-Comparator Matching Ratio by Safety Outcome

For each safety outcome and drug comparison, the total number of matched patients pooled across all included hospitals in the target (escitalopram) and comparator (sertraline or trazodone) arms, and the resulting comparator-to-target matching ratio (Comparator N ÷ Target N).

Comparison	Outcome	Target N	Comparator N	Matching Ratio (Comparator: Target)
Escitalopram vs Sertraline	Atrial fibrillation	929	2556	2.751
	Cardiac arrest	1175	3427	2.917
	Circulatory system	1501	7918	5.275
	Gastrointestinal system	1123	3824	3.405
	Heart failure	1166	4387	3.762
	Hematologic side effects	1692	7258	4.29
	Hypersensitivity	502	3576	7.124
	Psycho-neurological system	941	5934	6.306
	QT prolongation	1273	4748	3.73
Escitalopram vs Trazodone	Circulatory system	755	2628	3.481
	Gastrointestinal system	933	3839	4.115
	Heart failure	687	1720	2.504
	Hematologic side effects	1129	3823	3.386
	QT prolongation	722	2136	2.958

Supplementary Table S5. Proportion and Identity of Covariates with an Absolute Standardized Mean Difference Greater Than 0.1 After Propensity Score Matching

For each safety outcome and drug comparison, the number and proportion of baseline covariates (from the covariate set reported in Table 1 and Supplementary Table 1a–n) with an absolute standardized mean difference greater than 0.1 after matching, together with the identity and standardized mean difference of each such covariate.

Supplementary Table S5a. Escitalopram vs Sertraline

Outcome (%)	Covariate	Std.diff (After)
Atrial fibrillation (4.2%)	Heart disease	0.136
	Angiotensin II antagonists	0.114
Cardiac arrest (2.1%)	Atypical antipsychotics	0.103
Circulatory system (2.1%)	25-29	0.105
Gastrointestinal system (4.3%)	Heart disease	0.113
	Metformin	0.109
Heart failure (4.2%)	Other ophthalmologicals	0.109
	Heart disease	0.105
Hypersensitivity (8.5%)	20-24	0.122
	90-94	0.12
	Drugs for constipation	0.118
	Heart disease	0.112
Psycho-neurological system (10.6%)	Recurrent depressive disorder	0.124
	Alzheimer's dementia	0.121
	Degenerative disease of the central nervous system	0.116
	Cerebral degeneration	0.116
	50-54	-0.105
QT prolongation (2.1%)	Drugs for constipation	-0.102

Supplementary Table S5b. Escitalopram vs Trazodone

Outcome	Covariate	Std.diff (After)
Circulatory system (2.1%)	95-99	0.107
Gastrointestinal system (2.1%)	Statins (HMG CoA reductase inhibitors)	-0.105
Heart failure (4.3%)	Atypical antipsychotics	-0.157
	Moderate depression	-0.118
Hematologic side effects (2.1%)	85-89	0.112
QT prolongation (8.3%)	Vitamin B1, plain	-0.143
	Alzheimer's dementia	0.118
	Other plain vitamin preparations	-0.107
	Antianemic preparations	-0.104

Supplementary Table S6(a-b). Hospital-Level Matched Sample Sizes, Event Counts, Follow-Up Time, and Incidence Rates by Safety Outcome

For each safety outcome, drug comparison, and hospital: the number of matched patients, event counts, mean follow-up time, and incidence rate (per 1,000 person-years) in the target and comparator arms, together with the hospital-specific hazard ratio (uncalibrated), 95% confidence interval, inclusion status, and reason for exclusion where applicable.

PY, person-years. Incidence rates are expressed per 1,000 person-years. Hospital-level hazard ratios shown in this table are uncalibrated; empirical calibration was applied once to the pooled estimate for each outcome and drug comparison, rather than separately at each hospital.

Supplementary Table S6a. Escitalopram vs Sertraline

Outcome	Site	Target N	Comparator N	Target events	Comparator events	Target, Mean Follow-up (PY)	Comparator, Mean Follow-up (PY)	Target IR (per 1,000 PY)	Comparator IR (per 1,000 PY)	HR	Lower CI	Upper CI	Exclude reason	Status
Atrial fibrillation	DCMC	199	1205	1	20	3.771	4.278	1.333	3.880	0.000	0.000	1.118		Included
	KWMC	696	1090	15	18	3.287	3.465	6.556	4.766	1.886	0.801	4.641		Included
	SEJONG_ICN	34	261	2	13	1.743	2.529	33.741	19.692	1.062	0.147	5.012		Included
	AUMC	230	991	0	8	4.367	4.707	0.000	1.715	0.000	0.000	0.856	target events=0, comparator events=8	Excluded
	CAUHS	63	173	1	1	2.100	1.845	7.559	3.133				target events=1, comparator events=1	Excluded
	GNUH	85	652	0	0	4.096	4.211	0.000	0.000				target events=0, comparator events=0	Excluded
	HUMC_DONGTAN	25	233	0	0	1.970	3.257	0.000	0.000				target events=0, comparator events=0	Excluded
	KDH	121	1090	5	13	4.500	4.306	9.184	2.770	10.227	1.886	51.580	upperCI>10 (CI_ub=51.58)	Excluded
	KHMC	142	655	1	8	3.152	2.181	2.235	5.600	3.301	0.156	26.199	upperCI>10 (CI_ub=26.20)	Excluded
	SCHBC	51	458	0	4	4.162	4.104	0.000	2.128	0.000		9.207	target events=0, comparator events=4	Excluded
	SCHSU	42	839	2	7	3.660	3.655	13.011	2.283	7.191	0.981	35.727	upperCI>10 (CI_ub=35.73)	Excluded
Cardiac arrest	AUMC	229	984	1	6	4.338	3.737	1.007	1.632	0.000	0.000	1.782		Included
	DCMC	219	1307	2	14	3.672	4.236	2.487	2.529	1.420	0.188	7.167		Included
	KWMC	727	1136	9	14	3.304	3.461	3.746	3.561	1.165	0.416	3.057		Included
	CAUHS	66	177	0	0	2.180	1.844	0.000	0.000				target events=0, comparator events=0	Excluded
	GNUH	85	650	1	2	4.096	4.217	2.872	0.730	1.566	0.063	19.774	upperCI>10 (CI_ub=19.77)	Excluded
	HUMC_DONGTAN	25	240	1	0	1.970	3.262	20.307	0.000				target events=1, comparator events=0	Excluded
	KDH	123	1089	0	3	4.579	4.315	0.000	0.638	0.000		10.337	(target events=0, comparator events=3	Excluded
	KHMC	147	752	1	0	3.204	1.921	2.123	0.000				target events=1, comparator events=0	Excluded

	KYUH	83	666	0	5	2.987	3.278	0.000	2.290	0.000		3.271	target events=0, comparator events=5	Excluded
	PNUH	47	91	0	0	3.681	3.737	0.000	0.000				target events=0, comparator events=0	Excluded
	SCHSU	43	860	0	1	3.772	3.634	0.000	0.320	0.000		168.950	target events=0, comparator events=1	Excluded
	SEJONG_ICN	39	248	0	0	1.980	2.535	0.000	0.000				(target events=0, comparator events=0	Excluded
Circulatory system	AUMC	181	750	14	45	4.396	4.933	17.594	12.162	0.703	0.305	1.477		Included
	CAUHS	51	145	2	5	2.081	1.720	18.847	20.054	1.303	0.180	6.721		Included
	CHNUH	62	450	4	39	2.678	2.441	24.091	35.509	0.635	0.181	1.730		Included
	DCMC	147	703	7	93	3.631	3.920	13.115	33.745	0.423	0.143	1.008		Included
	EUMC	126	1249	7	94	2.909	3.429	19.096	21.948	1.317	0.529	2.815		Included
	GNUH	72	556	5	29	3.654	3.974	19.007	13.123	1.335	0.441	3.327		Included
	KDH	83	765	14	77	4.192	3.857	40.242	26.098	1.495	0.669	3.073		Included
	KHMC	88	380	10	44	2.886	1.011	39.369	114.476	1.537	0.553	3.836		Included
	KWMC	498	814	58	105	3.002	3.136	38.799	41.132	1.077	0.711	1.619		Included
	KYUH	69	574	6	56	2.712	3.081	32.063	31.667	1.815	0.571	4.784		Included
	SCHBC	36	329	5	47	3.765	3.701	36.886	38.605	0.965	0.312	2.445		Included
	UUH	88	1203	7	72	3.220	4.356	24.705	13.741	1.845	0.664	4.302		Included
	DSMC	14	58	1	3	2.234	2.542	31.978	20.349	0.836	0.039	7.484	n<30 (matched_n=14)	Excluded
	HUMC_DONGTAN	22	190	0	15	1.731	2.895	0.000	27.274	0.000		2.202	target events=0, comparator events=15	Excluded
	ISH	23	397	1	34	1.970	2.793	22.069	30.668	1.212	0.067	5.902	n<30 (matched_n=23)	Excluded
	PNUH	40	74	2	7	3.493	3.552	14.314	26.633	1.686	0.199	14.271	upperCI>10 (CI_ub=14.27)	Excluded
	SCHSU	26	601	3	60	3.580	3.388	32.231	29.463	1.264	0.305	3.487	n<30 (matched_n=26)	Excluded
	SEJONG_ICN	22	147	2	25	1.589	2.420	57.209	70.278	1.358	0.190	5.871	n<30 (matched_n=22)	Excluded
Gastrointestinal system	AUMC	211	879	5	23	4.278	3.695	5.539	7.082	0.578	0.130	1.837		Included
	CAUHS	59	152	6	12	2.028	1.785	50.149	44.216	1.157	0.382	3.261		Included
	KDH	91	837	9	109	4.213	3.991	23.472	32.633	0.804	0.330	1.762		Included
	KHMC	140	673	12	47	2.917	1.631	29.388	42.829	2.331	1.018	5.069		Included
	KWMC	580	899	70	107	3.040	3.261	39.697	36.493	1.190	0.823	1.716		Included
	SCHBC	42	384	5	32	4.091	3.881	29.099	21.473	1.184	0.318	3.570		Included
	HUMC_DONGTAN	24	193	1	12	1.938	3.146	21.495	19.766	2.105	0.106	14.766	n<30 (matched_n=24)	Excluded
	SEJONG_ICN	26	173	1	30	1.572	2.111	24.472	82.136	0.247	0.014	1.191	n<30 (matched_n=26)	Excluded
Heart failure	DCMC	187	1214	1	13	3.645	4.204	1.467	2.547	0.779	0.037	6.371		Included
	EUMC	144	1455	1	22	2.914	3.536	2.383	4.276	0.862	0.045	4.809		Included
	KHMC	140	632	4	17	3.033	2.255	9.421	11.928	2.046	0.507	6.773		Included
	KWMC	695	1086	11	22	3.332	3.453	4.749	5.866	0.588	0.222	1.421		Included
	AUMC	218	977	1	3	4.318	4.748	1.062	0.647	1.422	0.064	16.178	upperCI>10 (CI_ub=16.18)	Excluded
	CAUHS	62	171	1	1	2.110	1.816	7.642	3.221	2.449	0.096	62.468	upperCI>10 (CI_ub=62.47)	Excluded
	DSMC	23	90	0	2	2.153	2.486	0.000	8.939	0.000		2.229	(target events=0, comparator events=2	Excluded

	GNUH	83	642	0	5	4.154	4.182	0.000	1.862	0.000		9.676	target events=0, comparator events=5	Excluded
	HUMC_DONGTAN	25	230	0	0	1.970	3.198	0.000	0.000				target events=0, comparator events=0	Excluded
	KHNMC	7	32	0	0	3.141	3.577	0.000	0.000				target events=0, comparator events=0	Excluded
	KYUH	83	665	1	15	2.906	3.208	4.145	7.032	0.000		4.049	target events=1, comparator events=15	Excluded
	PNUH	36	69	0	0	4.046	3.758	0.000	0.000				target events=0, comparator events=0	Excluded
	SCHSU	42	845	1	1	3.675	3.622	6.478	0.327	8.718	0.216	351.834	upperCI>10 (CI_ub=351.83)	Excluded
	SEJONG_ICN	28	198	0	13	2.027	2.445	0.000	26.852	0.000	0.000	0.702	target events=0, comparator events=13	Excluded
	AUMC	218	951	2	10	4.226	3.735	2.171	2.816	0.734	0.105	3.237		Included
	DCMC	197	1209	9	58	3.505	4.164	13.035	11.522	1.516	0.620	3.320		Included
	EUMC	147	1451	2	39	2.902	3.536	4.688	7.600	0.643	0.098	2.382		Included
	KDH	114	1048	6	42	4.562	4.230	11.538	9.474	2.387	0.810	6.165		Included
	KHMC	152	699	8	21	3.130	2.055	16.817	14.618	2.989	0.899	9.166		Included
	KWMC	689	1073	27	43	3.169	3.393	12.366	11.811	0.764	0.390	1.450		Included
	MJH	175	827	12	52	4.296	3.494	15.961	17.996	1.165	0.494	2.514		Included
Hematologic side effects	GNUH	85	639	0	17	4.096	4.137	0.000	6.431	0.000	0.000	0.879	target events=0, comparator events=17	Excluded
	HUMC_DONGTAN	23	237	1	6	1.980	3.187	21.959	7.943	10.952	0.395	128.716	n<30 (matched_n=23)	Excluded
	KYUH	76	714	3	27	2.930	3.266	13.473	11.578	2.899	0.511	12.700	upperCI>10 (CI_ub=12.70)	Excluded
	SCHBC	45	465	0	13	4.249	3.932	0.000	7.109	0.000		2.882	target events=0, comparator events=13	Excluded
	SEJONG_ICN	34	258	1	10	1.961	2.527	14.999	15.335	3.519	0.167	29.720	upperCI>10 (CI_ub=29.72)	Excluded
	AUMC	229	983	2	11	4.271	3.726	2.045	3.003	1.219	0.168	5.598		Included
	EUMC	150	1531	1	7	2.859	3.556	2.332	1.286	0.929	0.042	7.914		Included
	KDH	123	1062	2	16	4.593	4.277	3.541	3.522	0.529	0.027	3.349		Included
	CAUHS	66	176	0	2	2.180	1.816	0.000	6.257	0.000		2.229	target events=0, comparator events=2	Excluded
	HUMC_DONGTAN	25	240	0	0	1.970	3.262	0.000	0.000				target events=0, comparator events=0	Excluded
Hypersensitivity	KHMC	147	720	0	8	3.158	2.460	0.000	4.517	0.000		2.859	target events=0, comparator events=8	Excluded
	KWMC	727	1130	2	2	3.286	3.458	0.837	0.512				target events=2, comparator events=2	Excluded
	KYUH	83	675	1	13	2.940	3.285	4.098	5.864	1.979	0.105	11.309	upperCI>10 (CI_ub=11.31)	Excluded
	PNUH	39	72	0	0	3.669	3.746	0.000	0.000				target events=0, comparator events=0	Excluded

	SCHBC	53	464	0	0	4.128	4.112	0.000	0.000				target events=0, comparator events=0	Excluded
	SCHSU	43	861	0	5	3.772	3.614	0.000	1.607	0.000		12.092	target events=0, comparator events=5	Excluded
	SEJONG_ICN	39	240	0	1	1.980	2.564	0.000	1.625				target events=0, comparator events=1	Excluded
Psycho-neurological system	AUMC	184	779	13	44	4.039	3.542	17.494	15.946	0.865	0.398	1.766		Included
	CAUHS	56	132	2	8	2.139	1.824	16.695	33.220	0.944	0.135	4.409		Included
	EUMC	128	1171	8	146	2.803	3.354	22.294	37.170	0.665	0.288	1.338		Included
	GNUH	71	508	5	37	3.866	3.862	18.216	18.860	1.035	0.346	2.526		Included
	KDH	73	577	7	62	3.923	3.886	24.440	27.654	1.024	0.379	2.500		Included
	KHMC	145	555	6	34	2.968	2.543	13.943	24.093	0.784	0.214	2.309		Included
	KYUH	61	559	6	66	2.614	3.062	37.633	38.554	0.833	0.265	2.162		Included
	MJH	122	611	11	51	3.641	3.344	24.765	24.959	1.094	0.455	2.439		Included
	SCHBC	37	335	3	32	3.631	3.789	22.330	25.212	0.281	0.015	1.421		Included
	SCHSU	31	556	4	56	3.544	3.206	36.405	31.416	1.102	0.298	3.137		Included
	SEJONG_ICN	33	151	1	19	1.928	2.356	15.714	53.416	0.234	0.013	1.254		Included
	HUMC_DONGTAN	18	161	0	10	2.025	3.101	0.000	20.030	0.000		33.384	target events=0, comparator events=10	Excluded
	PNUH	41	71	1	2	3.601	3.584	6.773	7.860	2.000	0.079	50.535	upperCI>10 (CI_ub=50.53)	Excluded
QT prolongation	DCMC	196	1182	4	39	3.736	4.276	5.462	7.717	0.971	0.219	3.076		Included
	GNUH	85	646	2	7	4.094	4.173	5.747	2.597	1.333	0.187	5.901		Included
	KWMC	685	1080	31	33	3.251	3.455	13.918	8.844	1.730	0.963	3.145		Included
	KYUH	84	715	1	18	2.994	3.370	3.976	7.470	0.000	0.000	3.881		Included
	MJH	189	871	5	18	4.135	3.479	6.398	5.941	1.690	0.422	5.710		Included
	SEJONG_ICN	34	254	2	14	1.743	2.562	33.741	21.515	1.469	0.202	7.205		Included
	CAUHS	62	172	2	1	2.081	1.854	15.498	3.136				target events=2, comparator events=1	Excluded
	DSMC	20	99	0	2	2.238	2.601	0.000	7.766	0.000		1.613	target events=0, comparator events=2	Excluded
	EUMC	143	1499	0	34	2.898	3.555	0.000	6.380	0.000		1.258	target events=0, comparator events=34	Excluded
	HUMC_DONGTAN	25	234	1	3	1.970	3.197	20.307	4.010	4.899	0.192	124.938	n<30 (matched_n=25)	Excluded
	KDH	120	1070	5	15	4.427	4.297	9.411	3.262	5.839	1.274	23.344	upperCI>10 (CI_ub=23.34)	Excluded
	PNUH	44	66	0	0	3.671	3.631	0.000	0.000				target events=0, comparator events=0	Excluded

Supplementary Table S6b. Escitalopram vs Trazodone

Outcome	Site	Target, N	Comparator, N	Target events	Comparator events	Target, Mean Follow-up (PY)	Comparator, Mean Follow-up (PY)	Target IR (per 1,000 PY)	Comparator IR (per 1,000 PY)	HR	Lower CI	Upper CI	Exclude reason	Status
Circulatory system	EUMC	167	844	13	76	3.291	3.547	23.653	25.386	0.887	0.398	1.825		Included
Circulatory system	KDH	144	625	21	63	3.678	4.004	39.652	25.173	0.965	0.478	1.867		Included
Circulatory system	KHMC	95	330	8	36	2.488	1.147	33.845	95.131	1.951	0.717	4.965		Included
Circulatory system	KWMC	303	535	44	74	3.044	3.200	47.712	43.223	0.884	0.538	1.431		Included
Circulatory system	SCHBC	46	294	1	46	3.360	3.750	6.470	41.724	0.143	0.008	0.714		Included
Circulatory system	CAUHS	65	106	6	1	2.305	1.785	40.043	5.286	9.000	1.208	193.790	upperCI>10 (CI_ub=193.79)	Excluded
Circulatory system	HUMC_DONGTAN	9	37	1	3	4.144	2.860	26.811	28.351	0.000		2.054	target events=1, comparator events=3	Excluded
Circulatory system	PNUH	-5	24	0	2		3.672	0.000	22.695	0.000		4.004	target events=0, comparator events=2	Excluded
Circulatory system	SEJONG_ICN	19	62	4	11	0.915	2.464	230.043	72.018	3.723	0.667	23.086	n<30 (matched_n=19)	Excluded
Gastrointestinal system	CHNUH	68	409	3	11	2.376	2.434	18.567	11.048	2.294	0.433	9.175		Included
Gastrointestinal system	EUMC	159	864	12	82	3.209	3.502	23.518	27.100	1.077	0.480	2.229		Included
Gastrointestinal system	ISH	65	741	3	53	2.934	2.916	15.733	24.532	0.617	0.140	1.913		Included
Gastrointestinal system	KHMC	180	566	17	41	2.987	3.657	31.617	19.807	1.430	0.676	2.965		Included
Gastrointestinal system	KHNMC	31	157	6	16	3.184	3.937	60.797	25.888	1.829	0.518	5.837		Included
Gastrointestinal system	KWMC	348	611	36	67	3.234	3.369	31.989	32.549	0.834	0.464	1.467		Included
Gastrointestinal system	SCHBC	82	491	6	56	3.179	3.599	23.015	31.691	0.737	0.232	1.970		Included
Gastrointestinal system	GNUH	21	457	1	14	3.599	3.988	13.231	7.682	0.972	0.045	6.913	n<30 (matched_n=21)	Excluded
Gastrointestinal system	SCHGM	13	53	2	7	2.110	3.612	72.904	36.564	4.438	0.447	42.213	n<30 (matched_n=13)	Excluded
Heart failure	CHNUH	69	406	2	10	2.464	2.457	11.765	10.024	0.925	0.122	4.794		Included
Heart failure	KHMC	174	576	3	16	2.035	1.310	8.473	21.205	1.279	0.267	4.691		Included
Heart failure	KWMC	444	738	8	21	3.481	3.469	5.176	8.203	0.533	0.144	1.621		Included
Heart failure	CAUHS	71	144	0	1	2.295	1.709	0.000	4.063				target events=0, comparator events=1	Excluded
Heart failure	GNUH	30	626	0	5	3.582	4.184	0.000	1.909	0.000		33.295	target events=0, comparator events=5	Excluded
Heart failure	PNUH	9	36	0	0	4.552	4.351	0.000	0.000				target events=0, comparator events=0	Excluded
Heart failure	SEJONG_ICN	20	70	2	5	1.863	2.764	53.670	25.843	0.000	0.000	2.036	n<30 (matched_n=20)	Excluded
Hematologic side effects	EUMC	190	954	2	27	3.213	3.666	3.276	7.720	0.209	0.011	1.133		Included
Hematologic side effects	KDH	200	844	9	42	4.496	4.183	10.008	11.896	0.856	0.297	2.154		Included
Hematologic side effects	KHMC	172	590	7	15	3.212	1.282	12.670	19.824	1.459	0.489	4.292		Included
Hematologic side effects	KHNMC	39	192	1	13	3.675	4.261	6.978	15.892	0.440	0.023	2.490		Included
Hematologic side effects	KWMC	441	733	36	35	3.174	3.402	25.721	14.037	1.582	0.857	2.966		Included
Hematologic side effects	SCHBC	87	510	1	15	3.375	3.829	3.406	7.682	0.557	0.028	3.546		Included
Hematologic side effects	CHNUH	71	421	1	10	2.381	2.450	5.916	9.696	1.669	0.087	10.095	upperCI>10 (CI_ub=10.10)	Excluded
Hematologic side effects	GNUH	20	459	0	11	3.695	4.246	0.000	5.644	0.000		4.610	target events=0, comparator events=11	Excluded

	PNUH	7	26	0	0	3.987	4.131	0.000	0.000				target events=0, comparator events=0	Excluded
	SEJONG_ICN	22	70	0	1	1.690	2.611	0.000	5.472				target events=0, comparator events=1	Excluded
QT prolongation	KDH	211	877	6	15	4.257	4.270	6.680	4.006	1.703	0.455	5.963		Included
	KWMC	424	731	12	29	3.455	3.499	8.192	11.339	0.469	0.151	1.228		Included
	SCHBC	87	528	3	12	3.319	3.920	10.389	5.798	1.734	0.337	7.097		Included
	CAUHS	76	145	1	1	2.292	1.768	5.741	3.902				target events=1, comparator events=1	Excluded
	CHNUH	69	396	0	9	2.400	2.420	0.000	9.390	0.000		3.394	target events=0, comparator events=9	Excluded
	KHMC	168	538	3	12	2.104	0.771	8.487	28.915	2.079	0.263	10.947	upperCI>10 (CI_ub=10.95)	Excluded

Supplementary Table S7. Concept IDs for Inclusion and Exclusion Criteria

OMOP concept identifiers used to define the depressive-disorder inclusion criterion and the diagnostic exclusion criteria (bipolar disorder, schizophrenia, anxiety disorder, pregnancy-related diagnoses, and sleep disorder) applied to both the treatment pathway and safety analysis cohorts.

Concept Id	Concept Code	Concept Name	Domain	Standard Concept caption
4149320	310495003	Mild depression	Condition	Standard
4336957	87512008	Mild major depression	Condition	Standard
440383	35489007	Depressive disorder	Condition	Standard
442077	197480006	Anxiety disorder	Condition	Standard
436665	13746004	Bipolar disorder	Condition	Standard
435783	58214004	Schizophrenia	Condition	Standard
4307820	83074005	Unplanned pregnancy	Condition	Standard
441919	65147003	Twin pregnancy	Condition	Standard
4180111	429240000	Third trimester pregnancy less than 36 weeks	Condition	Standard
4217975	72892002	Normal pregnancy	Condition	Standard
444094	118185001	Finding related to pregnancy	Condition	Standard
374905	270487001	Non-organic sleep disorder	Condition	Standard
435524	39898005	Sleep disorder	Condition	Standard

Supplementary Table S8. Hospital Sites and Their Abbreviations

Abbreviations	Hospital name
AUMC	AJOU UNIVERSITY HOSPITAL
CAUHS	CHUNG-ANG UNIVERSITY HOSPITAL
CHNUH	CHUNGNAM NATIONAL UNIVERSITY HOSPITAL
DCMC	DAEGU CATHOLIC UNIV. MEDICAL CENTER
DSMC	KEIMUNG UNIVERSITY DONGSAN HOSPITAL
EUMC	EWHA WOMANS UNIVERSITY MEDICAL CENTER
GNUH	GYEONGSANG NATIONAL UNIVERSITY HOSPITAL
HUMC_DONGTAN	HALLYM UNIVERSITY DONGTAN SACRED HEART HOSPITAL
ISH	INTL. ST. MARY’S HOSPITAL, CKU
KDH	KANGDONG SACRED HEART HOSPITAL
KHMC	KYUNG HEE UNIVERSITY MEDICAL CENTER
KHNMC	KYUNG HEE UNIVERSITY HOSPITAL AT GANGDONG
KWMC	KANGWON NATIONAL UNIVERSITY HOSPITAL
KYUH	KONYANG UNIVERSITY HOSPITAL
MJH	MYONGJI HOSPITAL
PNUH	PUSAN NATIONAL UNIVERSITY HOSPITAL
SCHBC	SOON CHUN HYANG UNIVERSITY HOSPITAL BUCHEON
SCHGM	SOON CHUN HYANG UNIVERSITY HOSPITAL GUMI
SCHSU	SOON CHUN HYANG UNIVERSITY HOSPITAL SEOUL
SEJONG_ICN	INCHEON SEJONG HOSPITAL
UUH	ULSAN UNIVERSITY HOSPITAL

Supplementary Table S9(a-e). Sex and Age Distribution of the Safety Analysis Cohort, by Hospital

Sex and age distribution of patients in the safety analysis cohort, by hospital. Percentages are calculated within each hospital and sex/age category separately (i.e., the sex rows sum to 100% and the age rows sum to 100% for each hospital). Hospitals are split across five sub-tables (9a-9e) for page-width; together they cover all 18 hospitals contributing to the safety analysis.

Supplementary Table S9a

		AUMC		CAUHS		CHNUH		DCMC	
Sex	Male	1,103	33.75%	251	32.85%	422	33.18%	1,189	35.44%
	Female	2,165	66.25%	513	67.15%	850	66.82%	2,166	64.56%
	Total	3,268		764		1,272		3,355	
Age	15 - 19	102	3.12%	18	2.36%	14	1.10%	37	1.10%
	20 - 24	204	6.24%	37	4.84%	29	2.28%	96	2.86%
	25 - 29	151	4.62%	33	4.32%	20	1.57%	57	1.70%
	30 - 34	173	5.29%	29	3.80%	15	1.18%	45	1.34%
	35 - 39	222	6.79%	17	2.23%	20	1.57%	88	2.62%
	40 - 44	304	9.30%	30	3.93%	25	1.97%	118	3.52%
	45 - 49	275	8.41%	36	4.71%	32	2.52%	194	5.78%
	50 - 54	312	9.55%	36	4.71%	50	3.93%	245	7.30%
	55 - 59	281	8.60%	50	6.54%	65	5.11%	303	9.03%
	60 - 64	316	9.67%	72	9.42%	105	8.25%	338	10.07%
	65 - 69	288	8.81%	87	11.39%	181	14.23%	378	11.27%
	70 - 74	239	7.31%	72	9.42%	226	17.77%	487	14.52%
	75 - 79	222	6.79%	86	11.26%	229	18.00%	479	14.28%
	80 - 84	126	3.86%	78	10.21%	189	14.86%	343	10.22%
	85 - 89	43	1.32%	62	8.12%	61	4.80%	126	3.76%
	90 - 94	-	-	17	2.23%	-	-	-	-

Supplementary Table S9b

		DSMC		EUMC		GNUH		HUMC_DONGTAN	
Sex	Male	258	38.62%	1,081	33.94%	461	38.19%	208	33.66%
	Female	410	61.38%	2,104	66.06%	746	61.81%	410	66.34%
	Total	668		3,185		1,207		618	
Age	15 - 19	-	-	130	4.08%	23	1.91%	10	1.62%
	20 - 24	36	5.39%	285	8.95%	72	5.97%	33	5.34%
	25 - 29	21	3.14%	180	5.65%	21	1.74%	21	3.40%
	30 - 34	25	3.74%	118	3.70%	25	2.07%	23	3.72%
	35 - 39	10	1.50%	127	3.99%	27	2.24%	29	4.69%
	40 - 44	19	2.84%	176	5.53%	51	4.23%	48	7.77%
	45 - 49	31	4.64%	179	5.62%	47	3.89%	49	7.93%
	50 - 54	36	5.39%	234	7.35%	92	7.62%	46	7.44%
	55 - 59	53	7.93%	240	7.54%	122	10.11%	56	9.06%
	60 - 64	71	10.63%	233	7.32%	129	10.69%	63	10.19%
	65 - 69	77	11.53%	285	8.95%	171	14.17%	64	10.36%
	70 - 74	88	13.17%	302	9.48%	141	11.68%	51	8.25%
	75 - 79	89	13.32%	322	10.11%	152	12.59%	67	10.84%
	80 - 84	74	11.08%	237	7.44%	102	8.45%	31	5.02%
	85 - 89	24	3.59%	100	3.14%	26	2.15%	22	3.56%
	90 - 94	7	1.05%	35	1.10%	-	-	-	-

Supplementary Table S9c											
		ISH		KDH		KHMC		KHNMC		KWMC	
Sex	Male	393	36.52%	1,235	31.45%	476	34.17%	196	29.47%	1,865	38.68%
	Female	683	63.48%	2,692	68.55%	917	65.83%	469	70.53%	2,956	61.32%
	Total	1,076		3,927		1,393		665		4,821	
Age	15 - 19	27	2.51%	-	-	35	2.51%	13	1.95%	90	1.87%
	20 - 24	87	8.09%	148	3.77%	83	5.96%	64	9.62%	232	4.81%
	25 - 29	57	5.30%	127	3.23%	46	3.30%	31	4.66%	165	3.42%
	30 - 34	46	4.28%	157	4.00%	38	2.73%	37	5.56%	167	3.46%
	35 - 39	50	4.65%	156	3.97%	27	1.94%	31	4.66%	183	3.80%
	40 - 44	68	6.32%	194	4.94%	42	3.02%	47	7.07%	210	4.36%
	45 - 49	66	6.13%	264	6.72%	51	3.66%	75	11.28%	218	4.52%
	50 - 54	105	9.76%	335	8.53%	87	6.25%	67	10.08%	345	7.16%
	55 - 59	117	10.87%	389	9.91%	112	8.04%	62	9.32%	376	7.80%
	60 - 64	110	10.22%	394	10.03%	127	9.12%	53	7.97%	387	8.03%
	65 - 69	72	6.69%	371	9.45%	132	9.48%	56	8.42%	436	9.04%
	70 - 74	75	6.97%	412	10.49%	204	14.64%	56	8.42%	454	9.42%
	75 - 79	83	7.71%	394	10.03%	211	15.15%	51	7.67%	589	12.22%
	80 - 84	80	7.43%	298	7.59%	141	10.12%	14	2.11%	552	11.45%
	85 - 89	26	2.42%	184	4.69%	41	2.94%	-	-	301	6.24%
	90 - 94	-	-	55	1.40%	14	1.01%	-	-	102	2.12%

Supplementary Table S9d									
		KYUH		MJH		PNUH		SCHBC	
Sex	Male	776	38.02%	2,187	37.57%	163	39.28%	554	34.43%
	Female	1,265	61.98%	3,634	62.43%	252	60.72%	1,055	65.57%
	Total	2,041		5,821		415		1,609	
Age	15 - 19	28	1.37%	103	1.77%	5	1.20%	26	1.62%
	20 - 24	143	7.01%	198	3.40%	23	5.54%	49	3.05%
	25 - 29	83	4.07%	196	3.37%	11	2.65%	42	2.61%
	30 - 34	67	3.28%	226	3.88%	13	3.13%	29	1.80%
	35 - 39	97	4.75%	289	4.96%	15	3.61%	45	2.80%
	40 - 44	125	6.12%	374	6.43%	15	3.61%	62	3.85%
	45 - 49	149	7.30%	454	7.80%	17	4.10%	116	7.21%
	50 - 54	153	7.50%	537	9.23%	28	6.75%	132	8.20%
	55 - 59	184	9.02%	526	9.04%	25	6.02%	148	9.20%
	60 - 64	200	9.80%	552	9.48%	41	9.88%	160	9.94%
	65 - 69	199	9.75%	498	8.56%	43	10.36%	159	9.88%
	70 - 74	187	9.16%	456	7.83%	79	19.04%	171	10.63%
	75 - 79	234	11.46%	547	9.40%	48	11.57%	221	13.74%
	80 - 84	136	6.66%	465	7.99%	36	8.67%	148	9.20%
	85 - 89	47	2.30%	285	4.90%	12	2.89%	81	5.03%
	90 - 94	-	-	98	1.68%	-	-	20	1.24%

Supplementary Table S9e									
		SCHGM		SCHSU		SEJONG_ICN		UUH	
Sex	Male	290	32.22%	820	37.00%	257	33.55%	1,177	35.62%
	Female	610	67.78%	1,396	63.00%	509	66.45%	2,127	64.38%
	Total	900		2,216		766		3,304	
Age	15 - 19	14	1.56%	32	1.44%	8	1.04%	57	1.73%
	20 - 24	26	2.89%	80	3.61%	28	3.66%	117	3.54%
	25 - 29	28	3.11%	71	3.20%	16	2.09%	89	2.69%
	30 - 34	44	4.89%	66	2.98%	19	2.48%	129	3.90%
	35 - 39	48	5.33%	92	4.15%	28	3.66%	160	4.84%
	40 - 44	67	7.44%	117	5.28%	26	3.39%	227	6.87%
	45 - 49	75	8.33%	137	6.18%	34	4.44%	315	9.53%
	50 - 54	49	5.44%	191	8.62%	50	6.53%	402	12.17%
	55 - 59	61	6.78%	206	9.30%	69	9.01%	452	13.68%
	60 - 64	73	8.11%	209	9.43%	97	12.66%	391	11.83%
	65 - 69	78	8.67%	206	9.30%	74	9.66%	341	10.32%
	70 - 74	88	9.78%	242	10.92%	61	7.96%	268	8.11%
	75 - 79	103	11.44%	236	10.65%	85	11.10%	229	6.93%
	80 - 84	89	9.89%	182	8.21%	104	13.58%	100	3.03%
	85 - 89	40	4.44%	111	5.01%	52	6.79%	-	-
	90 - 94	16	1.78%	35	1.58%	14	1.83%	-	-

Supplementary Table S10. Sensitivity of Standardized Mean Differences to Masked-Cell Value Assumptions

For each safety outcome and drug comparison, the number of covariate cells masked because of a matched sample count below 5, and the range in the resulting absolute standardized mean difference (in percentage points) across two assumptions for the true value of each masked cell: 0 (min0) and 4 (upper4, the maximum value consistent with a count below 5). The maximum and mean range across all covariates for each outcome are shown, together with the number of covariates for which this range exceeded 1 percentage point.

Comparison	Outcome	Masked Cells (n)	Max Range (pp)	Cells with Range > 1pp (n)
Escitalopram vs. Sertraline	Atrial fibrillation	8	2.0	1
	Cardiac arrest	8	0.7	0
	Circulatory system	39	2.4	1
	Gastrointestinal system	25	1.2	1
	Heart failure	11	1.7	1
	Hematologic side effects	17	1.3	1
	Hypersensitivity	13	2.9	1
	Psycho-neurological system	39	4.0	1
	QT prolongation	23	1.0	0
Escitalopram vs. Trazodone	Circulatory system	27	2.8	1
	Gastrointestinal system	33	2.4	1
	Heart failure	17	1.3	1
	Hematologic side effects	30	1.8	1
	QT prolongation	14	1.9	1

Supplementary Table S11. Concept IDs for Safety Outcomes

OMOP concept identifiers defining each of the nine broad safety outcome categories. Several categories (Hematologic side effects, Circulatory system, Gastrointestinal system, and Psycho-neurological system) are composite phenotypes combining multiple standard concepts, as listed; the remaining categories (Atrial fibrillation, Cardiac arrest, Heart failure, Hypersensitivity, QT prolongation) are defined by a single concept or concept classification.

	Concept Id	Concept Name	Domain	Standard Concept caption
Atrial fibrillation	313217	Atrial fibrillation	Condition	Standard
Hematologic side effects	440689	Agranulocytosis	Condition	Standard
	439777	Anemia	Condition	Standard
	435224	Leukopenia	Condition	Standard
	606834	Leukopenia caused by drug	Condition	Standard
Cardiac arrest	321042	Cardiac arrest	Condition	Standard
Circulatory system	4169095	Bradycardia	Condition	Standard
	4120275	Drug-induced hypotension	Condition	Standard
	320128	Essential hypertension	Condition	Standard
	319041	Orthostatic hypotension	Condition	Standard
	315078	Palpitations	Condition	Standard
	135360	Syncope	Condition	Standard
	444070	Tachycardia	Condition	Standard
Gastrointestinal system	200219	Abdominal pain	Condition	Standard
	436675	Anorexia nervosa	Condition	Standard
	75860	Constipation	Condition	Standard
	80141	Functional diarrhea	Condition	Standard
	42890914	Hydro dipsomania	Condition	Classification
	4057826	Irritable bowel syndrome with diarrhea	Condition	Standard
	4104142	Localized abdominal distention	Condition	Standard
	27674	Nausea and vomiting	Condition	Standard
	36715842	Non-infective neonatal diarrhea	Condition	Standard
	36310382	Parageusia	Condition	Standard
Heart failure	316139	Heart failure	Condition	Standard
Hypersensitivity	135618	Pruritic rash	Condition	Standard
Psycho-neurological system	36714194	Acquired dysarthria	Condition	Standard
	432590	Delusional disorder	Condition	Standard
	4108537	Disorder of excessive somnolence	Condition	Standard
	433316	Dizziness and giddiness	Condition	Standard
	433031	Hallucinations	Condition	Standard
	378253	Headache	Condition	Standard
	436962	Insomnia	Condition	Standard
	500002101	OMOP Suicidality 1	Condition	Classification
	4168212	Restlessness and agitation	Condition	Standard
	4280369	Satyriasis	Condition	Non-Standard
	443782	Tremor	Condition	Standard
QT prolongation	500001801	OMOP Qt Prolongation/Torsade De Pointes 1	Condition	Classification

Supplementary Table S12. Concept IDs for Antidepressants and Other Medications

OMOP standard concept identifiers used to define exposure to antidepressants, mood stabilizers, and antipsychotics considered in the treatment pathway analysis.

Concept Id	Concept Code	Concept Name	Domain	Standard Concept caption
715939	321988	escitalopram	Drug	Standard
739138	36437	sertraline	Drug	Standard
755695	4493	fluoxetine	Drug	Standard
751412	42355	fluvoxamine	Drug	Standard
722031	32937	paroxetine	Drug	Standard
44507700	1455099	vortioxetine	Drug	Standard
715259	72625	duloxetine	Drug	Standard
19080226	588250	milnacipran	Drug	Standard
743670	39786	venlafaxine	Drug	Standard
717607	734064	desvenlafaxine	Drug	Standard
710062	704	amitriptyline	Drug	Standard
778268	5691	imipramine	Drug	Standard
721724	7531	nortriptyline	Drug	Standard
703547	10737	trazodone	Drug	Standard
725131	15996	mirtazapine	Drug	Standard
750982	42347	bupropion	Drug	Standard
19041910	38252	tianeptine	Drug	Standard
36878783	OMOP997499	Agomelatine	Drug	Standard
751246	42351	lithium carbonate	Drug	Standard
757688	89013	aripiprazole	Drug	Standard
785788	61381	olanzapine	Drug	Standard
735979	35636	risperidone	Drug	Standard
766814	51272	quetiapine	Drug	Standard

Supplementary Table S13. Hospitals with Inaccessible Covariate Result

For each safety outcome and drug comparison, the hospitals whose covariate-balance results became inaccessible following changes to institutional data-governance protocols and could not be independently re-verified.

Comparison	Outcome	Site
Escitalopram vs Sertraline	Atrial fibrillation	CAUHS
		SEJONG_ICN
	Cardiac arrest	CAUHS
		GNUH
		PNUH
		SEJONG_ICN
	Circulatory system	CAUHS
		GNUH
		HUMC_DONGTAN
		PNUH
		SEJONG_ICN
	Gastrointestinal system	CAUHS
		HUMC_DONGTAN
		SEJONG_ICN
	Heart failure	CAUHS
		SEJONG_ICN
	Hematologic side effects	GNUH
		SEJONG_ICN
	Hypersensitivity	CAUHS
		SEJONG_ICN
	Psycho-neurological system	CAUHS
		GNUH
		PNUH
		SEJONG_ICN
	QT prolongation	CAUHS
		GNUH
		SEJONG_ICN
Escitalopram vs Trazodone	Circulatory system	SEJONG_ICN
	Heart failure	SEJONG_ICN
	Hematologic side effects	SEJONG_ICN

Supplementary Table S14. Negative Control Outcome Concept IDs

OMOP concept identifiers for the negative control outcomes used in the empirical calibration described in the Methods.

OMOP Concept ID	Outcome Name	OMOP Concept ID	Outcome Name
443698	Abnormal anal Papanicolaou smear	433071	Contusion of multiple sites
443585	Abrasion and/or friction burn of multiple sites	42537730	Coronary artery graft present
4170145	Absence of lung	201606	Crohn's disease
4093531	Absence of toe	75389	Current tear of lateral cartilage AND/OR meniscus of knee
4092879	Absent kidney	80242	Current tear of medial cartilage AND/OR meniscus of knee
42539582	Acquired absence of genital organ	73575	Deformity of toe
380818	Acquired deformity of head	436233	Delayed milestone
31668	Acquired deformity of neck	436906	Disease caused by rickettsiae
4319325	Acquired deformity of trunk	4135080	Dislocation of radial head
432411	Acquired equinus deformity of foot	438021	Disorder due to and following fracture of upper limb
439673	Acute hepatitis B with delta-agent (coinfection) without hepatic coma	192367	Dysplasia of cervix
441481	Adult victim of abuse	78834	Effusion of joint of hand
4218106	Alcoholism	4247710	Effusion of joint of pelvic region
4303805	Allergic reaction to bite and/or sting	72407	Effusion of joint of shoulder region
4101660	Amputated below knee	4062791	Endocrine, nutritional and metabolic disease complicating pregnancy, childbirth and puerperium
4198962	Amputated thumb	200775	Endometrial hyperplasia
4171556	Ankle ulcer	4150043	Epididymitis
77650	Aseptic necrosis of bone	374358	Excess skin of eyelid
439237	Assault	197607	Excessive and frequent menstruation
434170	Atypical squamous cells of undetermined significance on cervical Papanicolaou smear	4059015	Falls
141797	Black piedra	4264617	Foot-drop
79232	Burn of ankle	374801	Foreign body in ear
4067069	Callosity	4131595	Fracture of radius
4172458	Candidiasis of skin	441487	Frostbite
42709838	Cellulitis of lower limb	40481632	Ganglion cyst
4213540	Cervical somatic dysfunction	4201388	Gastrostomy present
443570	Cervicovaginal cytology: Low grade squamous intraepithelial lesion	4166231	Genetic predisposition
201613	Chronic nonalcoholic liver disease	74855	Genital herpes simplex
439674	Chronic viral hepatitis B without delta-agent	437744	Heat exhaustion
4047787	Colles' fracture	440021	Herpes simplex without complication
134734	Compartment syndrome	437489	Herpes zoster with complication
43021250	Complication associated with orthopedic device	440329	Herpes zoster without complication
46269889	Complication due to Crohn's disease	435511	Hypercalcemia
72995	Contracture of joint of hand	4295287	Hypercoagulability state
80492	Contracture of knee joint	77364	Hypermobility of coccyx
439666	Contracture of multiple joints	74731	Hypertrophic osteoarthropathy
199978	Contusion of lower limb	440129	Hypertrophy of nasal turbinates

196473	Hypertrophy of uterus	4129404	Open wound of upper arm
440072	Hypogammaglobulinemia	438130	Harmful pattern of use of opioid
443447	Iatrogenic hypotension	438120	Opioid dependence
4344500	Impingement syndrome of shoulder region	4171915	Orchitis
441417	Incoordination	315361	Orthopnea
434872	Infection by Trichomonas	74080	Orthostatic proteinuria
4057662	Infestation by Phthirus	75920	Osteitis condensans
440053	Infestation by insect	437359	Osteochondritis dissecans
4168222	Intra-abdominal and pelvic swelling, mass and lump	378160	Otorrhea
196168	Irregular periods	77356	Pathological dislocation of joint
72994	Jaccoud's syndrome	4022076	Patient dependence on care provider
78512	Joint contracture of the ankle and/or foot	375292	Perforation of tympanic membrane
78228	Joint derangement	4141640	Perimenopausal disorder
77072	Joint effusion of ankle AND/OR foot	437092	Physiological development failure
72404	Joint stiffness	253796	Pneumothorax
435903	Juvenile osteochondrosis of foot	4012231	Poor stream of urine
438527	Juvenile osteochondrosis of lower extremity, excluding foot	4295261	Postmenopausal state
435633	Juvenile osteochondrosis of upper extremity	4094448	Pregnancy test negative
4115991	Knee joint effusion	198715	Premature menopause
435516	Lipoprotein deficiency disorder	46286594	Problem related to lifestyle
4297984	Local infection of wound	199876	Prolapse of female genital organs
440638	Lyme disease	4295888	Prolapse of intestine
438067	Malaria	194997	Prostatitis
438297	Mechanical complication of cardiac device, implant AND/OR graft	443274	Psychostimulant dependence
432798	Mechanical complication of internal orthopedic device, implant AND/OR graft	4245252	Prostate specific antigen above reference range
439795	Minimal cognitive impairment	436246	Reduced libido
137967	Muscle, ligament and fascia disorders	43021237	Secondary erectile dysfunction
4271024	Musculoskeletal fibromatosis	4052226	Sequelae of injuries of lower limb
4209423	Nicotine dependence	4125590	Slurred speech
201792	Nongonococcal urethritis	36713918	Somatic dysfunction of lumbar region
40480893	Nonspecific tuberculin test reaction	4002818	Spasm of back muscles
72413	Nontraumatic rupture of muscle	4345332	Spinal instability
4215978	Onychomycosis	4008710	Stenosis due to any device, implant AND/OR graft
140648	Onychomycosis due to dermatophyte	4195698	Tenosynovitis
4129408	Open wound of ankle	4339088	Testicular mass
4053600	Open wound of elbow	80946	Tinea manus
77139	Open wound of finger without complication	4163280	Tinea of perianal region
444426	Open wound of foot except toes without complication	133141	Tinea pedis
137426	Open wound of forearm without complication	440268	Toxic effect of carbon monoxide
77421	Open wound of hand except fingers without complication	4201387	Tracheostomy present

4051004	Open wound of scalp	81930	Transient arthropathy
42538119	Transplanted heart valve present	261599	Vocal cord paralysis
74719	Ulcer of foot	195603	Vulval and/or perineal noninflammatory disorders
443593	Ulcer of thigh	132834	White piedra
4092565	Uterine prolapse	4216670	Worried well
435131	Victim of neglect	435723	Wound seroma
444074	Victim of vehicular AND/OR traffic accident		

Supplementary Table S15. Number of Negative Control Outcomes with Estimable Results, by Hospital

For each drug comparison and hospital, the number of the 167 prespecified negative control outcomes for which a hazard ratio and standard error could be estimated (i.e., met the same hospital-level stability criteria applied to the safety outcomes: ≥30 matched patients and an upper 95% confidence limit ≤10) and were therefore used to construct the empirical null distribution for calibration.

Supplementary Table S15a. Escitalopram vs Sertraline

Outcome	Site	Total Negative Control	Negative Controls with Estimable Results
Atrial fibrillation	DCMC	167	24
	KWMC	167	21
	SEJONG_ICN	167	1
Cardiac arrest	AUMC	167	13
	DCMC	167	25
	KWMC	167	21
Circulatory system	AUMC	167	11
	CAUHS	167	1
	CHNUH	167	7
	DCMC	167	14
	EUMC	167	10
	GNUH	167	0
	KDH	167	11
	KHMC	167	9
	KWMC	167	13
	KYUH	167	8
	SCHBC	167	3
	UUH	167	7
Gastrointestinal system	AUMC	167	17
	CAUHS	167	1
	KDH	167	11
	KHMC	167	11
	KWMC	167	17
	SCHBC	167	4
Heart failure	DCMC	167	25
	EUMC	167	12
	KHMC	167	11
	KWMC	167	19
Hematologic side effects	AUMC	167	18
	DCMC	167	21
	EUMC	167	10
	KDH	167	14
	KHMC	167	11
	KWMC	167	20
	MJH	167	15
Hypersensitivity	AUMC	167	16
	EUMC	167	11
	KDH	167	14
Psycho-neurological system	AUMC	167	10
	CAUHS	167	0

	EUMC	167	10
	GNUH	167	2
	KDH	167	11
	KHMC	167	12
	KYUH	167	5
	MJH	167	12
	SCHBC	167	4
	SCHSU	167	6
	SEJONG_ICN	167	4
QT prolongation	DCMC	167	22
	GNUH	167	2
	KWMC	167	21
	KYUH	167	15
	MJH	167	15
	SEJONG_ICN	167	1

Supplementary Table S15b. Escitalopram vs Trazodone

Outcome	Site	Total Negative Control	Negative Controls with Estimable Results
Circulatory system	EUMC	167	11
	KDH	167	16
	KHMC	167	8
	KWMC	167	13
	SCHBC	167	3
Gastrointestinal system	CHNUH	167	9
	EUMC	167	13
	ISH	167	13
	KHMC	167	10
	KHNMC	167	0
	KWMC	167	15
	SCHBC	167	10
Heart failure	CHNUH	167	6
	KHMC	167	13
	KWMC	167	15
Hematologic side effects	EUMC	167	14
	KDH	167	21
	KHMC	167	13
	KHNMC	167	2
	KWMC	167	13
	SCHBC	167	11
QT prolongation	KDH	167	24
	KWMC	167	11
	SCHBC	167	9

Supplementary Table S16(a-b). Uncalibrated and Calibrated Hazard Ratios, 95% Confidence Intervals, and P-values for All Safety Outcomes

Pooled hazard ratios with 95% confidence intervals and P-values before and after empirical calibration using the 167 prespecified negative control outcomes for each of the nine safety outcomes.

Supplementary Table S16a. Escitalopram vs Sertraline

Outcome	Uncalibrated				Calibrated			
	HR	Lower CI	Upper CI	P	HR	Lower CI	Upper CI	P
Atrial fibrillation	1.660	0.756	3.645	0.206	1.513	0.689	3.322	0.302
Cardiac arrest	1.200	0.501	2.876	0.682	1.220	0.509	2.923	0.656
Circulatory system	1.101	0.869	1.395	0.425	1.042	0.822	1.320	0.736
Gastrointestinal system	1.195	0.901	1.585	0.218	1.263	0.952	1.675	0.105
Heart failure	0.886	0.443	1.769	0.731	0.846	0.423	1.689	0.635
Hematologic side effects	1.255	0.837	1.881	0.272	1.304	0.870	1.956	0.198
Hypersensitivity	0.917	0.264	3.192	0.892	0.832	0.239	2.896	0.773
Psycho-neurological system	0.856	0.625	1.172	0.331	0.766	0.559	1.049	0.097
QT prolongation	1.553	0.977	2.467	0.062	1.752	1.103	2.784	0.018

Supplementary Table S16b. Escitalopram vs Trazodone

Outcome	Uncalibrated				Calibrated			
	HR	Lower CI	Upper CI	P	HR	Lower CI	Upper CI	P
Circulatory system	0.957	0.651	1.406	0.821	1.164	0.792	1.711	0.440
Gastrointestinal system	1.051	0.755	1.464	0.769	1.288	0.925	1.795	0.134
Heart failure	0.797	0.349	1.819	0.590	0.990	0.434	2.260	0.981
Hematologic side effects	1.175	0.752	1.838	0.479	1.068	0.683	1.670	0.773
QT prolongation	1.001	0.401	2.498	0.998	0.935	0.375	2.333	0.885

Supplementary Figure S1(a-e). Full-Size Sunburst Plots of Antidepressant Treatment Pathways

Full-size versions of the sunburst plots shown in Figure 1, provided to facilitate detailed inspection of the treatment-pathway hierarchy at each hospital. Each plot illustrates antidepressant treatment pathways from STEP 1 to STEP 3, with the inner ring representing the initial antidepressant therapy (STEP 1) and outer rings representing subsequent treatment pathways (STEP 2 and 3); colors denote individual antidepressants. (a) AUMC, (b) EUMC, (c) KDH, (d) KHNMC, and (e) KWMC.

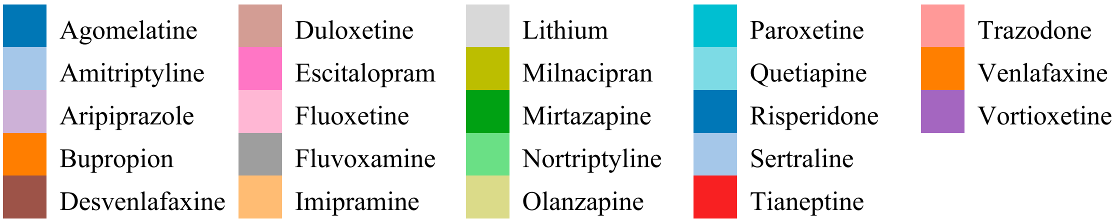

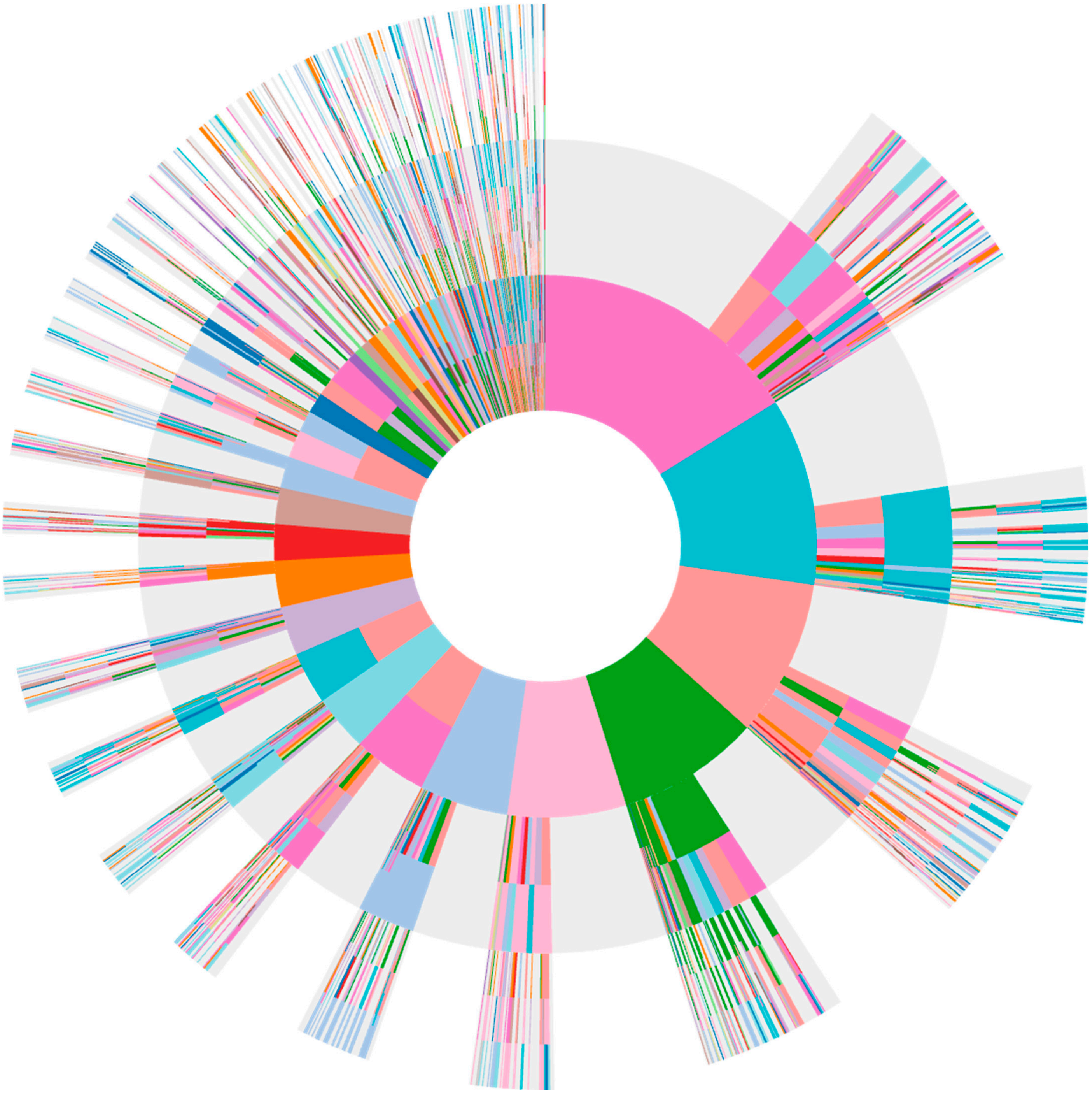

Figure S1a. AUMC

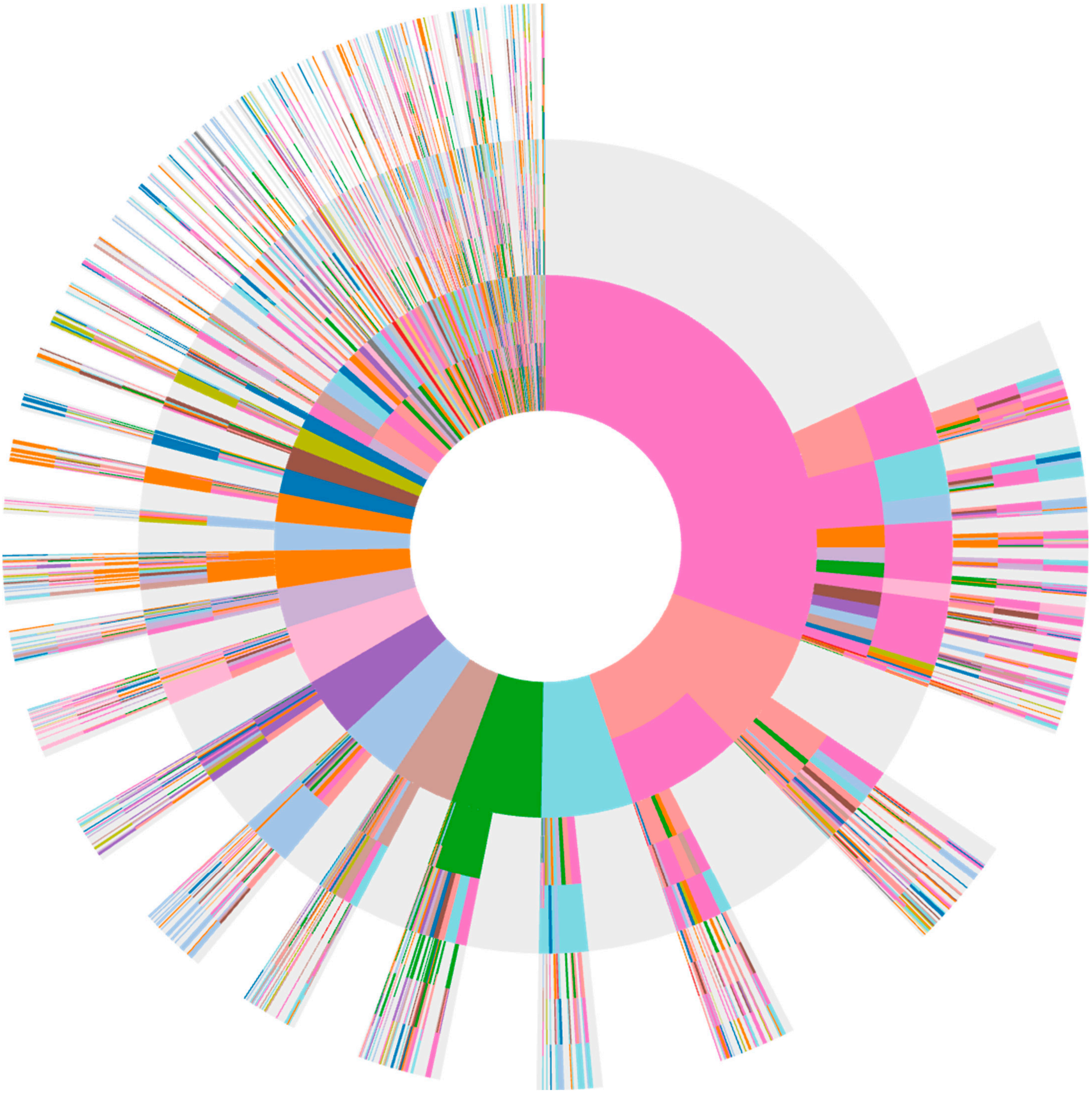

Figure S1b. EUMC

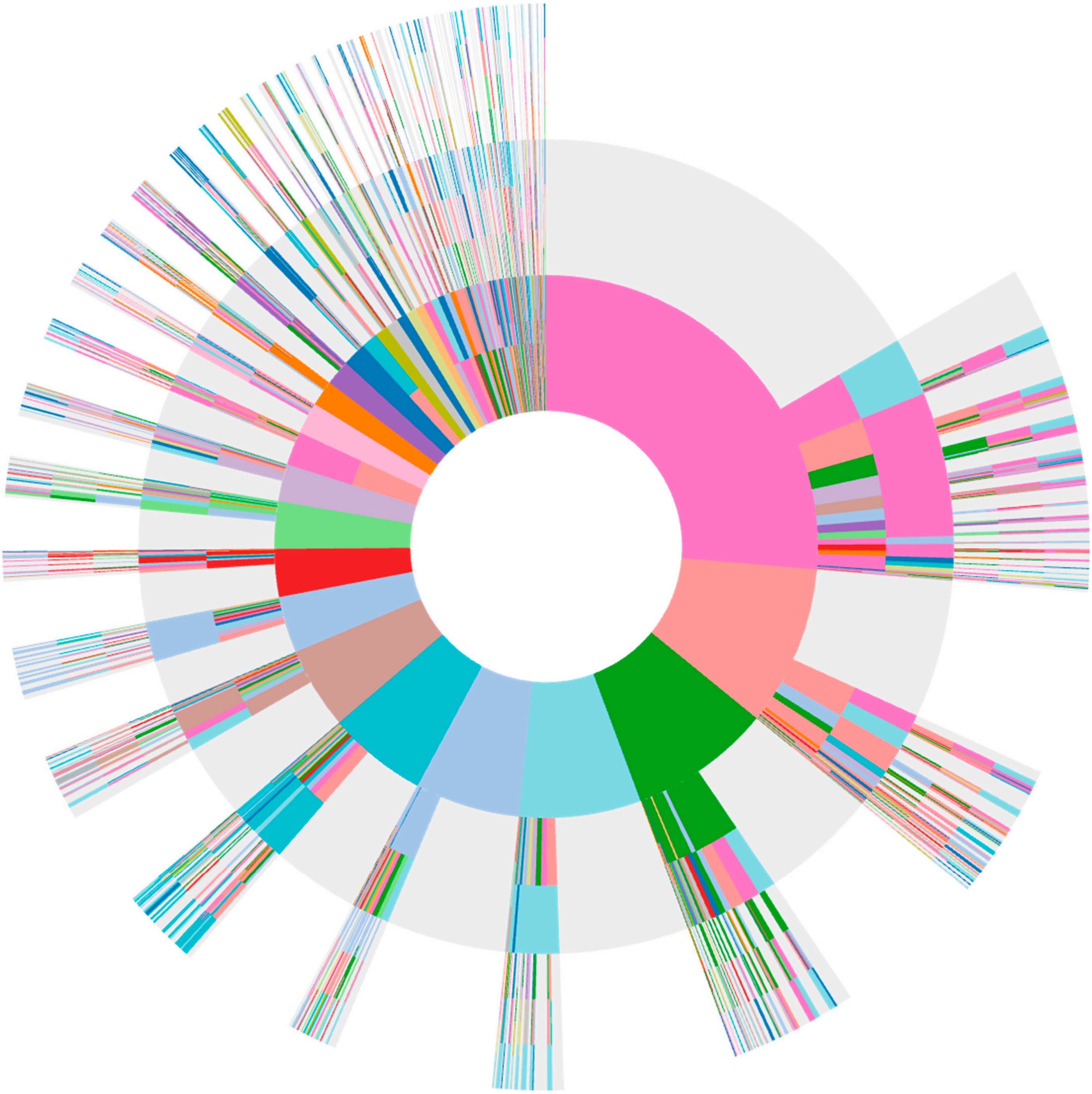

Figure S1c. KDH

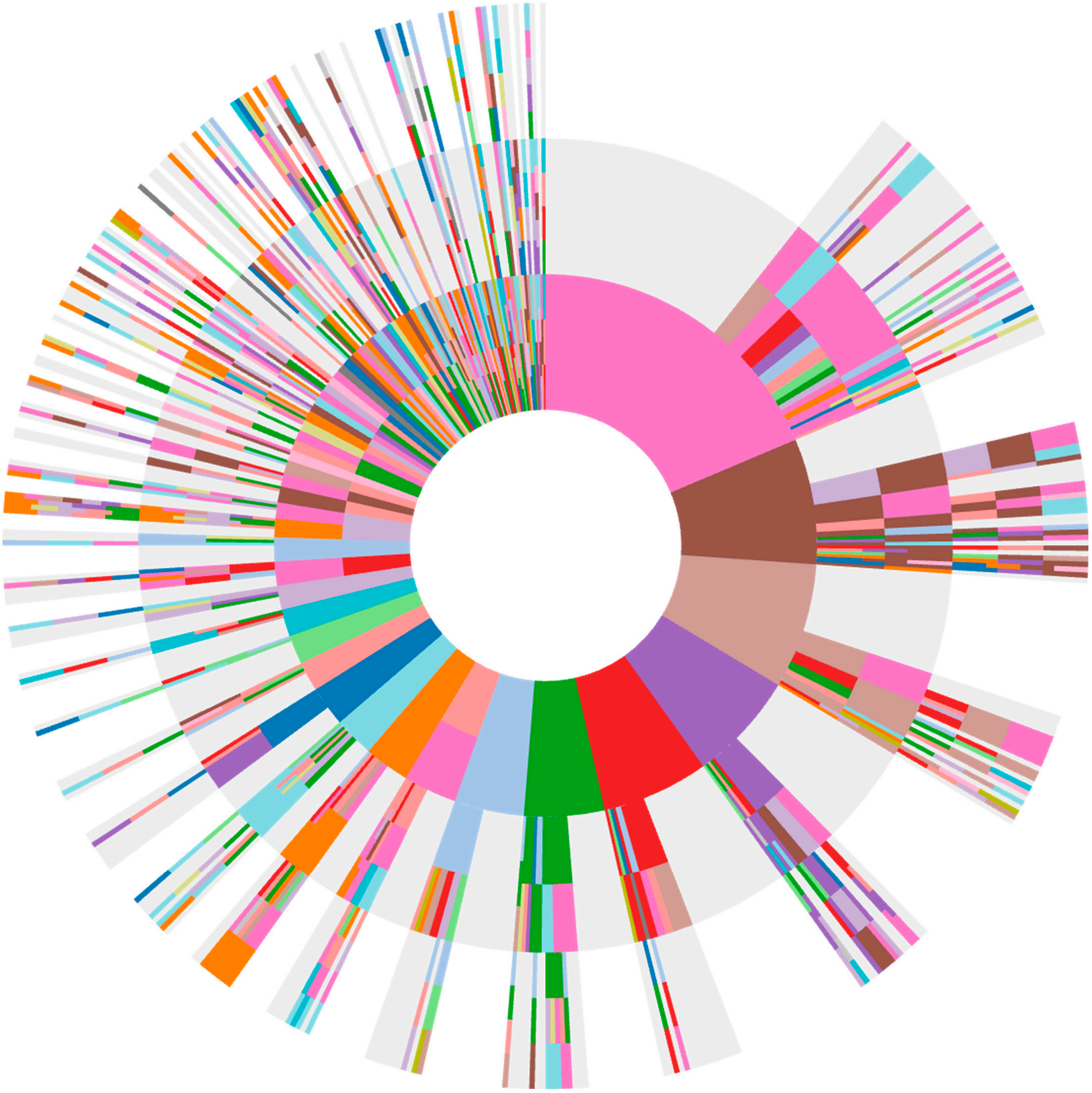

Figure S1d. KHNMC

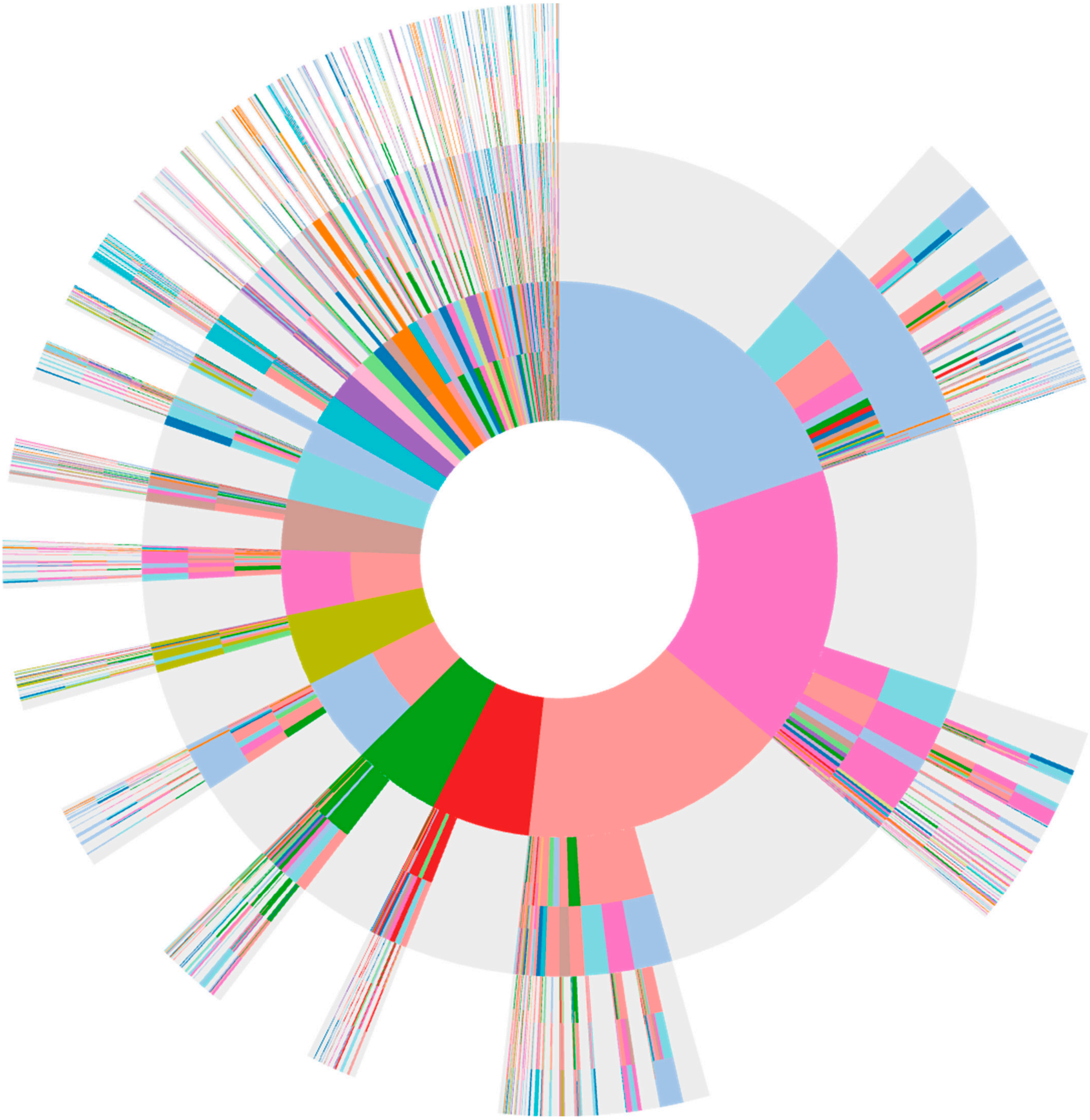

Figure S1e. KWMC

Supplementary Figure S2(a-b). Secular Prescribing Trends by Sex, Age, and Calendar Period (KDH, KWMC)

Sunburst plots showing treatment pathways at the two hospitals with sufficient case volume for period-stratified analysis (KDH, escitalopram-predominant; KWMC, sertraline-predominant), stratified by sex (male, female), age group (<65 vs. ≥65 years), and calendar period (before vs. 2017 and after).

KDH		Male	Female
65 and older	2017 and after	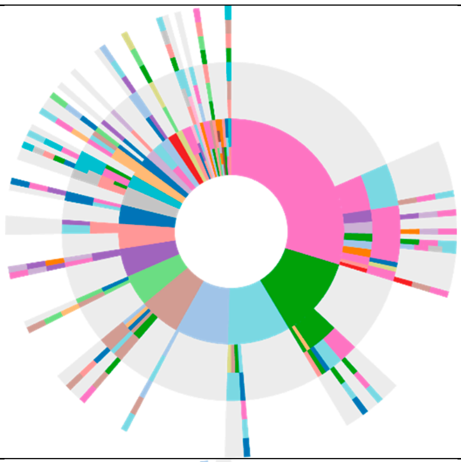	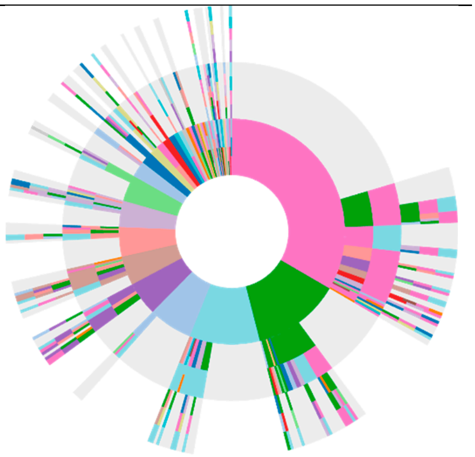
65 and older	Before 2017	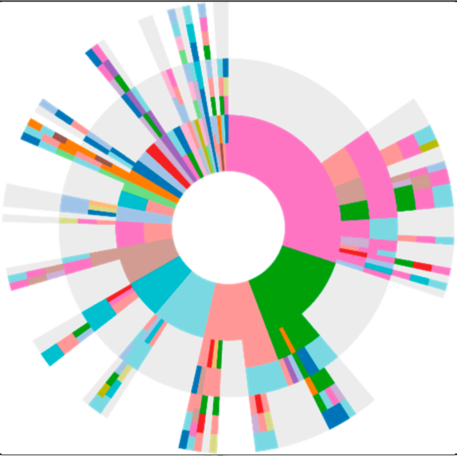	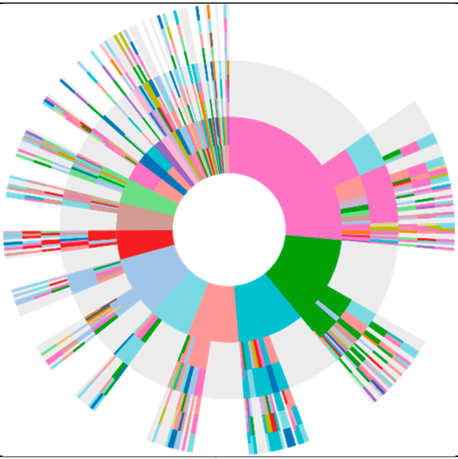
Under 65	2017 and after	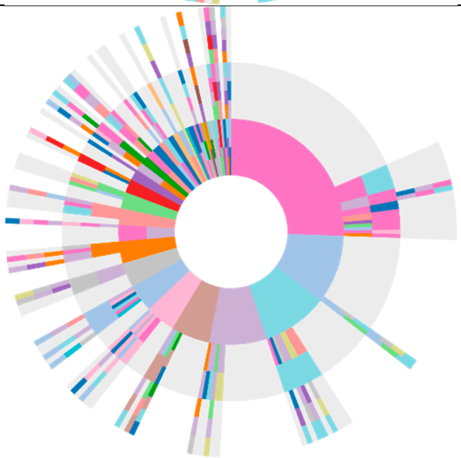	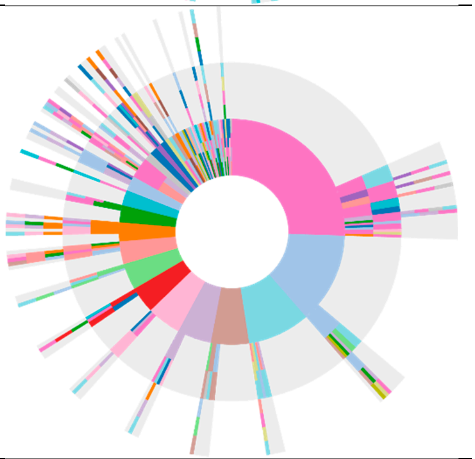
Under 65	Before 2017	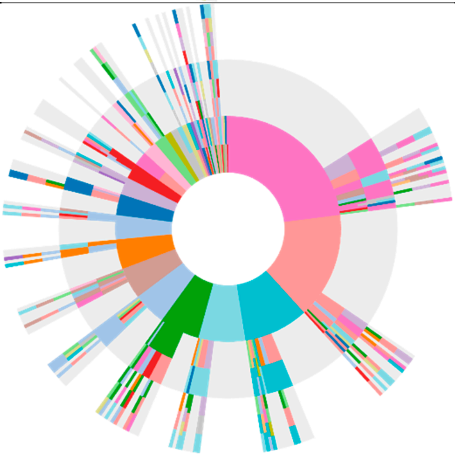	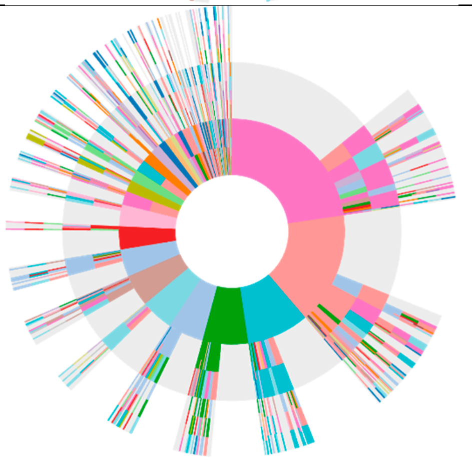

Supplementary Figure S2a. Secular Prescribing Trends by Sex, Age, and Calendar Period (KDH)

KWMC		Male	Female
65 and older	2017 and after	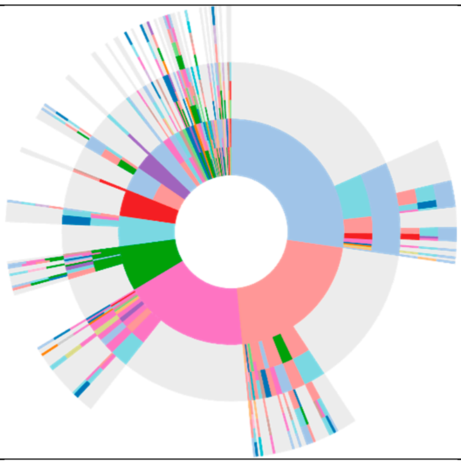	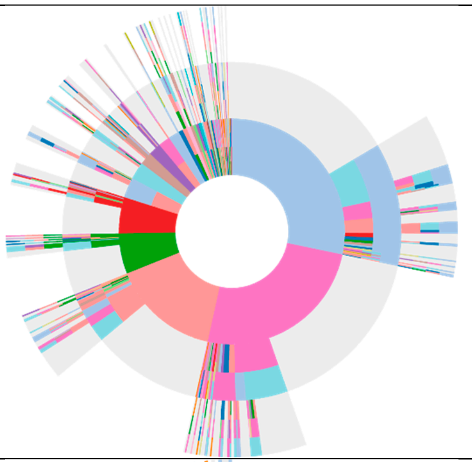
65 and older	Before 2017	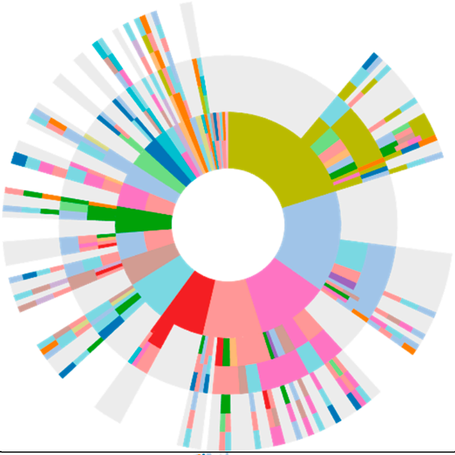	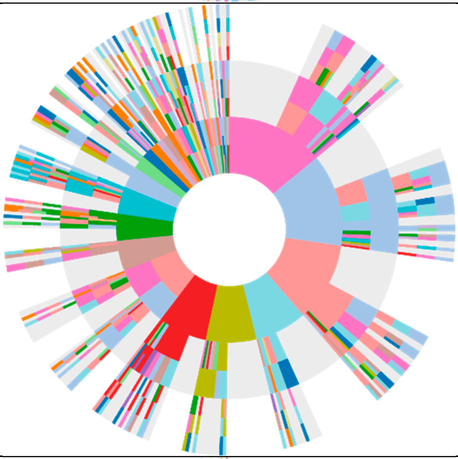
Under 65	2017 and after	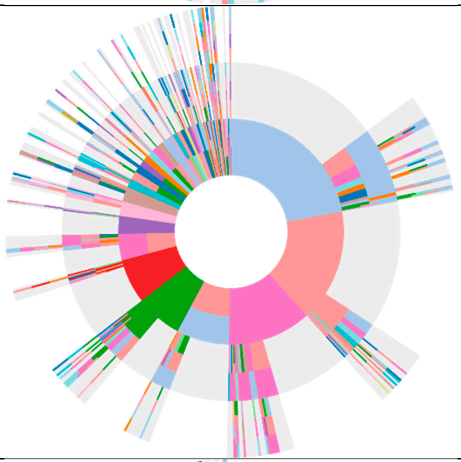	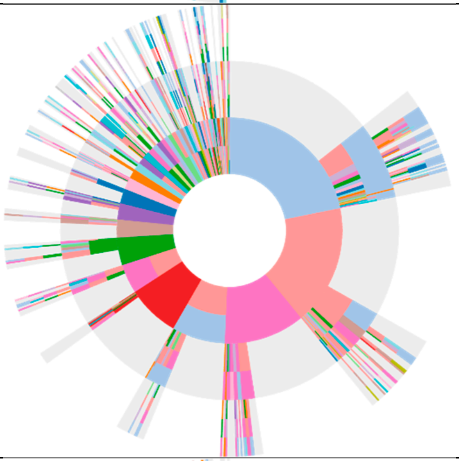
Under 65	Before 2017	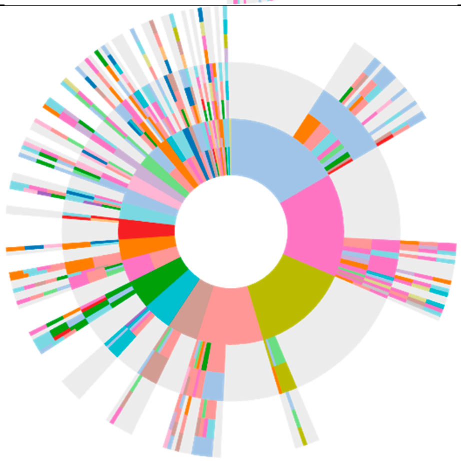	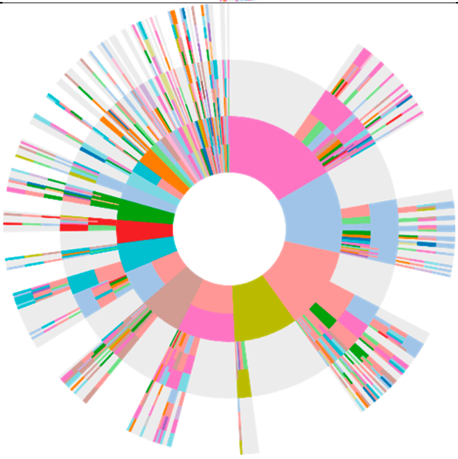

Supplementary Figure S2b. Secular Prescribing Trends by Sex, Age, and Calendar Period (KWMC)

Supplementary Figure S3(a-b). Love Plots of Covariate Balance Before and After Propensity Score Matching

Each panel displays the absolute standardized mean difference for every baseline covariate, before (open circles) and after (filled circles) propensity score matching, for one outcome/drug-comparison pair; covariates are ordered by the magnitude of imbalance before matching. The dashed reference line at 0.1 indicates the conventional threshold below which covariate balance is considered adequate. These plots correspond to the same hospital sets and covariate values summarized numerically in Supplementary Table 1(a-n).

Figure S3a. Escitalopram vs Sertraline

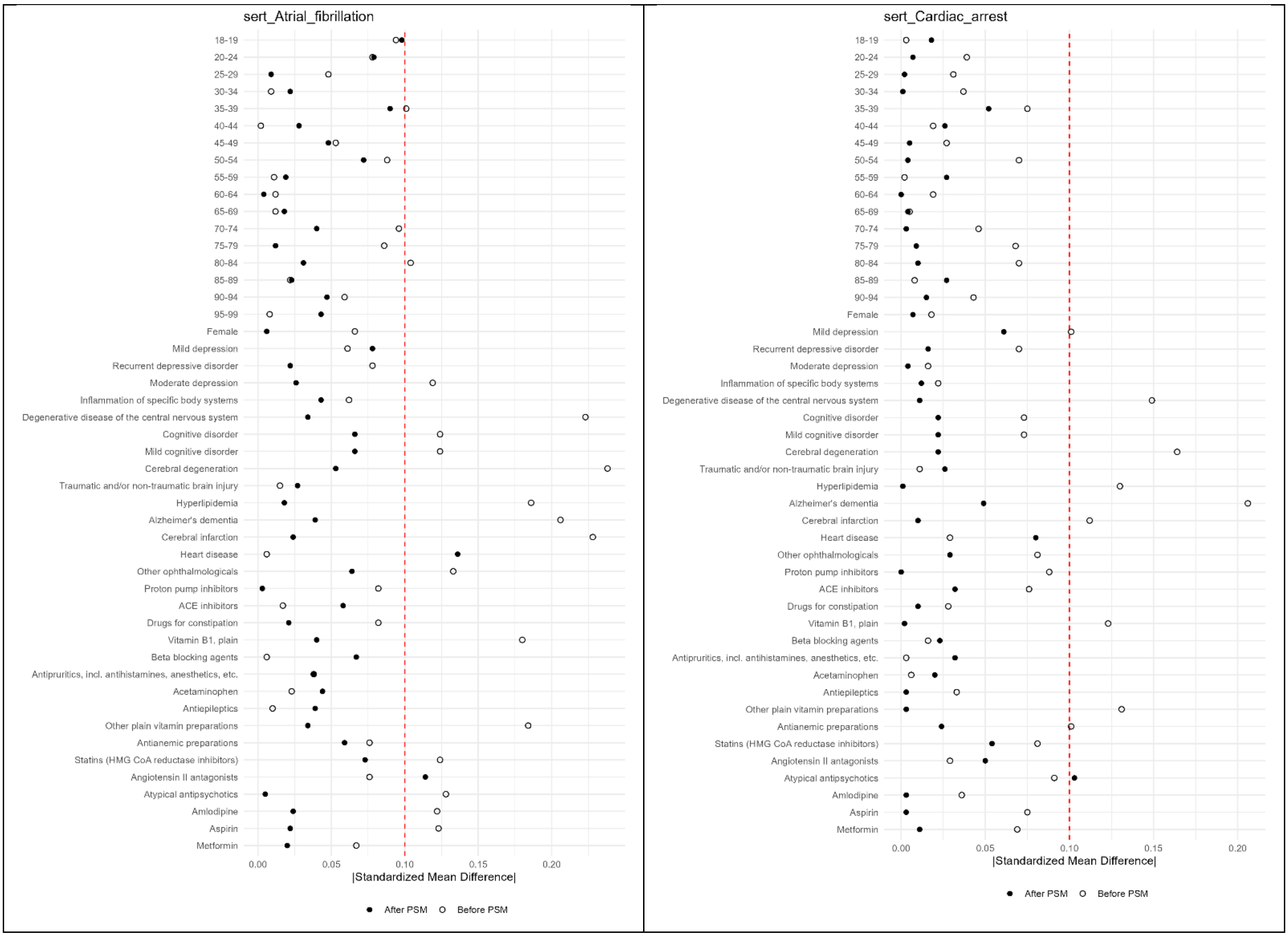

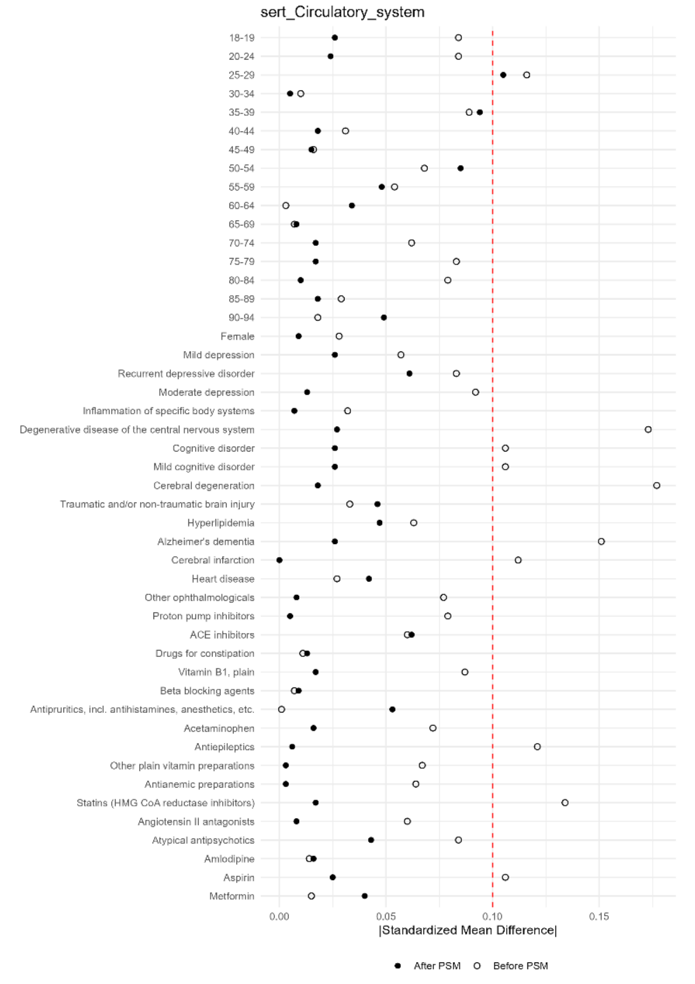
sert_Circulatory_system
18-19
20-24
25-29
30-34
35-39
40-44
45-49
50-54
55-59
60-64
65-69
70-74
75-79
80-84
85-89
90-94
Female
Mild depression
Recurrent depressive disorder
Moderate depression
Inflammation of specific body systems
Degenerative disease of the central nervous system
Cognitive disorder
Mild cognitive disorder
Cerebral degeneration
Traumatic and/or non-traumatic brain injury
Hyperlipidemia
Alzheimer's dementia
Cerebral infarction
Heart disease
Other ophthalmologicals
Proton pump inhibitors
ACE inhibitors
Drugs for constipation
Vitamin B1, plain
Beta blocking agents
Antipruritics, incl. antihistamines, anesthetics, etc.
Acetaminophen
Antiepileptics
Other plain vitamin preparations
Antianemic preparations
Statins (HMG CoA reductase inhibitors)
Angiotensin II antagonists
Atypical antipsychotics
Amlodipine
Aspirin
Metformin
0.00
0.05
0.10
0.15
|Standardized Mean Difference|
After PSM
Before PSM

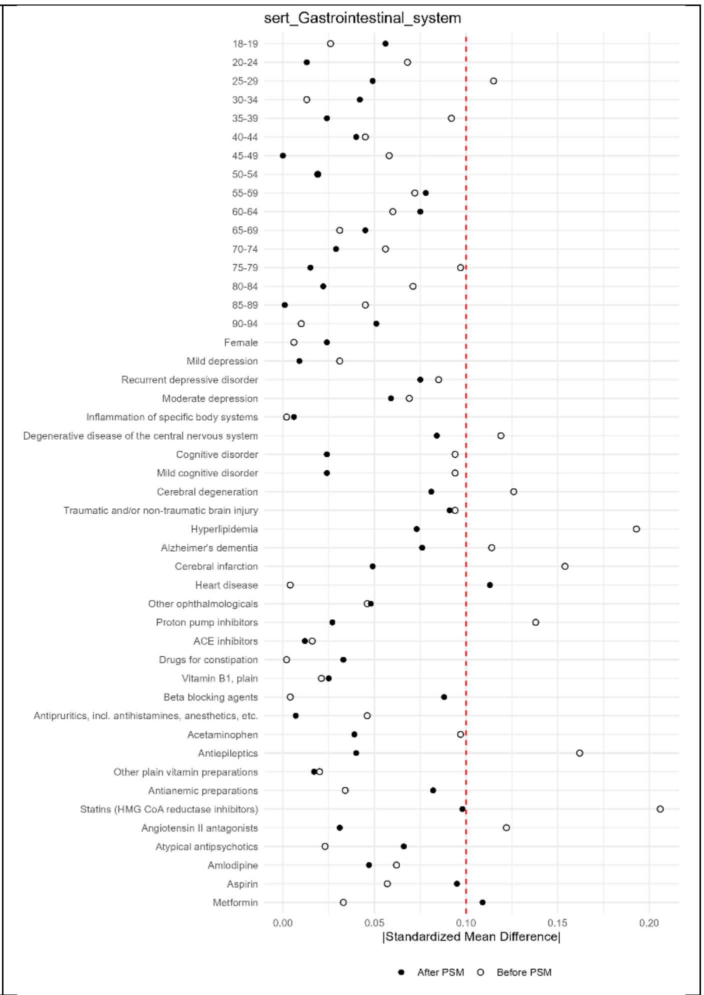
sert_Gastrointestinal_system
18-19
20-24
25-29
30-34
35-39
40-44
45-49
50-54
55-59
60-64
65-69
70-74
75-79
80-84
85-89
90-94
Female
Mild depression
Recurrent depressive disorder
Moderate depression
Inflammation of specific body systems
Degenerative disease of the central nervous system
Cognitive disorder
Mild cognitive disorder
Cerebral degeneration
Traumatic and/or non-traumatic brain injury
Hyperlipidemia
Alzheimer's dementia
Cerebral infarction
Heart disease
Other ophthalmologicals
Proton pump inhibitors
ACE inhibitors
Drugs for constipation
Vitamin B1, plain
Beta blocking agents
Antipruritics, incl. antihistamines, anesthetics, etc.
Acetaminophen
Antiepileptics
Other plain vitamin preparations
Antianemic preparations
Statins (HMG CoA reductase inhibitors)
Angiotensin II antagonists
Atypical antipsychotics
Amlodipine
Aspirin
Metformin
0.00
0.05
0.10
0.15
0.20
|Standardized Mean Difference|
After PSM
Before PSM

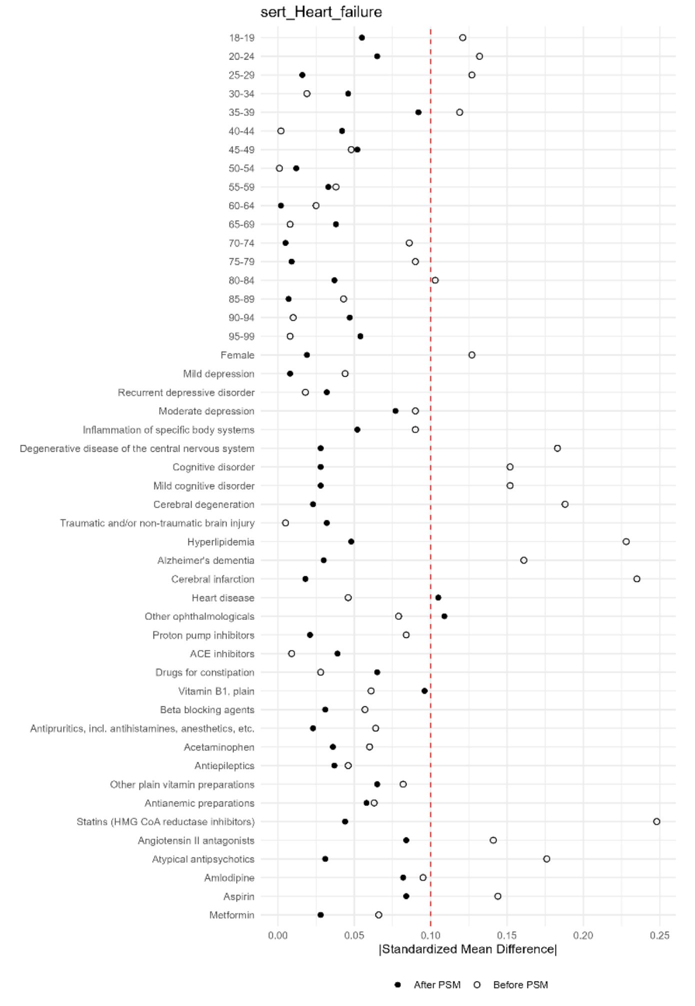
sert_Heart_failure
18-19
20-24
25-29
30-34
35-39
40-44
45-49
50-54
55-59
60-64
65-69
70-74
75-79
80-84
85-89
90-94
95-99
Female
Mild depression
Recurrent depressive disorder
Moderate depression
Inflammation of specific body systems
Degenerative disease of the central nervous system
Cognitive disorder
Mild cognitive disorder
Cerebral degeneration
Traumatic and/or non-traumatic brain injury
Hyperlipidemia
Alzheimer's dementia
Cerebral infarction
Heart disease
Other ophthalmologicals
Proton pump inhibitors
ACE inhibitors
Drugs for constipation
Vitamin B1, plain
Beta blocking agents
Antipruritics, incl. antihistamines, anesthetics, etc.
Acetaminophen
Antiepileptics
Other plain vitamin preparations
Antianemic preparations
Statins (HMG CoA reductase inhibitors)
Angiotensin II antagonists
Atypical antipsychotics
Amlodipine
Aspirin
Metformin
0.00
0.05
0.10
0.15
0.20
0.25
|Standardized Mean Difference|
After PSM
Before PSM

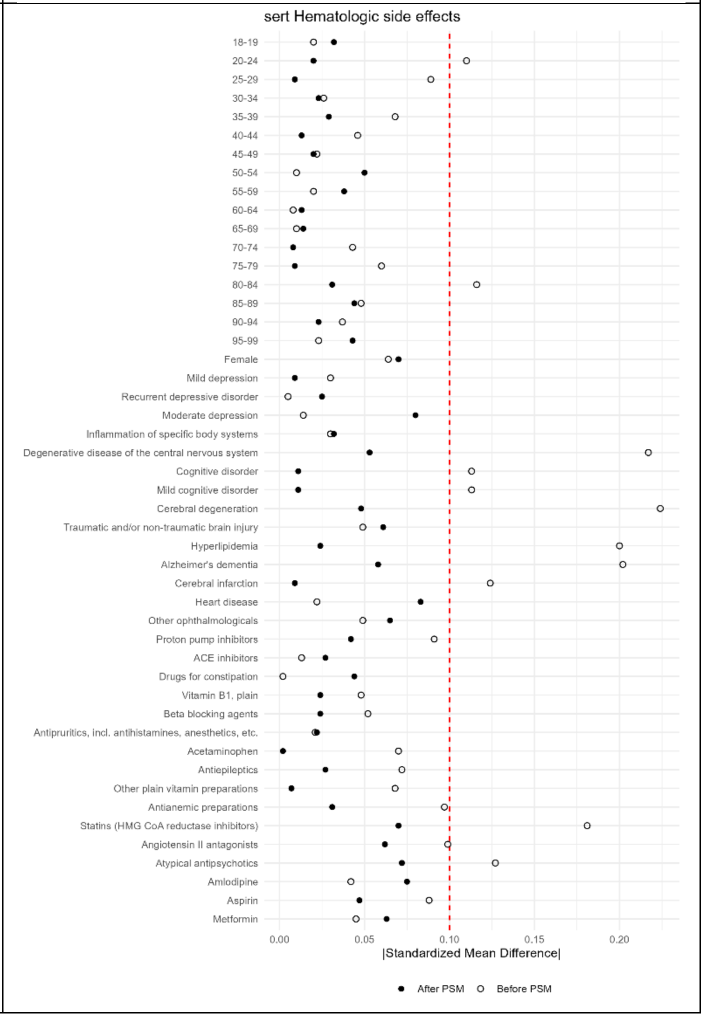
sert Hematologic side effects
18-19
20-24
25-29
30-34
35-39
40-44
45-49
50-54
55-59
60-64
65-69
70-74
75-79
80-84
85-89
90-94
95-99
Female
Mild depression
Recurrent depressive disorder
Moderate depression
Inflammation of specific body systems
Degenerative disease of the central nervous system
Cognitive disorder
Mild cognitive disorder
Cerebral degeneration
Traumatic and/or non-traumatic brain injury
Hyperlipidemia
Alzheimer's dementia
Cerebral infarction
Heart disease
Other ophthalmologicals
Proton pump inhibitors
ACE inhibitors
Drugs for constipation
Vitamin B1, plain
Beta blocking agents
Antipruritics, incl. antihistamines, anesthetics, etc.
Acetaminophen
Antiepileptics
Other plain vitamin preparations
Antianemic preparations
Statins (HMG CoA reductase inhibitors)
Angiotensin II antagonists
Atypical antipsychotics
Amlodipine
Aspirin
Metformin
0.00
0.05
0.10
0.15
0.20
|Standardized Mean Difference|
After PSM
Before PSM

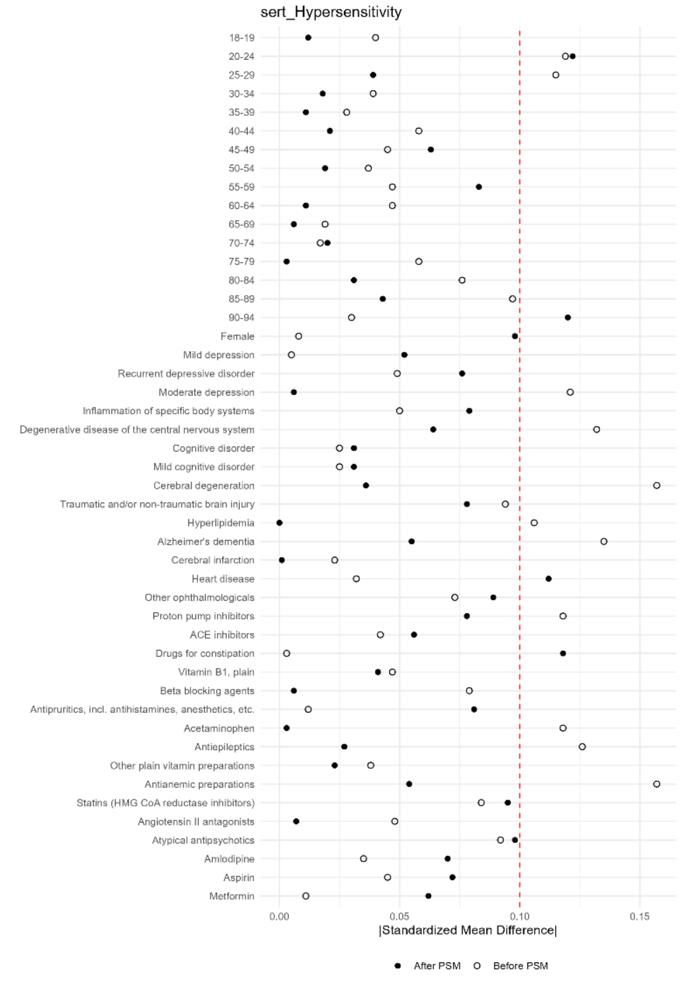
sert_Hypersensitivity
18-19
20-24
25-29
30-34
35-39
40-44
45-49
50-54
55-59
60-64
65-69
70-74
75-79
80-84
85-89
90-94
Female
Mild depression
Recurrent depressive disorder
Moderate depression
Inflammation of specific body systems
Degenerative disease of the central nervous system
Cognitive disorder
Mild cognitive disorder
Cerebral degeneration
Traumatic and/or non-traumatic brain injury
Hyperlipidemia
Alzheimer's dementia
Cerebral infarction
Heart disease
Other ophthalmologicals
Proton pump inhibitors
ACE inhibitors
Drugs for constipation
Vitamin B1, plain
Beta blocking agents
Antipruritics, incl. antihistamines, anesthetics, etc.
Acetaminophen
Antiepileptics
Other plain vitamin preparations
Antianemic preparations
Statins (HMG CoA reductase inhibitors)
Angiotensin II antagonists
Atypical antipsychotics
Amlodipine
Aspirin
Metformin
0.00
0.05
0.10
0.15
|Standardized Mean Difference|
After PSM
Before PSM
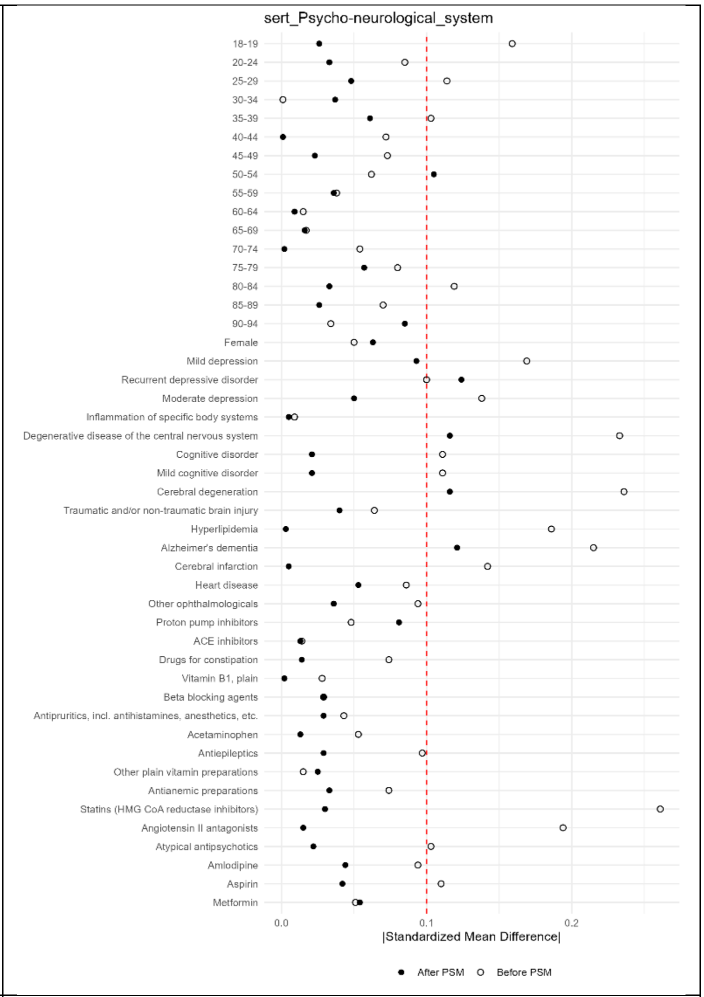
sert_Psycho-neurological_system
18-19
20-24
25-29
30-34
35-39
40-44
45-49
50-54
55-59
60-64
65-69
70-74
75-79
80-84
85-89
90-94
Female
Mild depression
Recurrent depressive disorder
Moderate depression
Inflammation of specific body systems
Degenerative disease of the central nervous system
Cognitive disorder
Mild cognitive disorder
Cerebral degeneration
Traumatic and/or non-traumatic brain injury
Hyperlipidemia
Alzheimer's dementia
Cerebral infarction
Heart disease
Other ophthalmologicals
Proton pump inhibitors
ACE inhibitors
Drugs for constipation
Vitamin B1, plain
Beta blocking agents
Antipruritics, incl. antihistamines, anesthetics, etc.
Acetaminophen
Antiepileptics
Other plain vitamin preparations
Antianemic preparations
Statins (HMG CoA reductase inhibitors)
Angiotensin II antagonists
Atypical antipsychotics
Amlodipine
Aspirin
Metformin
0.0
0.1
0.2
|Standardized Mean Difference|
After PSM
Before PSM
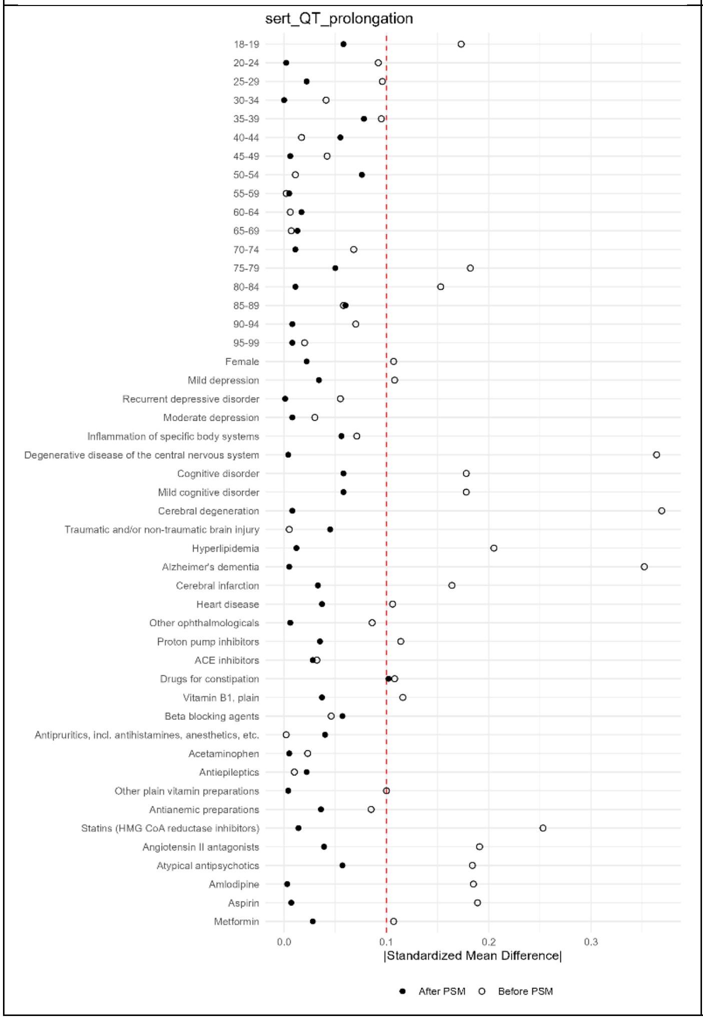
sert_QT_prolongation
18-19
20-24
25-29
30-34
35-39
40-44
45-49
50-54
55-59
60-64
65-69
70-74
75-79
80-84
85-89
90-94
95-99
Female
Mild depression
Recurrent depressive disorder
Moderate depression
Inflammation of specific body systems
Degenerative disease of the central nervous system
Cognitive disorder
Mild cognitive disorder
Cerebral degeneration
Traumatic and/or non-traumatic brain injury
Hyperlipidemia
Alzheimer's dementia
Cerebral infarction
Heart disease
Other ophthalmologicals
Proton pump inhibitors
ACE inhibitors
Drugs for constipation
Vitamin B1, plain
Beta blocking agents
Antipruritics, incl. antihistamines, anesthetics, etc.
Acetaminophen
Antiepileptics
Other plain vitamin preparations
Antianemic preparations
Statins (HMG CoA reductase inhibitors)
Angiotensin II antagonists
Atypical antipsychotics
Amlodipine
Aspirin
Metformin
0.0
0.1
0.2
0.3
|Standardized Mean Difference|
After PSM
Before PSM

Figure S3b. Escitalopram vs Trazodone

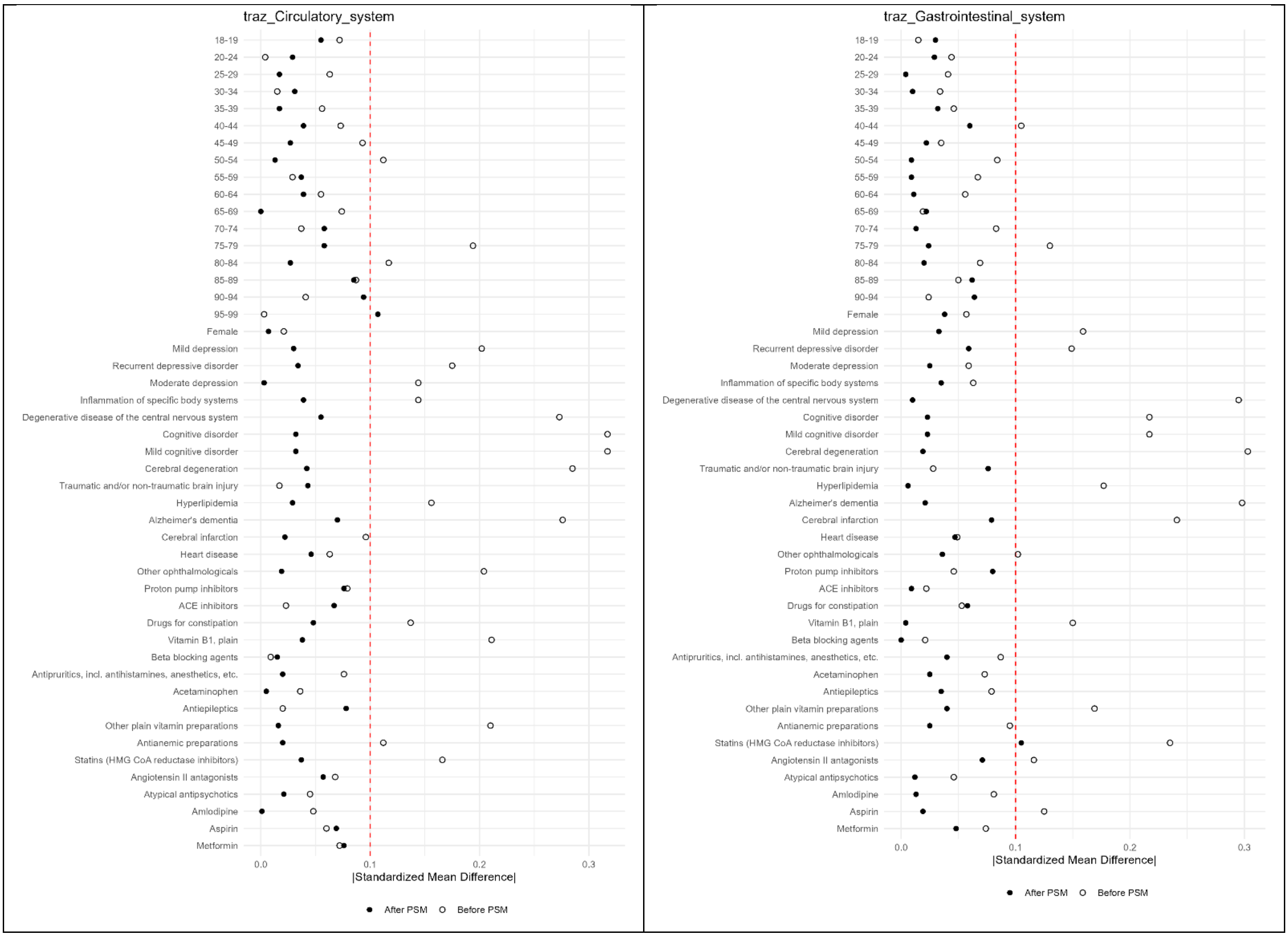

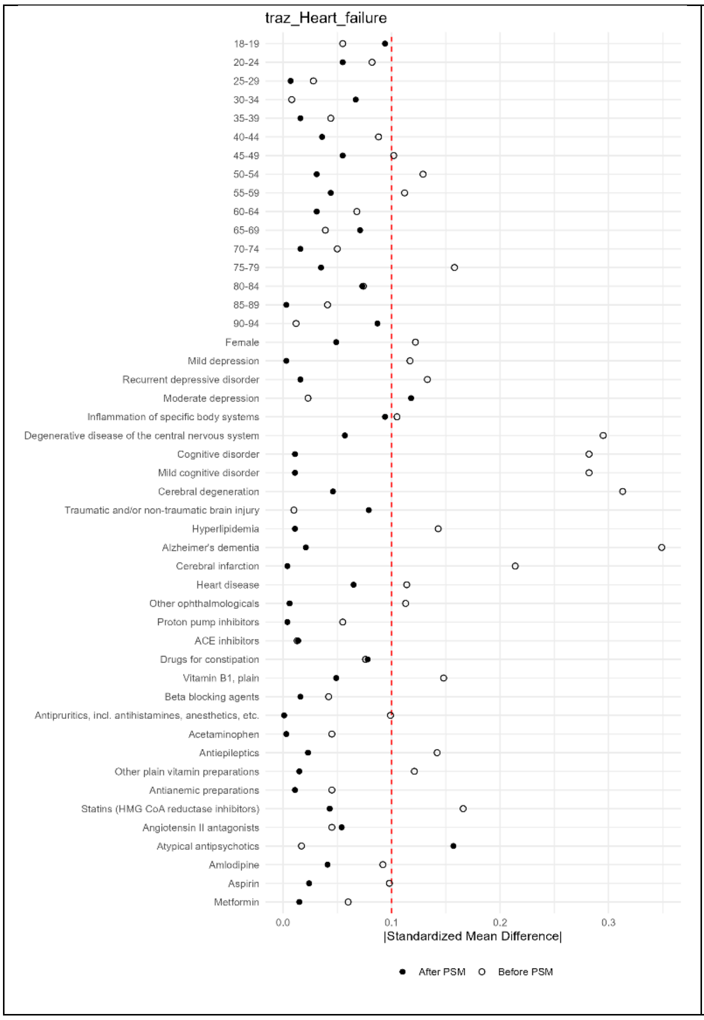
traz_Heart_failure
18-19
20-24
25-29
30-34
35-39
40-44
45-49
50-54
55-59
60-64
65-69
70-74
75-79
80-84
85-89
90-94
Female
Mild depression
Recurrent depressive disorder
Moderate depression
Inflammation of specific body systems
Degenerative disease of the central nervous system
Cognitive disorder
Mild cognitive disorder
Cerebral degeneration
Traumatic and/or non-traumatic brain injury
Hyperlipidemia
Alzheimer's dementia
Cerebral infarction
Heart disease
Other ophthalmologicals
Proton pump inhibitors
ACE inhibitors
Drugs for constipation
Vitamin B1, plain
Beta blocking agents
Antipruritics, incl. antihistamines, anesthetics, etc.
Acetaminophen
Antiepileptics
Other plain vitamin preparations
Antianemic preparations
Statins (HMG CoA reductase inhibitors)
Angiotensin II antagonists
Atypical antipsychotics
Amlodipine
Aspirin
Metformin
0.0
0.1
0.2
0.3
|Standardized Mean Difference|
After PSM
Before PSM

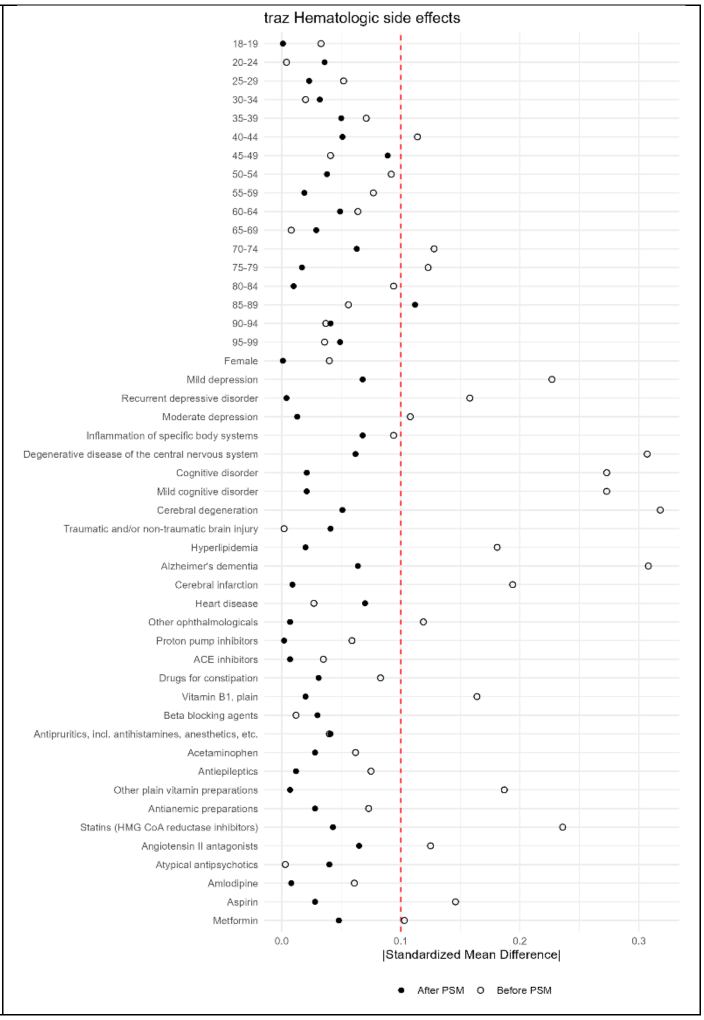
traz Hematologic side effects
18-19
20-24
25-29
30-34
35-39
40-44
45-49
50-54
55-59
60-64
65-69
70-74
75-79
80-84
85-89
90-94
95-99
Female
Mild depression
Recurrent depressive disorder
Moderate depression
Inflammation of specific body systems
Degenerative disease of the central nervous system
Cognitive disorder
Mild cognitive disorder
Cerebral degeneration
Traumatic and/or non-traumatic brain injury
Hyperlipidemia
Alzheimer's dementia
Cerebral infarction
Heart disease
Other ophthalmologicals
Proton pump inhibitors
ACE inhibitors
Drugs for constipation
Vitamin B1, plain
Beta blocking agents
Antipruritics, incl. antihistamines, anesthetics, etc.
Acetaminophen
Antiepileptics
Other plain vitamin preparations
Antianemic preparations
Statins (HMG CoA reductase inhibitors)
Angiotensin II antagonists
Atypical antipsychotics
Amlodipine
Aspirin
Metformin
0.0
0.1
0.2
0.3
|Standardized Mean Difference|
After PSM
Before PSM

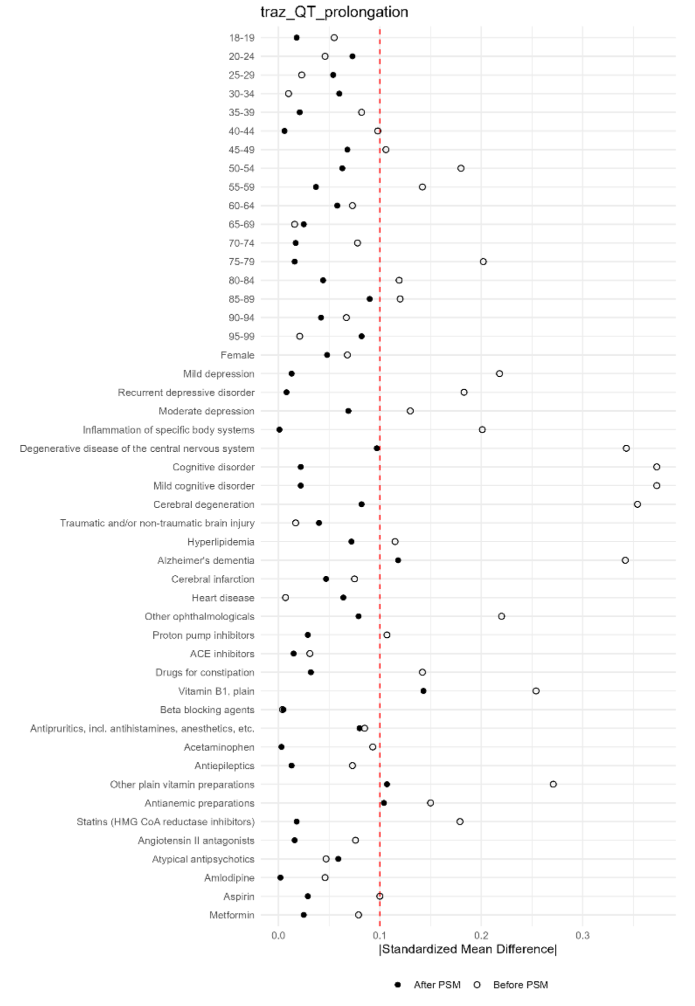
traz_QT_prolongation
18-19
20-24
25-29
30-34
35-39
40-44
45-49
50-54
55-59
60-64
65-69
70-74
75-79
80-84
85-89
90-94
95-99
Female
Mild depression
Recurrent depressive disorder
Moderate depression
Inflammation of specific body systems
Degenerative disease of the central nervous system
Cognitive disorder
Mild cognitive disorder
Cerebral degeneration
Traumatic and/or non-traumatic brain injury
Hyperlipidemia
Alzheimer's dementia
Cerebral infarction
Heart disease
Other ophthalmologicals
Proton pump inhibitors
ACE inhibitors
Drugs for constipation
Vitamin B1, plain
Beta blocking agents
Antipruritics, incl. antihistamines, anesthetics, etc.
Acetaminophen
Antiepileptics
Other plain vitamin preparations
Antianemic preparations
Statins (HMG CoA reductase inhibitors)
Angiotensin II antagonists
Atypical antipsychotics
Amlodipine
Aspirin
Metformin
0.0
0.1
0.2
0.3
|Standardized Mean Difference|
After PSM
Before PSM

Supplementary Figure S4(a-b). Preference Score Distributions Before Propensity Score Matching

Supplementary Figure S4a. Escitalopram vs Sertraline

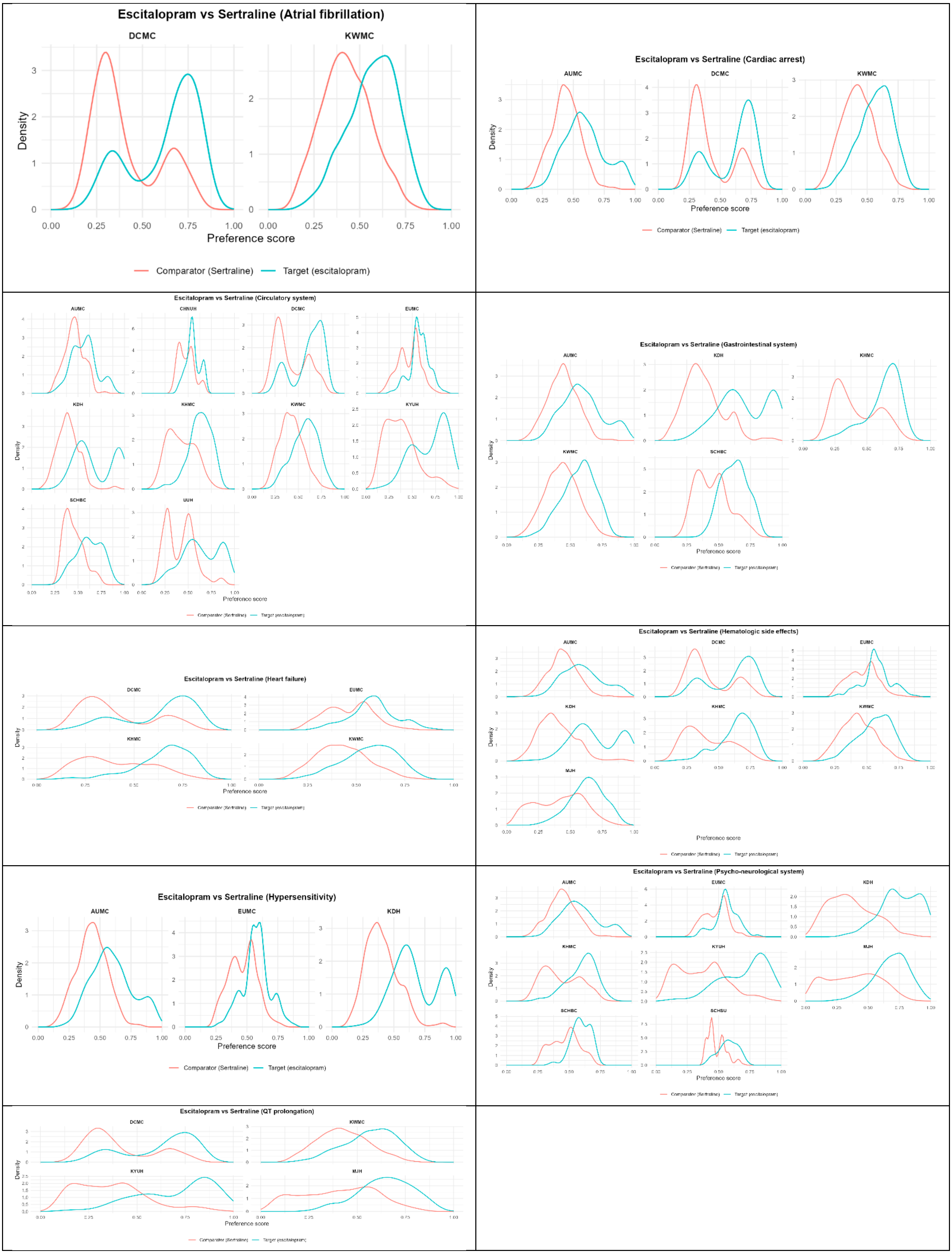

Supplementary Figure S4b. Escitalopram vs Trazodone

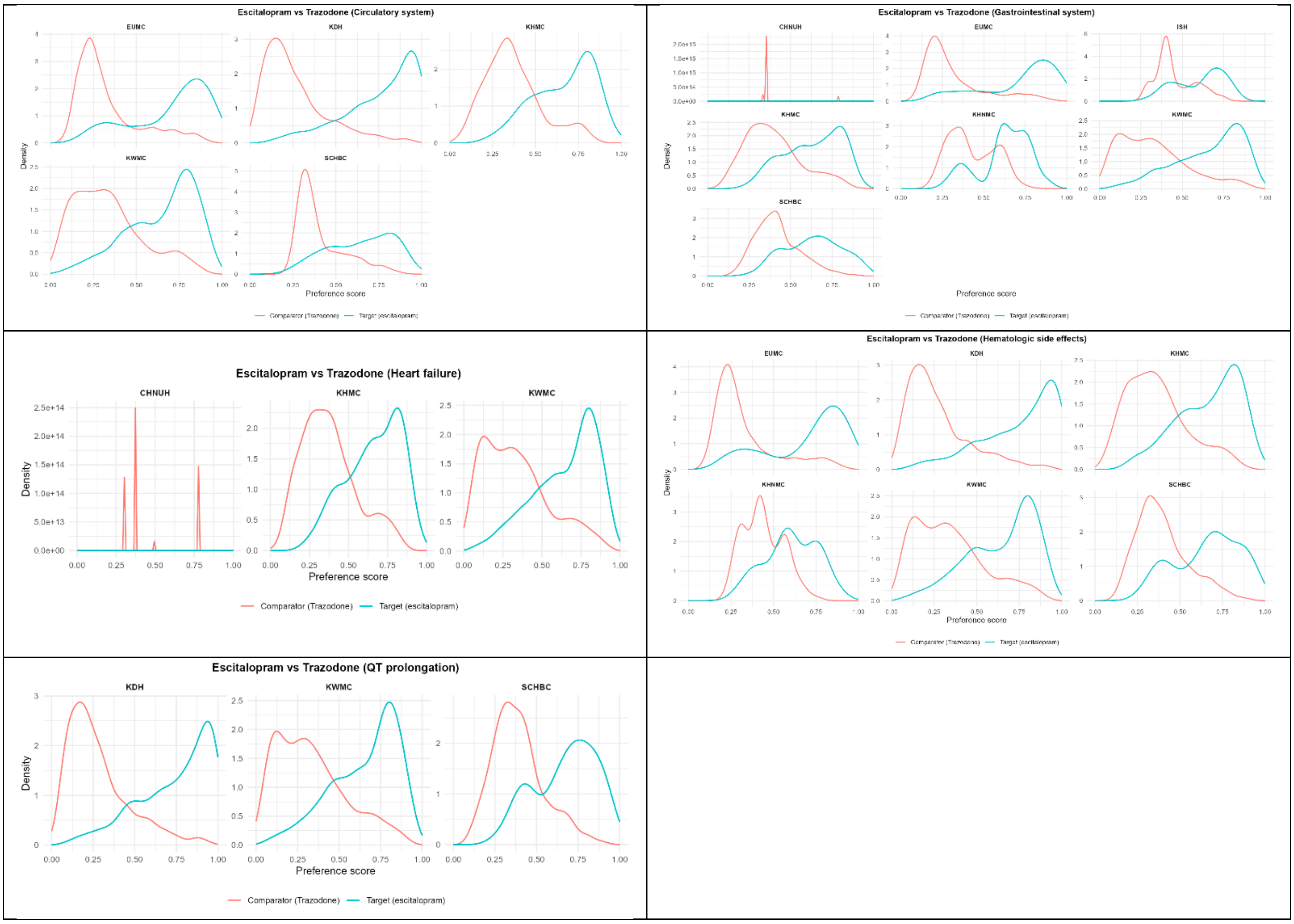

Supplementary Figure S5(a-i). Flow Diagrams (Escitalopram vs. Sertraline)

Flow diagrams showing patient attrition from the original drug-specific cohorts through propensity-score matching for each safety outcome in the escitalopram-versus-sertraline comparison.

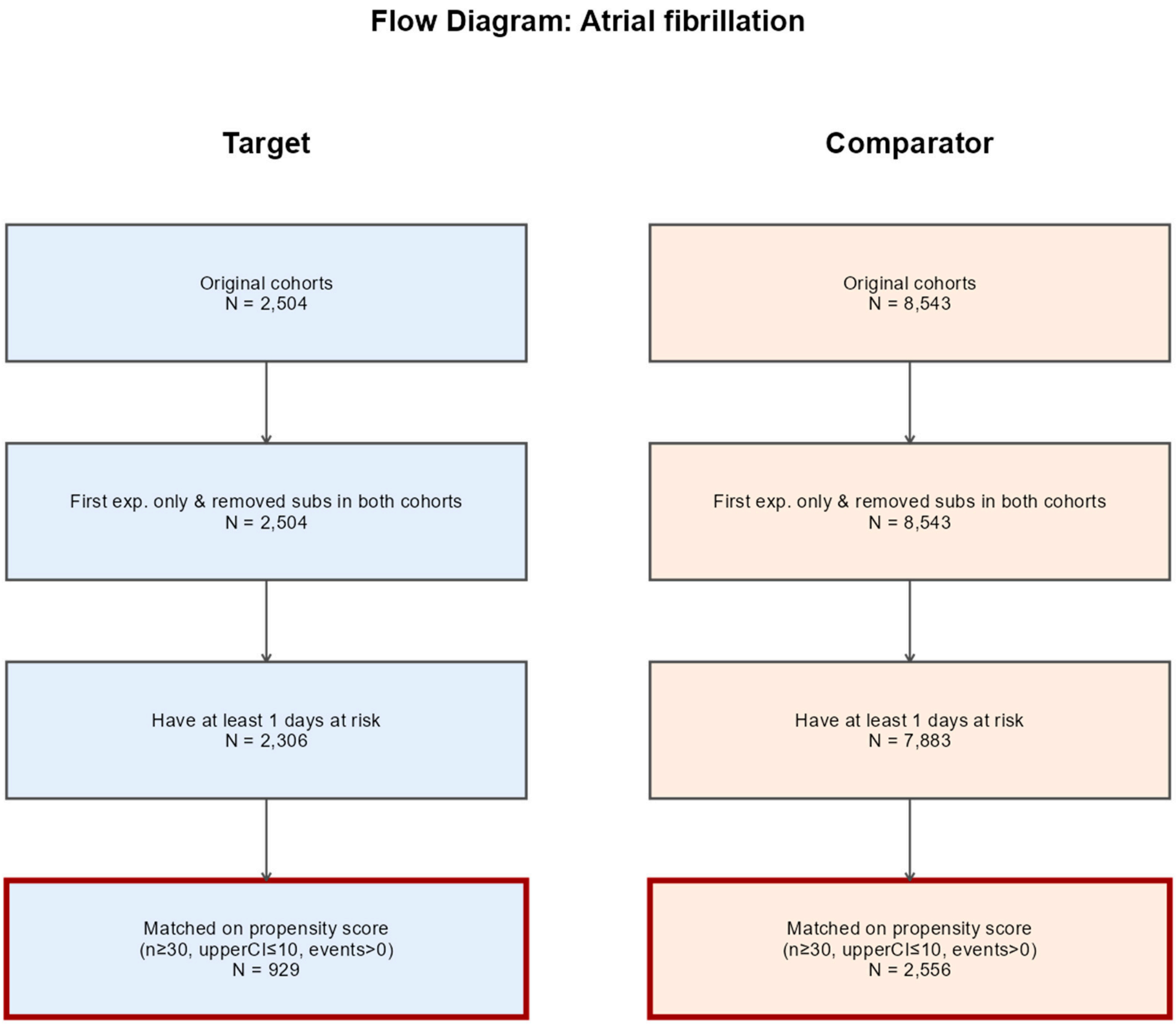

Supplementary Figure S5a. Flow Diagrams (Escitalopram vs. Sertraline), Atrial fibrillation

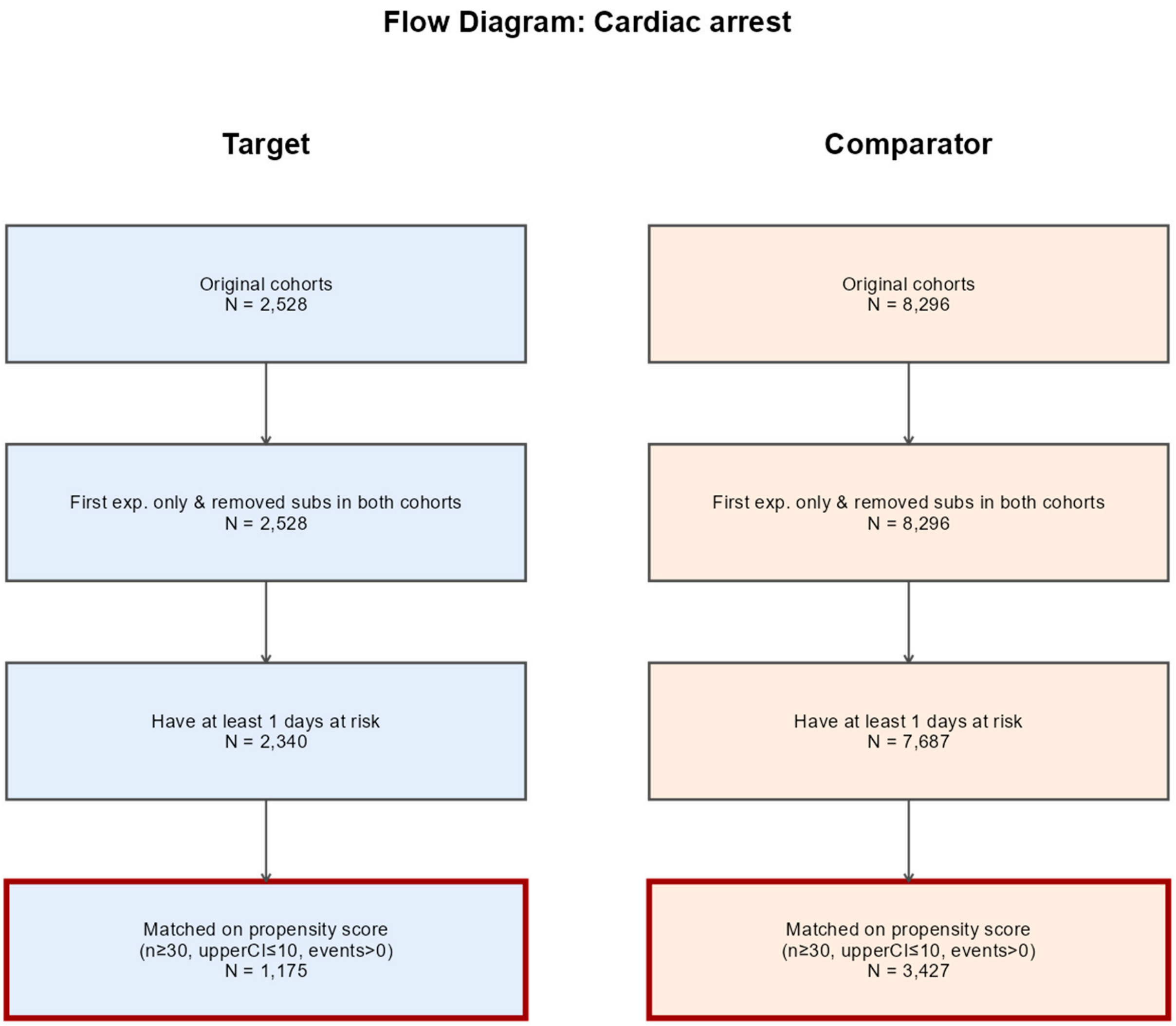

Supplementary Figure S5b. Flow Diagrams (Escitalopram vs. Sertraline), Cardiac arrest

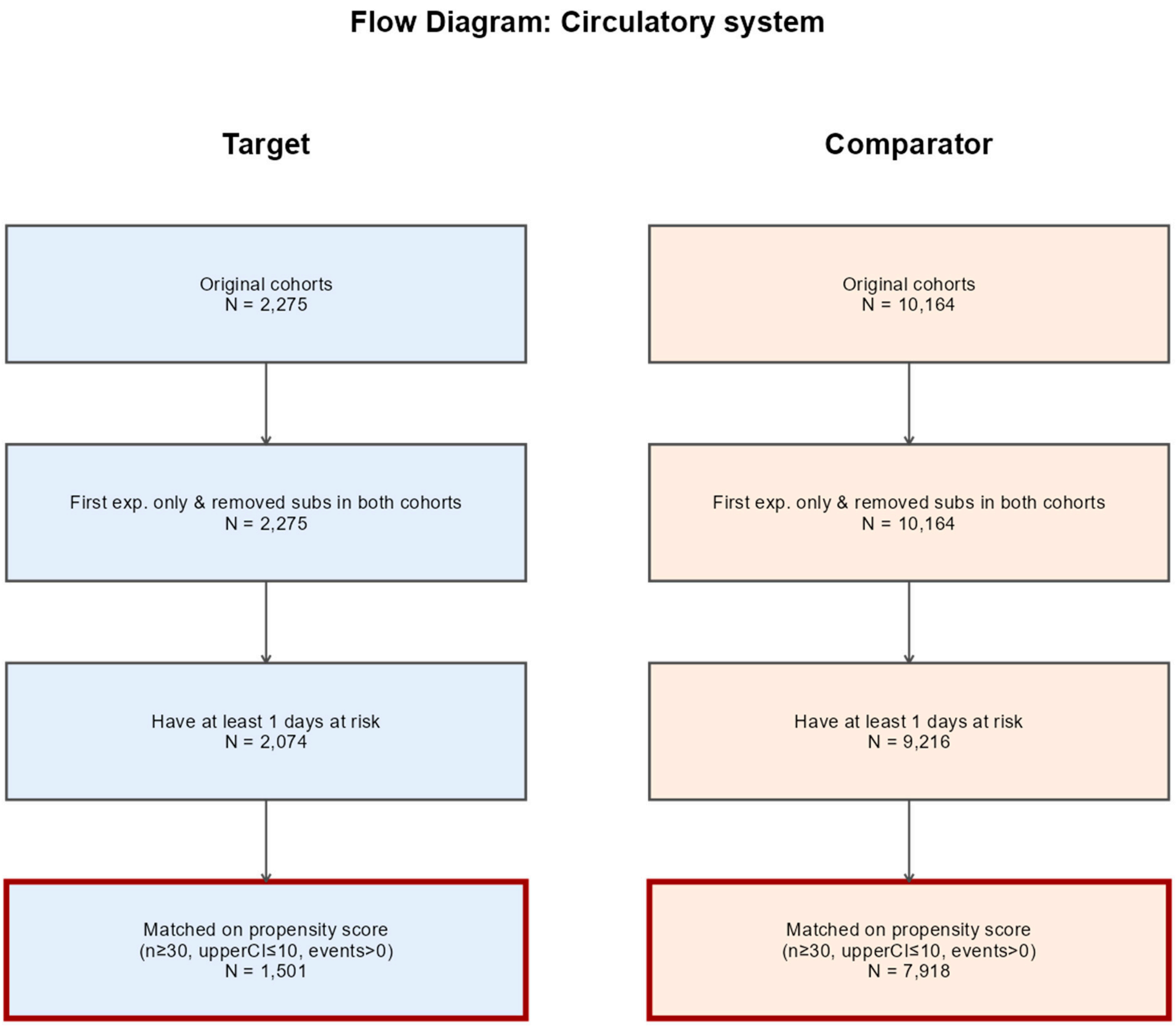

Supplementary Figure S5c. Flow Diagrams (Escitalopram vs. Sertraline), Circulatory system

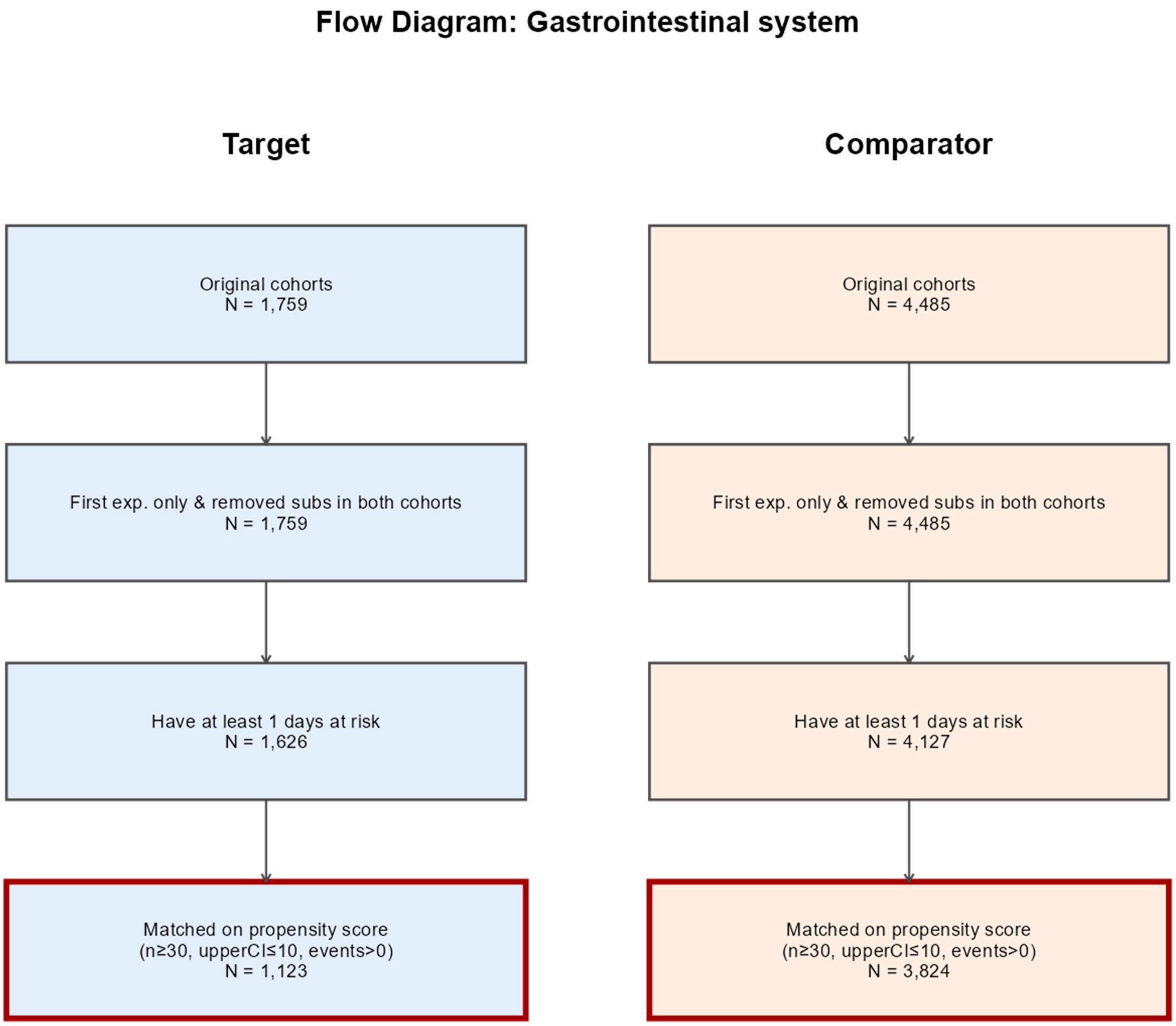

Supplementary Figure S5d. Flow Diagrams (Escitalopram vs. Sertraline), Gastrointestinal system

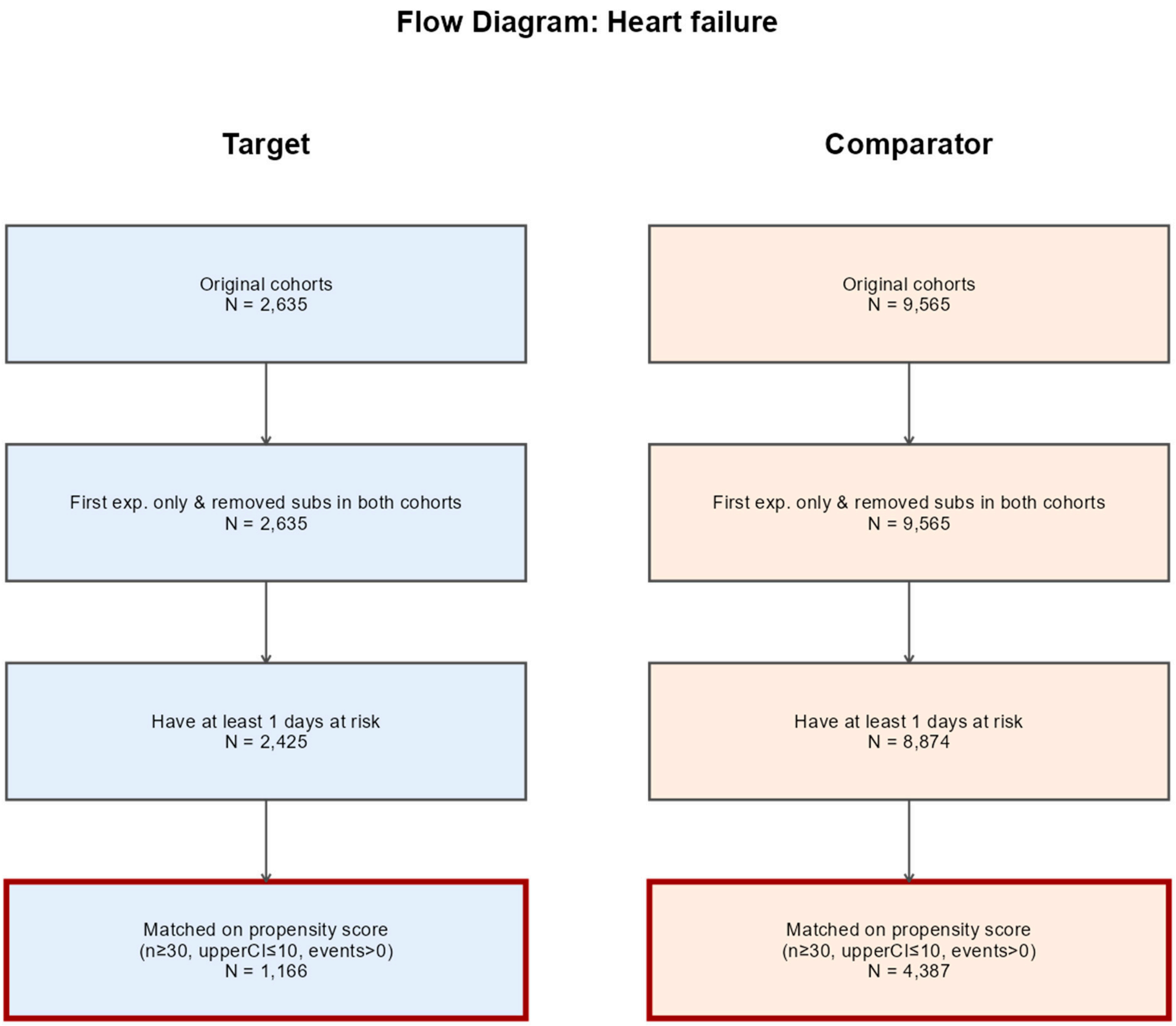

Supplementary Figure S5e. Flow Diagrams (Escitalopram vs. Sertraline), Heart failure

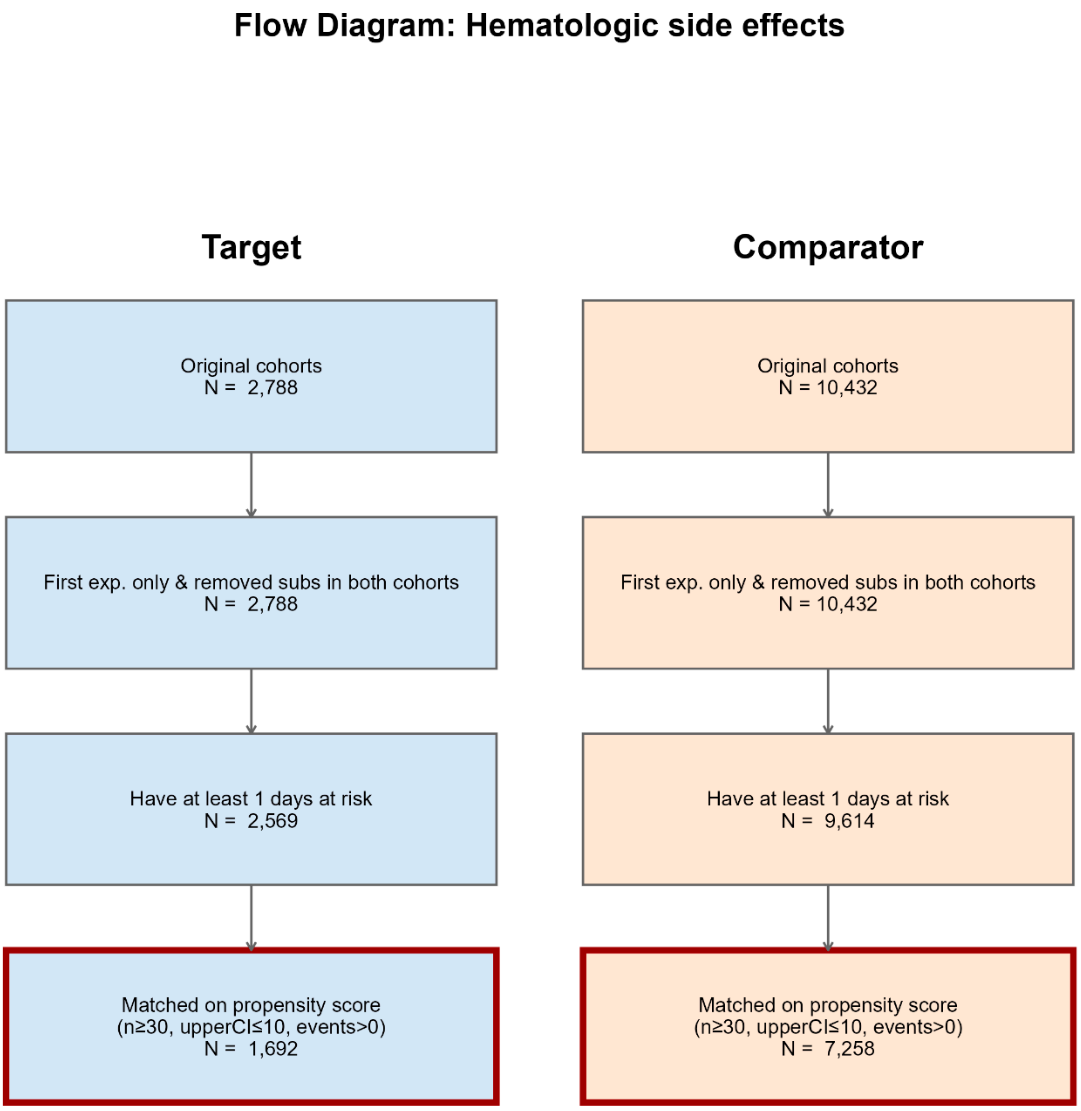

Supplementary Figure S5f. Flow Diagrams (Escitalopram vs. Sertraline), Hematologic side effects

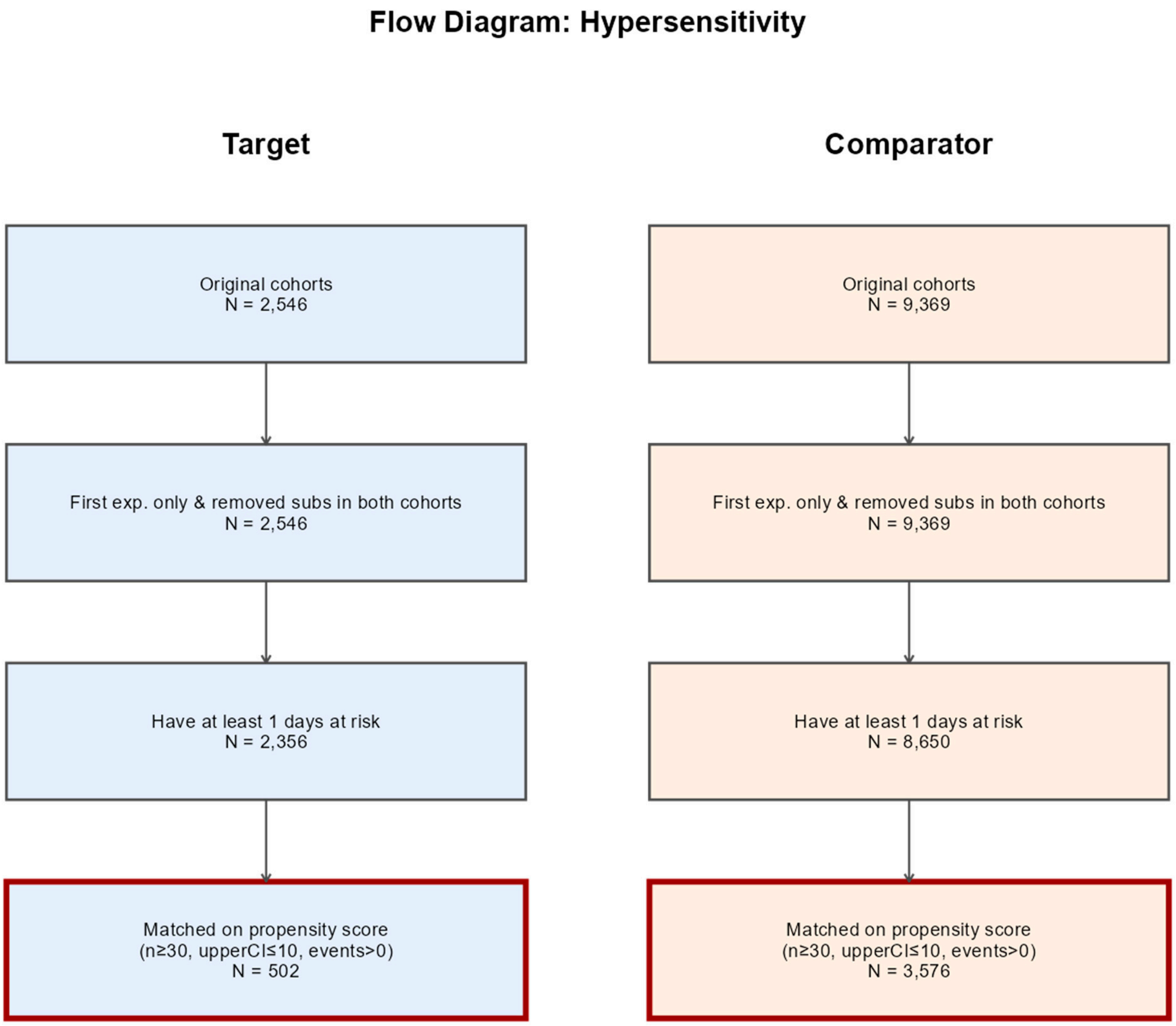

Supplementary Figure S5g. Flow Diagrams (Escitalopram vs. Sertraline), Hypersensitivity

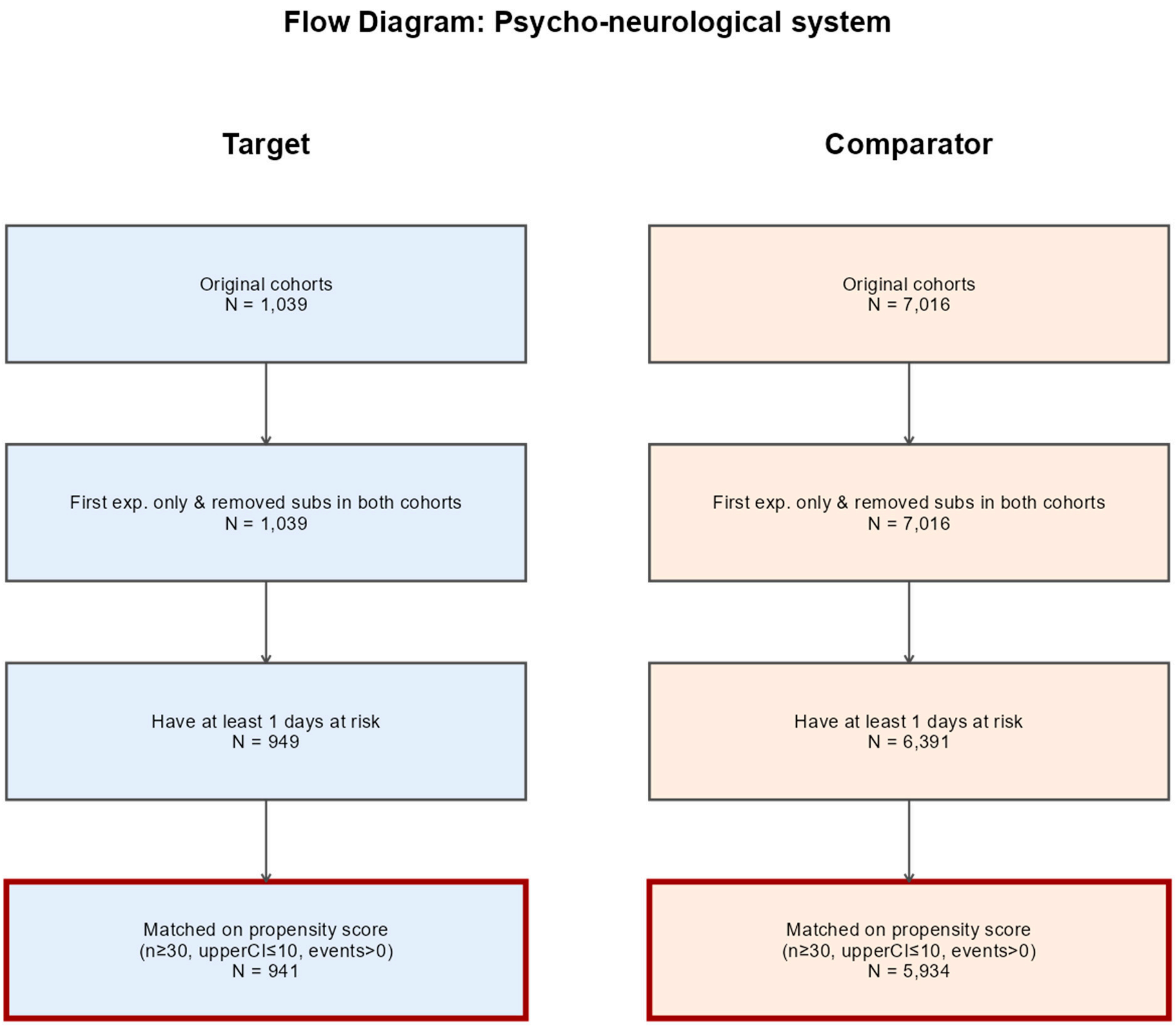

Supplementary Figure S5h. Flow Diagrams (Escitalopram vs. Sertraline), Psycho-neurological system

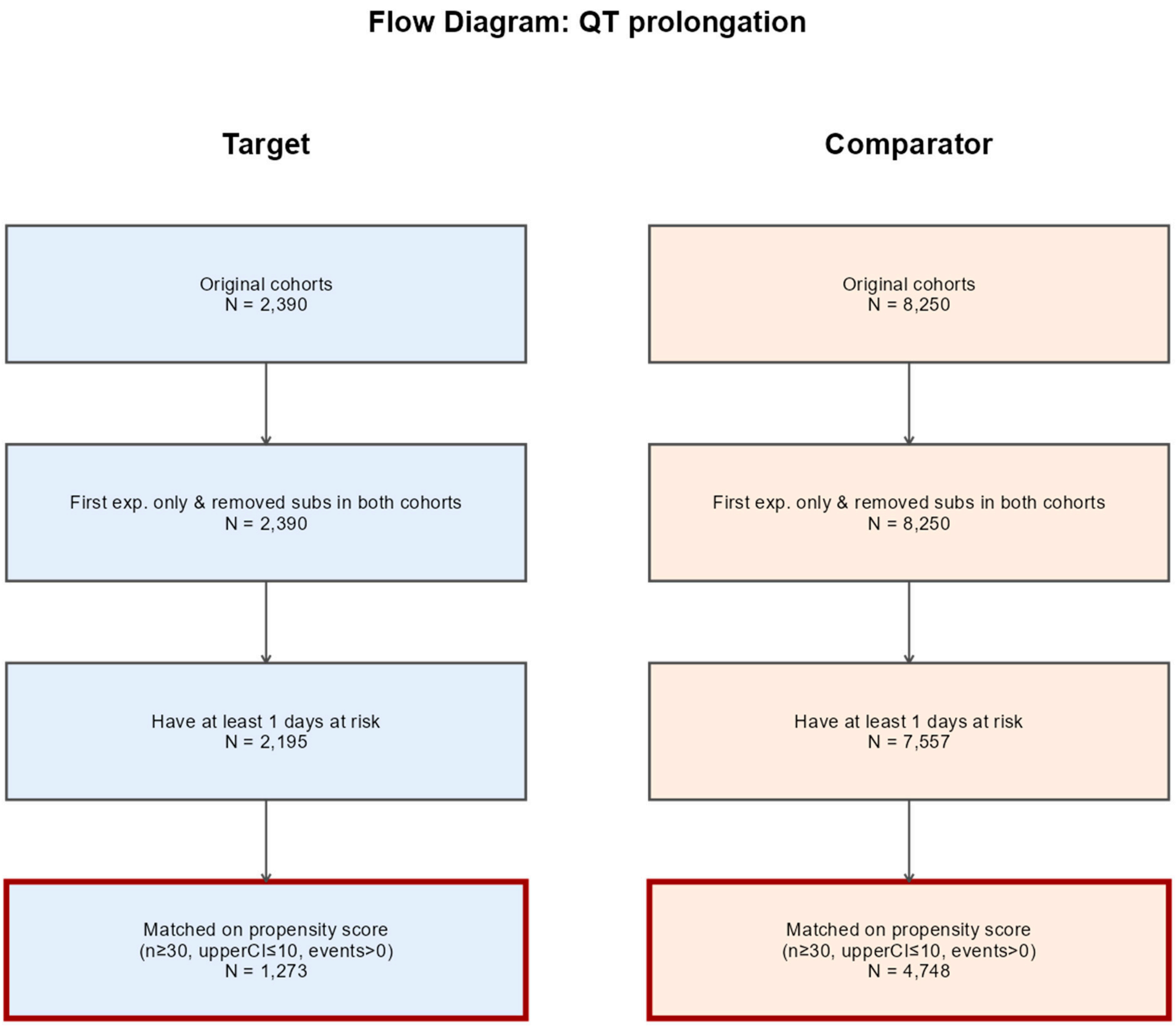

Supplementary Figure S5i. Flow Diagrams (Escitalopram vs. Sertraline), QT prolongation

Supplementary Figure S6(a-e). Flow Diagrams (Escitalopram vs. Trazodone)

Flow diagrams showing patient attrition from the original drug-specific cohorts through propensity-score matching for each safety outcome in the escitalopram-versus-trazodone comparison.

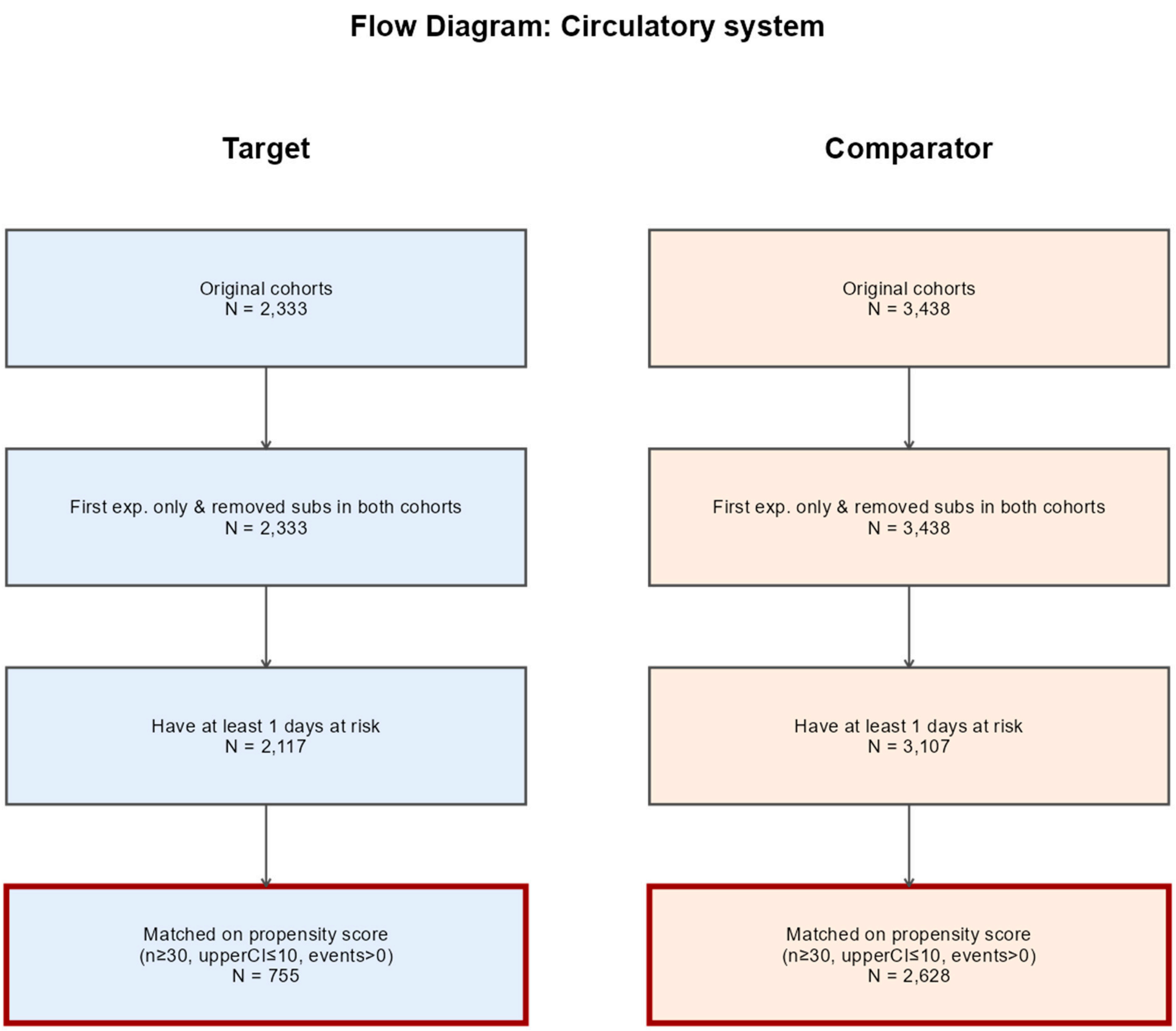

Supplementary Figure S6a. Flow Diagrams (Escitalopram vs. Trazodone), Circulatory system

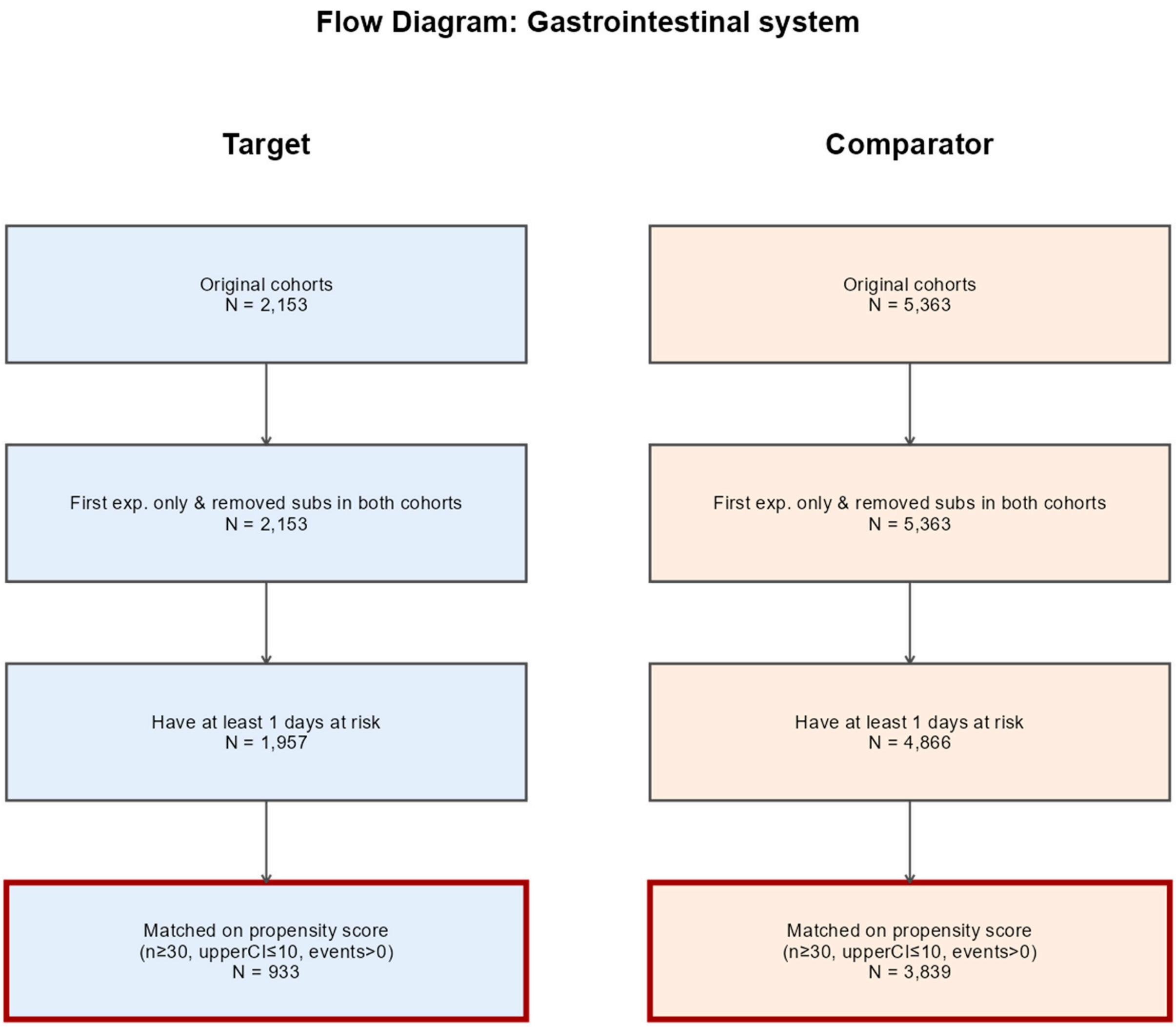

Supplementary Figure S6b. Flow Diagrams (Escitalopram vs. Trazodone), Gastrointestinal system

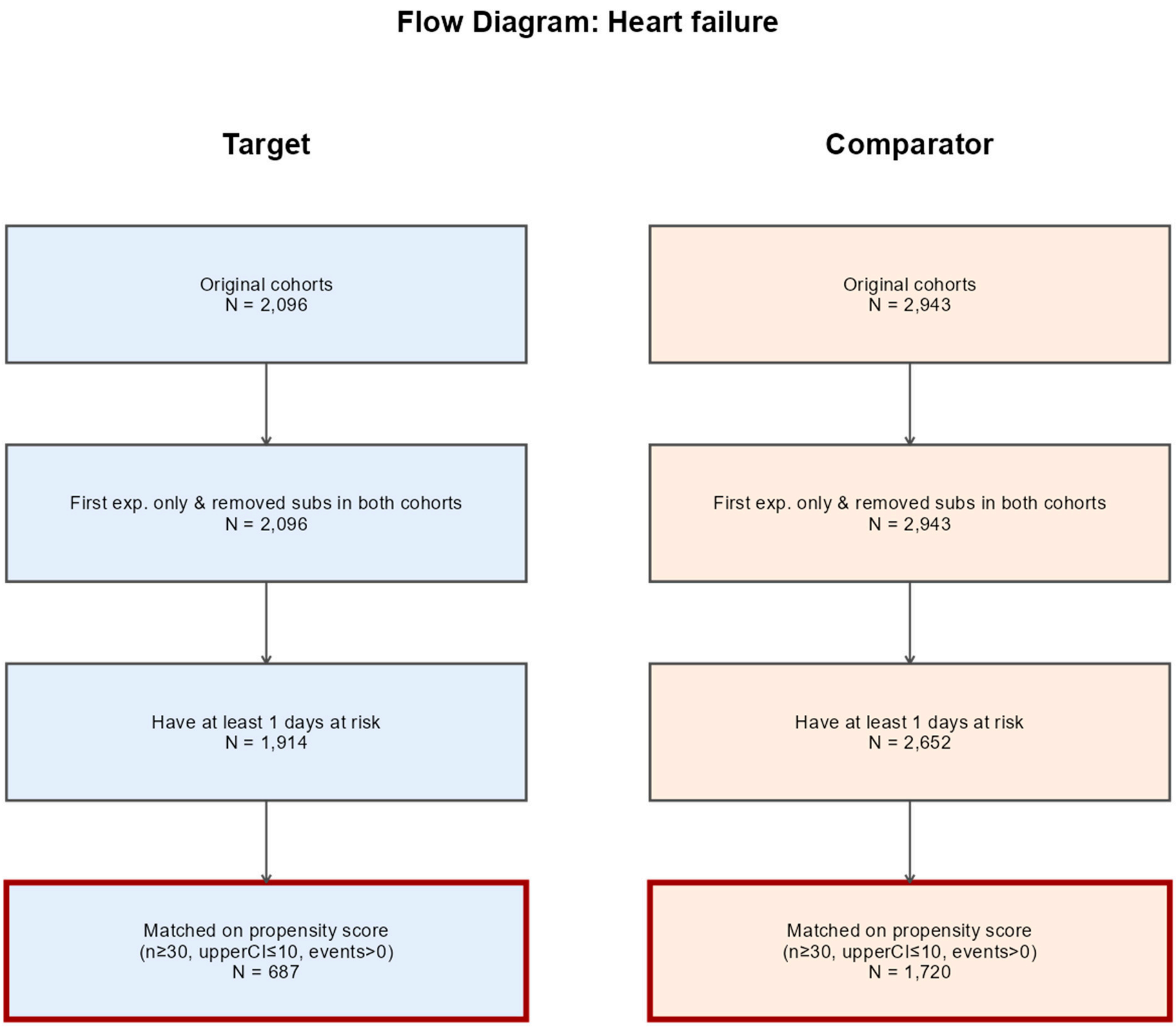

Supplementary Figure S6c. Flow Diagrams (Escitalopram vs. Trazodone), Heart failure

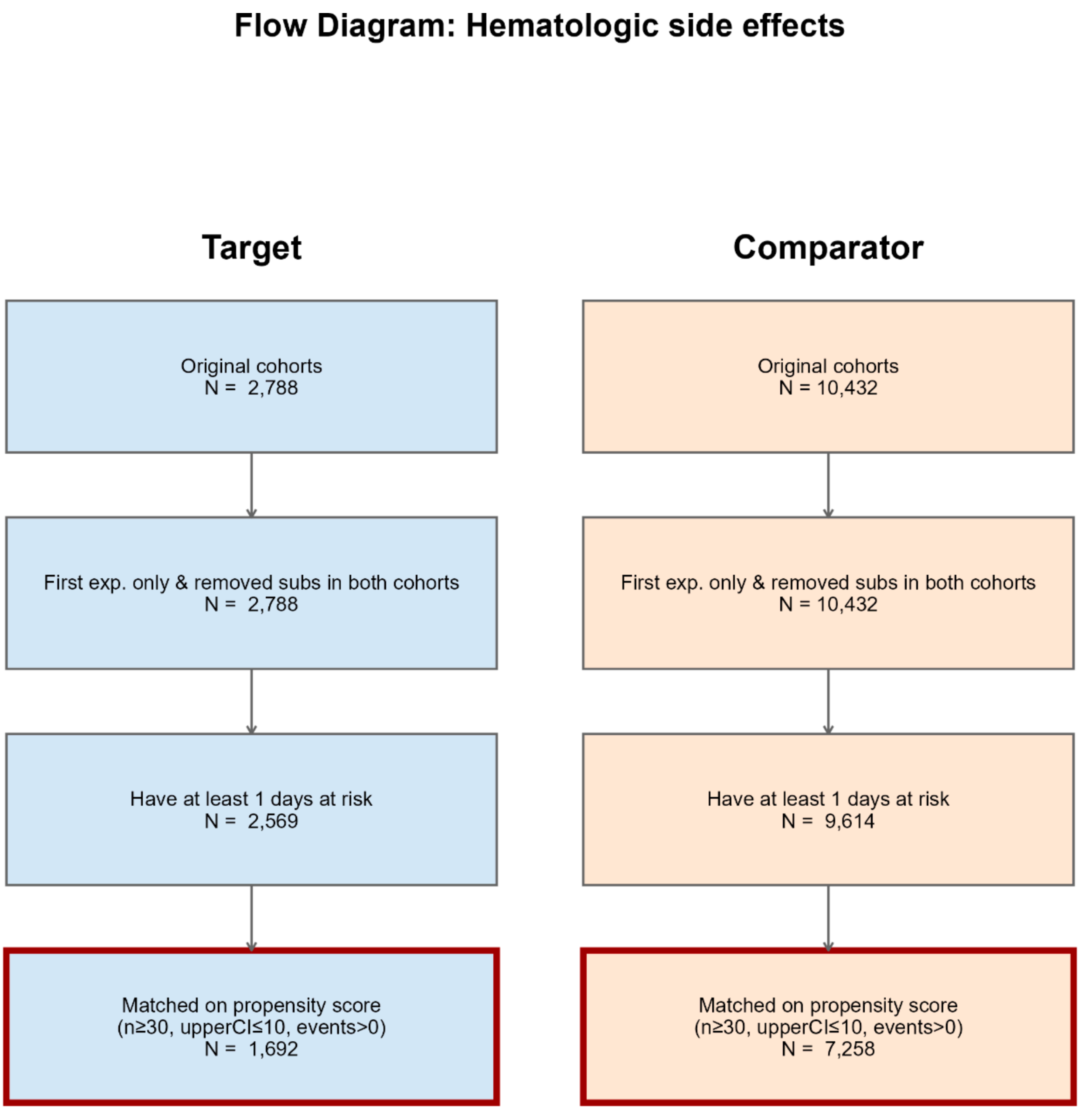

Supplementary Figure S6d. Flow Diagrams (Escitalopram vs. Trazodone), Hematologic side effects

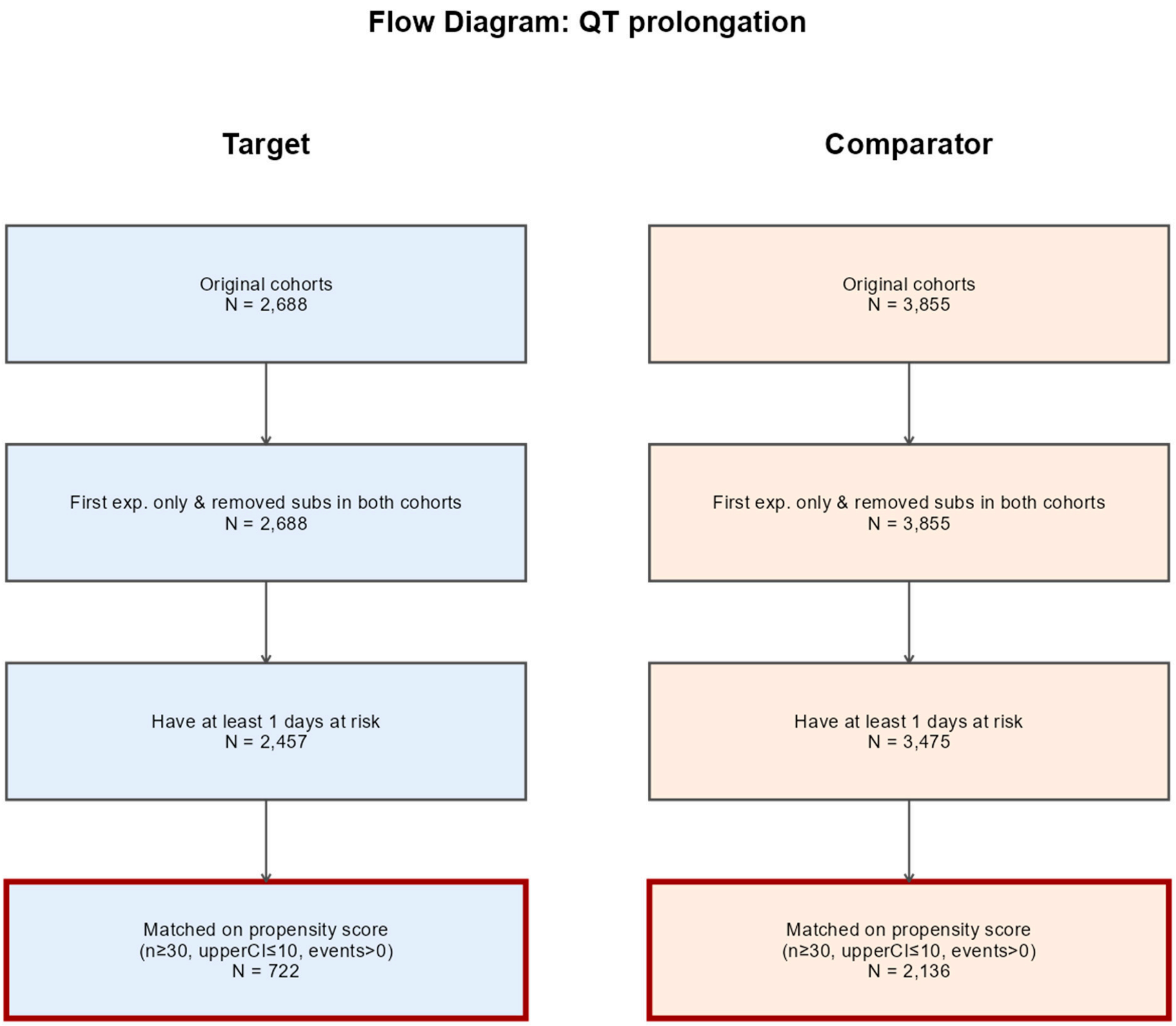

Supplementary Figure S6e. Flow Diagrams (Escitalopram vs. Trazodone), QT prolongation

Supplementary Figure S7. Forest plot of hazard ratios for multiple clinical outcomes comparing escitalopram and sertraline users in the 5-year time-at-risk (TAR).

Each point estimate represents the pooled hazard ratio (HR), and horizontal lines indicate 95% confidence intervals. The vertical line at HR = 1.0 indicates no difference between groups.

Outcome	Total N	I^2	τ^2	HR	95% CI
Atrial fibrillation	929	0%	0.0000	1.51	[0.69; 3.32]
Cardiac arrest	1,175	0%	0.0000	1.22	[0.51; 2.92]
Circulatory system	1,501	0%	0.0000	1.04	[0.82; 1.32]
Gastrointestinal system	1,123	0%	0.0000	1.26	[0.95; 1.68]
Heart failure	1,166	0%	0.0000	0.85	[0.42; 1.69]
Hematologic side effects	1,692	16%	0.0480	1.30	[0.87; 1.96]
Hypersensitivity	502	0%	0.0000	0.83	[0.24; 2.90]
Psycho-neurological system	941	0%	0.0000	0.77	[0.56; 1.05]
QT prolongation	1,273	0%	0.0000	1.75	[1.10; 2.78]

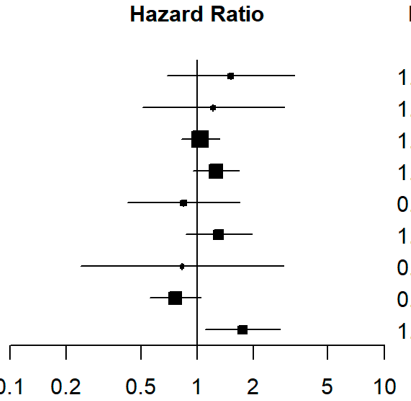

Supplementary Figure S8. Forest plot of hazard ratios for multiple clinical outcomes comparing escitalopram and trazodone users in the 5-year time-at-risk (TAR).

Each point estimate represents the pooled hazard ratio (HR), and horizontal lines indicate 95% confidence intervals. The vertical line at HR = 1.0 indicates no difference between groups.

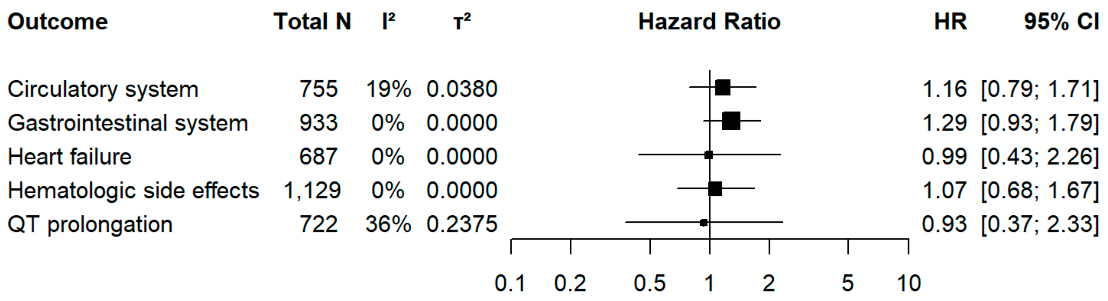

Supplementary Figure S9(a-c). Sensitivity and Subgroup Analysis Using a 0-Day Lag Time-at-Risk, by Sex and Age

As a sensitivity analysis for the primary 60-day lag time-at-risk (TAR) definition, hazard ratios were additionally estimated using a 0-day lag (i.e., the risk window began on the day of treatment initiation without a lag period), further stratified by sex (male vs. female) and age group (<65 vs. ≥65 years) within each drug comparison. This allows assessment of whether excluding the 60-day protopathic-bias buffer meaningfully changes the safety signal, and whether any such change is concentrated in a particular sex or age subgroup.

Supplementary Figure S9a. Sensitivity Analysis Using a 0-Day Lag Time-at-Risk

Escitalopram vs Sertraline

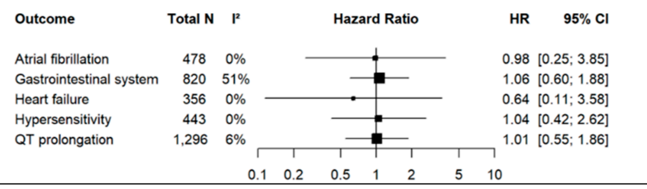

Escitalopram vs Trazodone

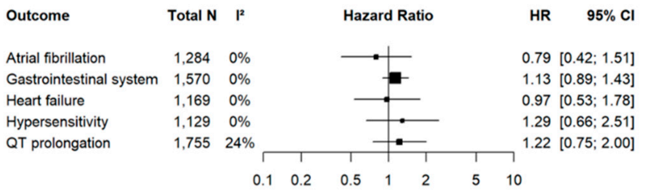

Supplementary Figure S9b. Subgroup Analysis by Sex

Analysis: Escitalopram vs Sertraline — Outcome: Gastrointestinal system

Male

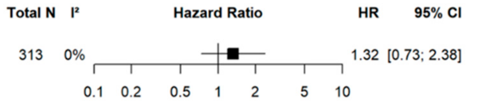

Female

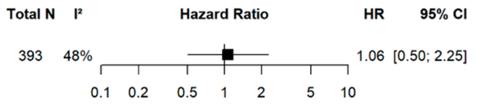

Analysis: Escitalopram vs Trazodone — Outcome: Gastrointestinal system

Male

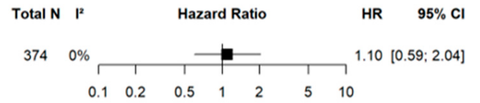

Female

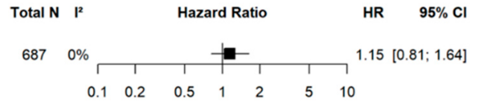

Supplementary Figure S9c. Subgroup Analysis by Age

Analysis: Escitalopram vs Sertraline — Outcome: Gastrointestinal system

Age 65 and older

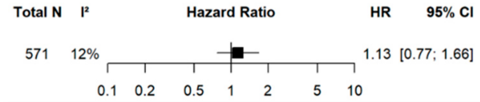

Under age 65

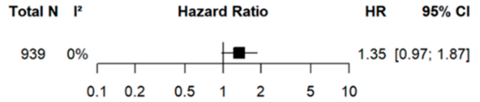

Analysis: Escitalopram vs Trazodone — Outcome: Gastrointestinal system

Age 65 and older

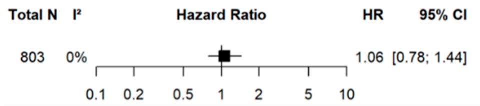

Under age 65

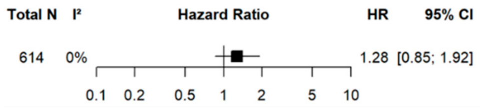

Supplementary Figure S10(a-d). Forest plot of hazard ratios across 1-, 3-year Time-at-Risk Window

Pooled hazard ratios for each safety outcome and drug comparison, estimated using 1-year, 3-year time-at-risk (TAR) windows following the 60-day lag period.

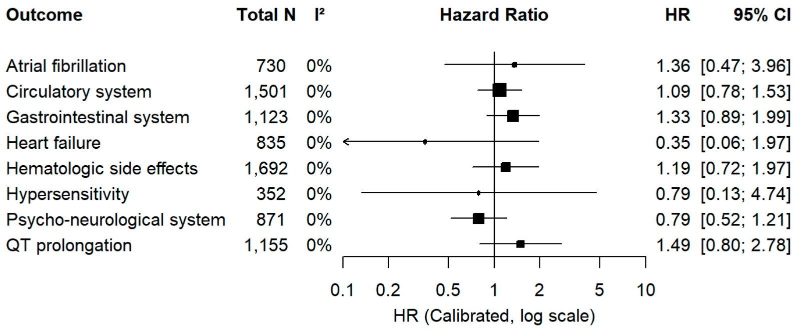

Figure S10a. Escitalopram vs Sertraline: 1-year TAR

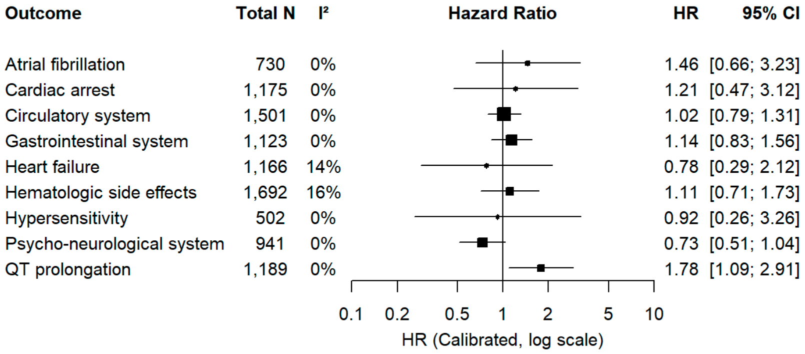

Figure S10b. Escitalopram vs Sertraline: 3-year TAR

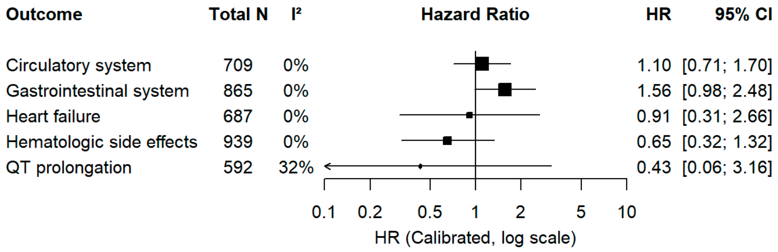

Figure S10c. Escitalopram vs Trazodone: 1-year TAR

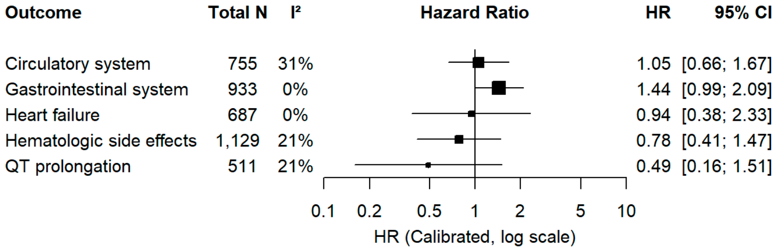

Figure S10d. Escitalopram vs Trazodone: 3-year TAR

Supplementary Figure S11(a-b). Sensitivity Analysis Restricted to the 2017–2026 Calendar Period

Pooled hazard ratios (uncalibrated) recalculated after restricting the study population to patients with an index date between 1 January 2017 and 14 January 2026, compared with the primary (1999–2026) analysis. The circulatory-system and neuropsychiatric-disorder categories are represented here by their individual component conditions (escitalopram vs. sertraline: tachycardia, tremor; escitalopram vs. trazodone: bradycardia, tachycardia).

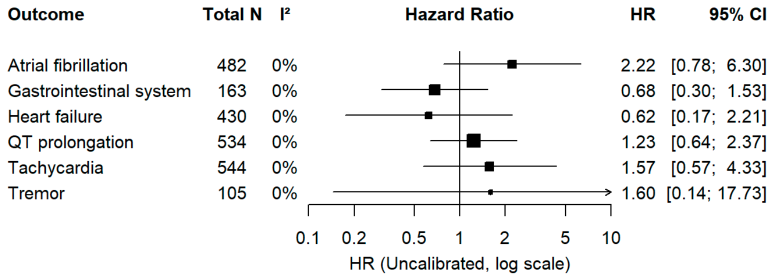

Supplementary Figure S11a. Escitalopram vs Sertraline

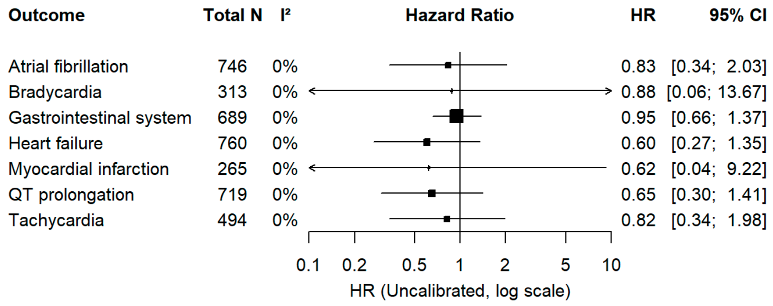

Supplementary Figure S11b. Escitalopram vs Trazodone

Supplementary Figure S12. Sensitivity Analysis Excluding Hospitals with Inaccessible Covariate Result

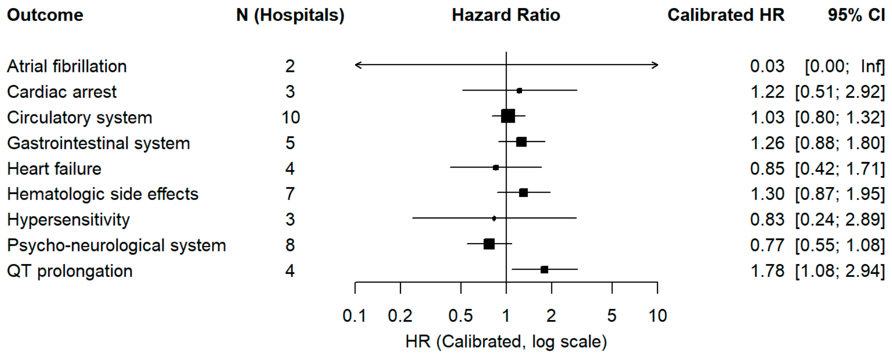

Supplementary Figure S12a. Escitalopram vs Sertraline

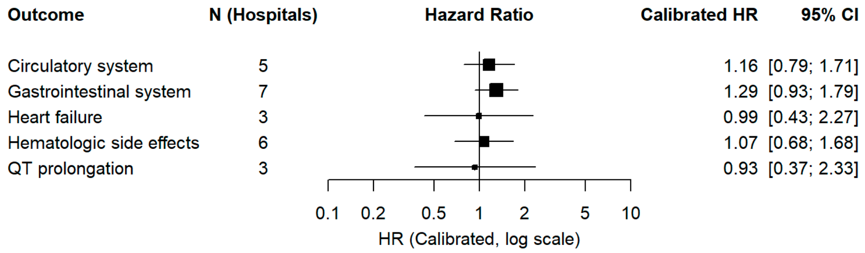

Supplementary Figure S12b. Escitalopram vs Trazodone

Supplementary Figure S13. Sensitivity Analysis Without the Confidence-Interval-Width Hospital-Exclusion Criterion

Pooled hazard ratios recalculated without applying the upper 95% confidence limit ≤10 hospital-exclusion criterion.

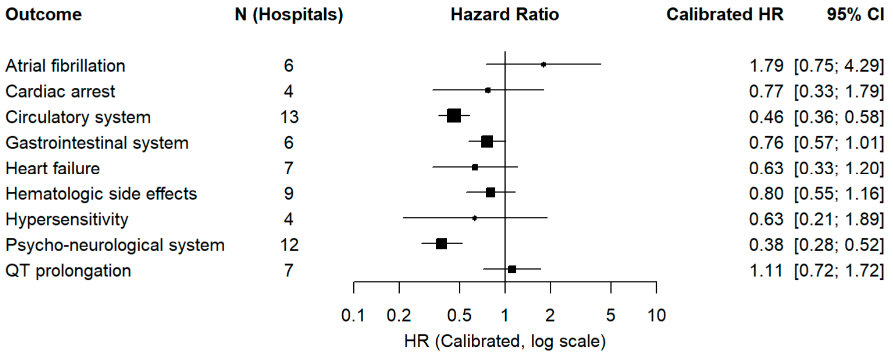

Supplementary Figure S13a. Escitalopram vs Sertraline

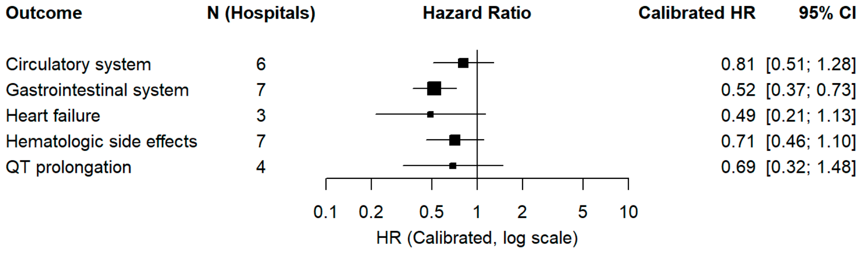

Supplementary Figure S13b. Escitalopram vs Trazodone

Supplementary Figure S14(a-b). Empirical Calibration Diagnostics for Negative Control Outcomes

P-value calibration plots for the 167 pre-specified negative control outcomes used in the empirical calibration described in the Methods. For each drug comparison, the plot compares the empirical distribution of p-values across negative controls (solid line) against the theoretical uniform distribution expected under the null hypothesis of no association (dashed line); systematic deviation of the empirical curve from the diagonal reference line indicates residual bias not accounted for by propensity score matching, which was subsequently used to derive calibrated hazard ratios for the nine safety outcomes.

Supplementary Figure S14a. Calibration Plot, Escitalopram vs Sertraline

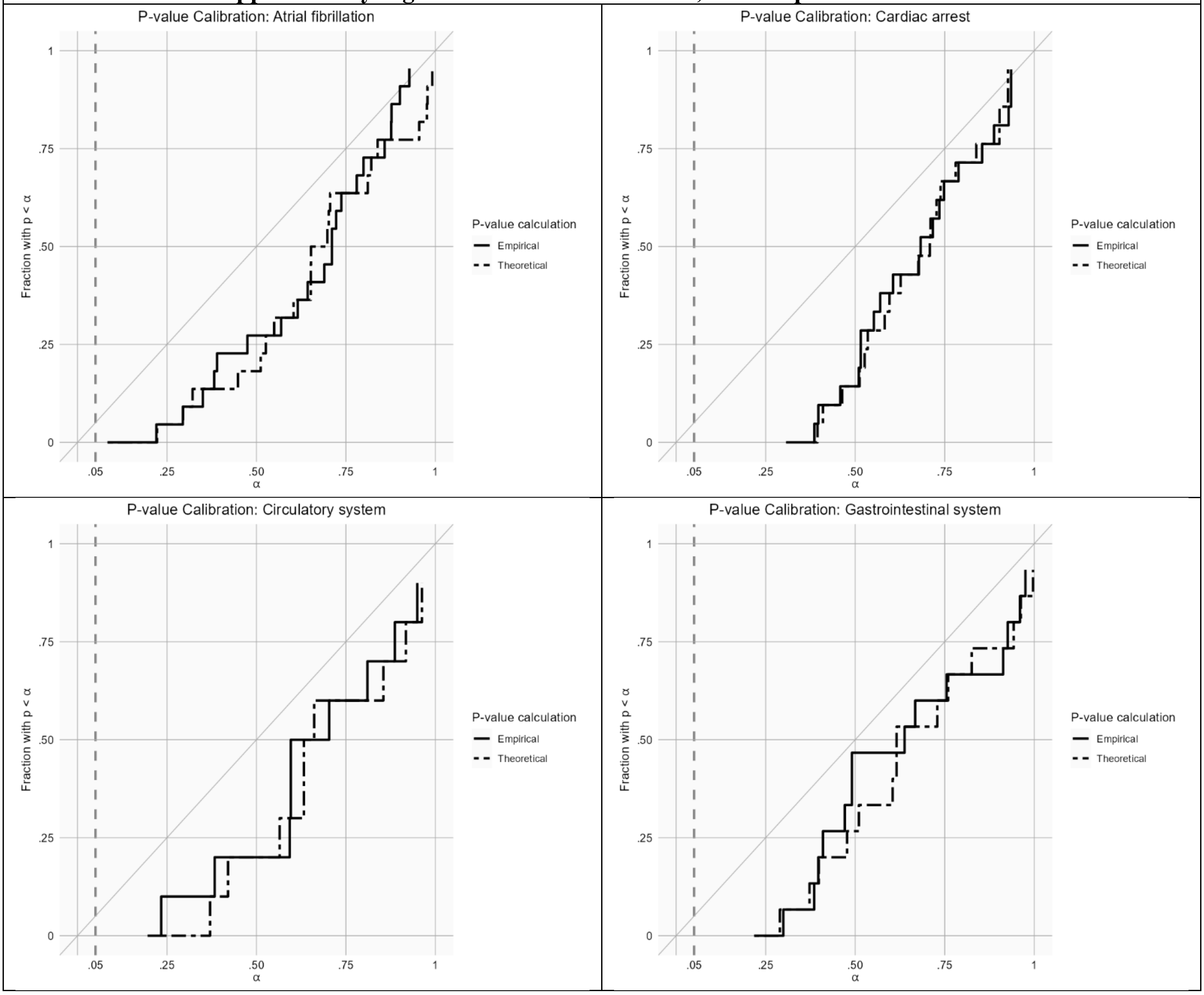

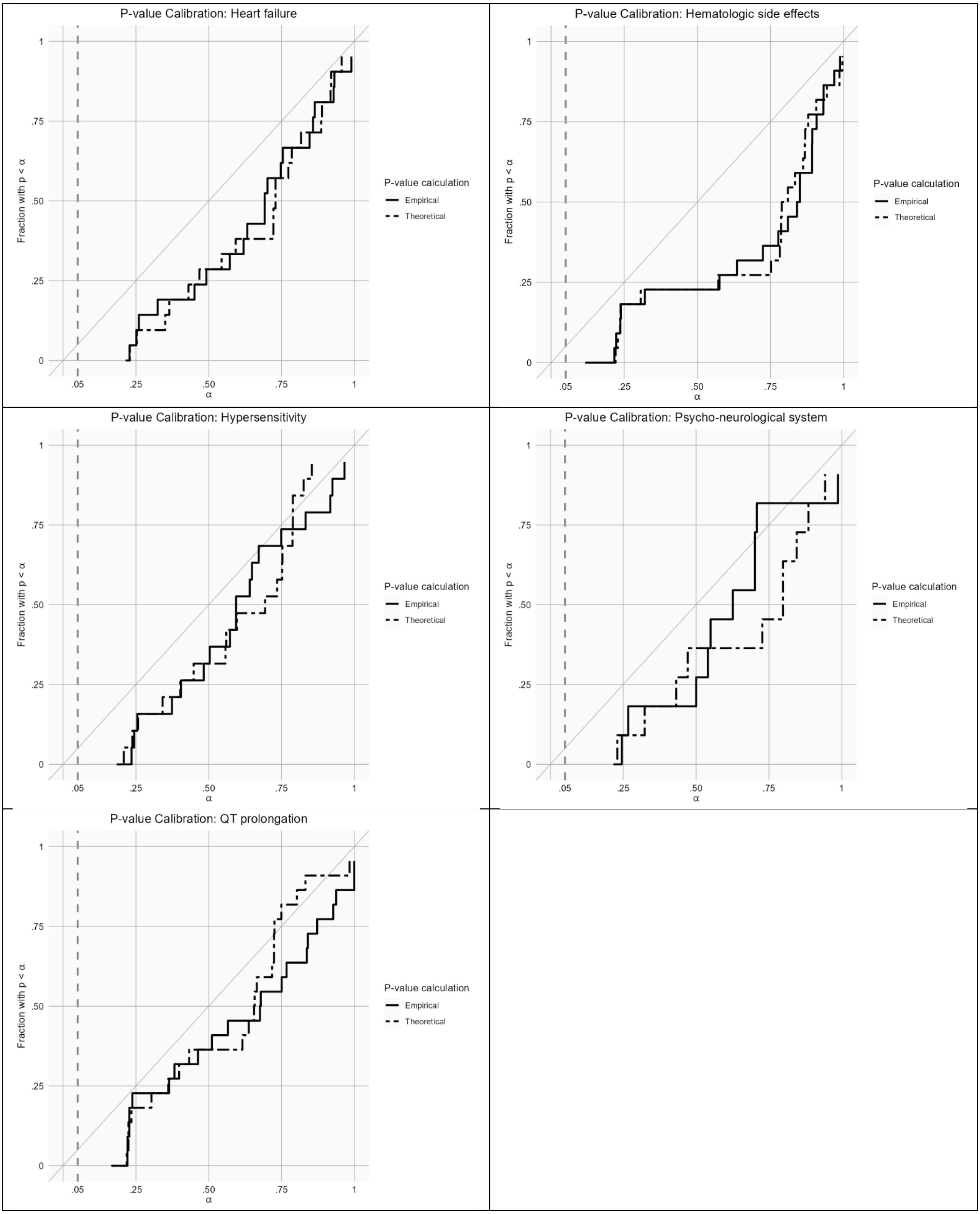
P-value Calibration: Heart failure
P-value Calibration: Hematologic side effects
P-value Calibration: Hypersensitivity
P-value Calibration: Psycho-neurological system
P-value Calibration: QT prolongation
Fraction with p < α
α
0
.25
.50
.75
1
.05
P-value calculation
Empirical
Theoretical

Supplementary Figure S14b. Calibration Plot, Escitalopram vs Trazodone

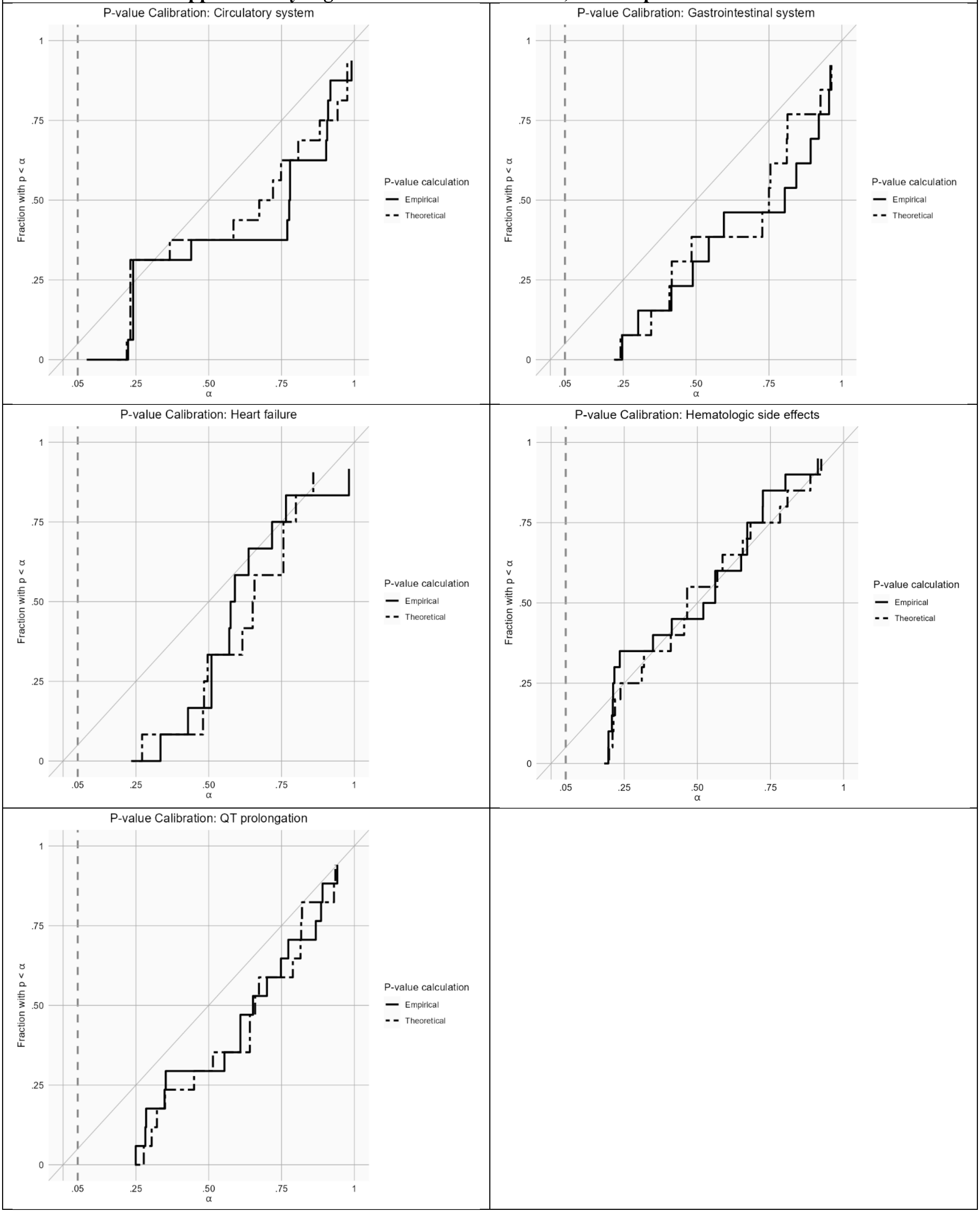

Supplement: Supplementary file 1 [file pharmaceuticals-19-01332-s001.zip › pharmaceuticals-4415433-supplementary.pdf]
